# Cloning and Comparative Analyses of the Zebrafish Ugt Repertoire Reveal Its Evolutionary Diversity

**DOI:** 10.1371/journal.pone.0009144

**Published:** 2010-02-10

**Authors:** Haiyan Huang, Qiang Wu

**Affiliations:** 1 Key Laboratory of Systems Biomedicine (Ministry of Education), Shanghai Center for Systems Biomedicine, Shanghai Jiao Tong University, Shanghai, China; 2 State Key Laboratory of Oncogenes and Related Genes, Shanghai Cancer Institute, Shanghai Jiao Tong University, Shanghai, China; Centre de Regulació Genòmica, Spain

## Abstract

UDP-glucuronosyltransferases (Ugts) are a supergene family of phase II drug-metabolizing enzymes that catalyze the conjugation of numerous hydrophobic small molecules with the UDP-glucuronic acid, converting them into hydrophilic molecules. Here, we report the identification and cloning of the complete zebrafish *Ugt* gene repertoire. We found that the zebrafish genome contains 45 *Ugt* genes that can be divided into three families: *Ugt1*, *Ugt2*, and *Ugt5*. Both *Ugt1* and *Ugt2* have two unlinked clusters: *a* and *b*. The *Ugt1a*, *Ugt1b*, *Ugt2a*, and *Ugt2b* clusters each contain variable and constant regions, similar to that of the protocadherin (*Pcdh*), immunoglobulin (*Ig*), and T-cell receptor (*Tcr*) clusters. Cloning the full-length coding sequences confirmed that each of the variable exons is separately spliced to the set of constant exons within each zebrafish *Ugt* cluster. Comparative analyses showed that both *a* and *b* clusters of the zebrafish *Ugt1* and *Ugt2* genes have orthologs in other teleosts, suggesting that they may be resulted from the “fish-specific” whole-genome duplication event. The *Ugt5* genes are a novel family of *Ugt* genes that exist in teleosts and amphibians. Their entire open reading frames are encoded by single large exons. The zebrafish *Ugt1*, *Ugt2*, and *Ugt5* genes can generate additional transcript diversity through alternative splicing. Based on phylogenetic analyses, we propose that the ancestral tetrapod and teleost *Ugt1* clusters contained multiple *Ugt1* paralogs. After speciation, these ancestral *Ugt1* clusters underwent lineage-specific gene loss and duplication. The ancestral vertebrate *Ugt2* cluster also underwent lineage-specific duplication. The intronless *Ugt5* open reading frames may be derived from retrotransposition followed by gene duplication. They have been expanded dramatically in teleosts and have become the most abundant *Ugt* family in these lineages. These findings have interesting implications regarding the molecular evolution of genes with diversified variable exons in vertebrates.

## Introduction

Natural selection plays an essential role in the evolution of vertebrate genomes. At the molecular level, DNA duplication provides important genetic materials upon which Darwinian positive selection can act. Vertebrate genomes contain a unique set of gene clusters that are organized into variable and constant regions. These gene clusters include the immunoglobulin (*Ig*), T-cell receptor (*Tcr*), Protocadherin (*Pcdh*), and UDP-glucuronosyltransferase (*Ugt*) genes [1]. Their variable region contains a tandem array of highly similar exons which are of about the same length. Each of the variable exons is combined with a single set of downstream constant exons to generate enormous molecular diversity required for the survival of the organisms. In the acquired immune system, DNA rearrangement, somatic mutation, and positive selection of the *Ig* and *Tcr* clusters provide unlimited diversity for defense against foreign antigens and for protection of our body from viral infections [2], [3]. Similarly, alternative splicing, gene conversion, and adaptive selection of the *Pcdh* clusters generate enormous diversity for the construction of trillions of specific neuronal connectivity in the central nervous system [4]–[7].

The human *Pcdh α* and *γ* clusters each contain more than a dozen variable exons and a common set of three constant exons [4]. Each “variable” exon is separately spliced to the set of downstream “constant” exons within each cluster to generate diverse functional mRNAs [4]. Each variable exon is preceded by a distinct promoter and promoter choice determines which variable exon is included in a *Pcdh* mRNA [5]. This unusual genomic organization of *Pcdh* clusters may provide a molecular foundation for generating enormous cellular diversity and complex neural connectivity in the brain.

The *Ugt* cluster encodes a diverse set of enzymes required for drug clearance, detoxification of xenobiotics, and metabolism of endobiotics, including phenolic compounds, environmental toxins, bilirubin, steroids, and bile acids [8]. *Ugt1* and *Ugt2* are the two *Ugt* families identified in mammals [9]–[11]. The mammalian *Ugt1* clusters are organized into variable and constant regions, similar to the organization of the *Pcdh* clusters [1], [12], [13]. Each variable exon is preceded by its own promoter and is separately spliced to a common set of four downstream constant exons. Each variable exon encodes a signal peptide and the amino-terminal aglycone-recognition domain. The constant exons encode a highly conserved donor-binding domain, which binds the UDP-glucuronic acid (UDPGA), and the carboxyl-terminal endoplasmic-reticulum(ER)-anchoring transmembrane segment. Therefore, the encoded family of diverse Ugt1 enzymes can catalyze the conjugation of a vast number of lipophilic xenobiotics and endobiotics with the UDP-glucuronic acid. This glucuronidation reaction converts hydrophobic aglycones to water-soluble glucuronides and enhances their excretion from the body [8], [11].

Mammalian and avian genomes contain a single *Ugt1* cluster divided into two major groups: constant-proximal bilirubin group and constant-distal phenol group [13], [14]. Members within the bilirubin or phenol group are highly similar and appear to be duplicated recently. Consistent with the whole genome duplication (WGD) in the teleost fishes [15]–[17] and similar to the duplication of the teleost *Pcdh* clusters [6], [7], [18], [19], the zebrafish *Ugt1* cluster has been duplicated into *Ugt1a* and *Ugt1b* clusters, each organized into variable and constant regions [14], [20]. Both clusters span a region of about 35 kb genomic sequences, much smaller than mammals and avians. Members of the zebrafish *Ugt1* clusters do not display orthologous relationships to those of the mammalian and avian clusters [14], [20].

Members of the *Ugt2* family are clustered in one locus in human, mouse and rat, and are probably evolved by duplication of the entire gene [14]. The human *UGT2* cluster includes three *UGT2A* genes and twelve *UGT2B* genes, five of which are pseudogenes [10], [11], [14], [21]. In contrast to the *Ugt1* family, each *Ugt2b* gene consists of six exons. The exons 1 and 2 of the *Ugt2b* genes correspond to one variable exon of the *Ugt1* cluster. Similar to the *Ugt1* cluster, *Ugt2a1* and *Ugt2a2* genes are organized into variable and constant regions, which contain two variable exons and five constant exons, respectively [9], [14].

The human *UGT* genes play important roles in drug metabolism. For example, the *UGT1A1* gene is essential in metabolizing the colorectal cancer drug irinotecan. Thus, the *UGT1A1* genotyping has been used to determine the personalized irinotecan dosage for the treatment of colorectal cancer patients [22]. As in humans, each nonhuman vertebrate species lives in different ecological niches and has distinctive dietary. For example, many fish populations thrive at unique aquatic environments. Thus, each organism interacts with unique sets of environmental “drugs” or toxins. Therefore, each species might evolve a unique set of *Ugt* genes to detoxify them. Recently, zebrafish emerged as an important model organism in the toxicological research [23]. They are small, fecund, and morphologically and physiologically similar to mammals. In addition, they have transparent, externally developing embryos that facilitate their usages in developmental toxicology [23].

In this report, we cloned the complete zebrafish *Ugt* gene repertoire, which encodes at least 40 distinct yet highly similar enzymes. In addition, sequencing their full-length coding regions revealed many novel splice variants. To gain insight into the *Ugt* evolution, we performed comprehensive comparative analyses of the *Ugt* repertoire in teleosts and lower tetrapods. We found that the vertebrate *Ugt1* clusters have undergone lineage-specific gene loss and duplication, while vertebrate *Ugt2* clusters have undergone only lineage-specific gene duplication. Finally, we discovered a novel class of genes, designated *Ugt5*, whose entire protein sequences are encoded by single large exons. Interestingly, the vertebrate *Ugt5* genes are only found in teleosts and amphibians, and have been extensively expanded in teleosts.

## Results and Discussion

### Identification and Cloning of the Complete Zebrafish *Ugt* Gene Repertoire

**Figure 1:**
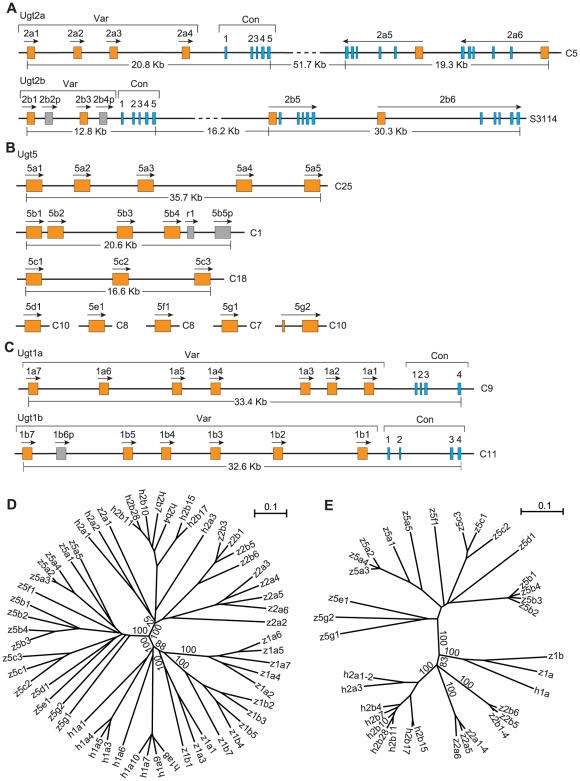
The repertoire of the zebrafish *Ugt* genes. Shown are the genomic organizations of the zebrafish *Ugt 2a* and *2b* clusters (A), the *Ugt5* genes (B), and the *Ugt 1a* and *1b* clusters (C). Orange boxes represent variable exons or *Ugt5* exons, and blue boxes represent constant exons. Gray boxes represent pseudogenes (p) or relics (r). The approximate length for each cluster is also shown. Transcription directions are indicated by an arrow above each gene. Chromosomal or scaffold locations are shown on the right by the letter “C” representing chromosome or “S” representing scaffold. Var, variable; Con, constant; Kb, kilobase pairs. The phylogenetic trees are based on the polypeptide sequences encoded by the zebrafish (z) and human (h) variable regions (D) or constant regions (E). The trees are unrooted. The tree branches are labeled with the percentage support on the basis of 1,000 bootstrap replicates. Only bootstrap values (>50%) of the major nodes are shown. The scale bar equals a distance of 0.1.

We identified 45 zebrafish *Ugt* genes through a combination of iterative BLAST searches of genomic sequences with comprehensive cloning of *Ugt* cDNA sequences (Tables S1 and S2). These genes constitute the complete repertoire of zebrafish *Ugt* genes (Fig. 1). They include two duplicated *Ugt2* clusters (Fig. 1A), 17 single-exon *Ugt5* genes (Fig. 1B), and two duplicated *Ugt1* clusters (Fig. 1C). They are located in at least ten different chromosomes. Five of them are pseudogenes (Fig. 1 and data not shown). The other 40 genes are all transcribed in the adult zebrafish as we have successfully cloned all of their full-length coding sequences. All of the encoded Ugt proteins have the typical two-Rossmann-fold-domain structures that bind donor and acceptor molecules.

We previously predicted four zebrafish *Ugt2a* (*2a1* to *2a4*) genes that are organized in a cluster containing variable and constant regions [14]. The variable region contains four highly similar exons that are organized in a tandem array. The constant region contains a set of five small exons located downstream of the variable region. We cloned the full-length coding sequences for all of the four zebrafish *Ugt2a* genes and confirmed that each of the four *Ugt2a* variable exons is separately spliced to the same set of downstream constant exons (Fig. 1A). In addition, we identified two additional *Ugt2a* (*2a5* and *2a6*) genes that are located downstream of the tandem-arrayed *Ugt2a* genes. Cloning of their full-length coding region and comparing them with the zebrafish genomic sequences revealed that both the *Ugt2a5* and *Ugt2a6* genes are organized into six exons and that they do not share any constant exon. Their first exons are highly similar to the variable exons of the tandem-arrayed *Ugt2a* genes. The next five exons are each similar to the five respective constant exons of the tandem-arrayed *Ugt2a* genes. However, the transcriptional directions are opposite to that of the tandem-arrayed *Ugt2a* genes (Fig. 1A).

We identified a novel cluster of zebrafish *Ugt2* genes. This *Ugt2b* cluster contains a variable region of four variable first exons, *2b1* to *2b4*, and a constant region with a set of five downstream constant exons (Fig. 1A). Cloning and sequencing of the full-length coding sequences revealed that, similar to the *Ugt2a* (*2a1* to *2a4*) cluster, each of the *Ugt2b* variable exons is separately spliced to the common set of downstream constant exons. However, the *Ugt2b2* and *Ugt2b4* genes appear to be pseudogenes because their variable exons are frame-shifted due to insertions. This cluster of *Ugt2b* genes is followed by two single *Ugt* genes: *Ugt2b5* and *Ugt2b6* (Fig. 1A). The *Ugt 2b5* and *2b6* genes contain six exons, each of which is similar to the corresponding exons in the tandem-arrayed *Ugt2b* genes. Contrary to the *Ugt2a* cluster, the transcriptional directions of the *Ugt5b* and *Ugt6b* genes are the same as the tandem-arrayed *Ugt2b* genes. Finally, we identified a pseudogene, *Ugt2b7p*, which is located in the same scaffold as that of the *Ugt2b* cluster (data not shown).

We identified 18 unusual *Ugt*-like genes, designated *Ugt5*, located in six different chromosomes. The encoded protein sequences show high similarity to that of the Ugt1 and Ugt2 enzymes. Each predicted Ugt5 protein contains an acceptor-binding domain preceded by an N-terminal signal peptide, and a donor-binding domain followed by an ER-anchoring transmembrane segment. Each Ugt5 protein also contains the essential catalytic histidine and aspartic acid residues [14] in its acceptor-binding domain. However, we cannot rule out the possibility that these proteins might use donor sugars other than that of the Ugt1 and Ugt2 enzymes.

In contrast to multiple coding exons of the *Ugt1* and *Ugt2* genes, the open reading frame of each of the *Ugt5* genes (except *Ugt5g2*) is encoded by a single large exon (Fig. 1B). Five of them, *Ugt 5a1* to *5a5*, are clustered on the chromosome 25. Similarly, the five *Ugt5b* (*5b1* to *5b5*) genes are clustered on the chromosome 1; however, the *Ugt5b5* gene has four nonsense mutations and two frameshift mutations and appears to be a pseudogene. Nevertheless, it is transcribed because we have cloned its cDNA. Three of the *Ugt5c* genes, *5c1* to *5c3*, are clustered on the chromosome 18. The other four *Ugt5* genes, *5d1*, *5e1*, *5f1*, and *5g1*, are nonclustered single genes. Finally, we found that the *Ugt5g2* gene contains two coding exons (Fig. 1B).

We previously identified two duplicated zebrafish *Ugt1* clusters, *1a* and *1b*. Each of these clusters contains a variable region and a constant region [14]. We predicted that each of the *Ugt1* variable exons is alternatively spliced to a single set of constant exons. We have cloned the full-length coding sequences of all of the members of the *Ugt 1a* and *1b* clusters and confirmed that each member of the zebrafish *Ugt 1a* and *1b* clusters is separately spliced to the respective set of constant exons (Fig. 1C).

To examine the evolutionary relationship of the zebrafish and human *Ugt* genes, we reconstructed their phylogenetic trees based on amino acid sequences corresponding to those encoded by the *Ugt2* variable exons (Fig. 1D) or the *Ugt1* constant exons (Fig. 1E). In both trees, the zebrafish *Ugt* superfamily is divided into three separate clades: *Ugt1*, *Ugt2*, and *Ugt5*. The zebrafish *Ugt1* and *Ugt2* genes are closely related to the human *UGT1* and *UGT2* genes, respectively. The zebrafish *Ugt5* genes form a separate branch in both trees, suggesting that the *Ugt5* genes are a novel *Ugt* subfamily that does not exist in humans (Fig. 1D and 1E).

### Additional *Ugt* Diversity Generated by Alternative Splicing

**Figure 2:**
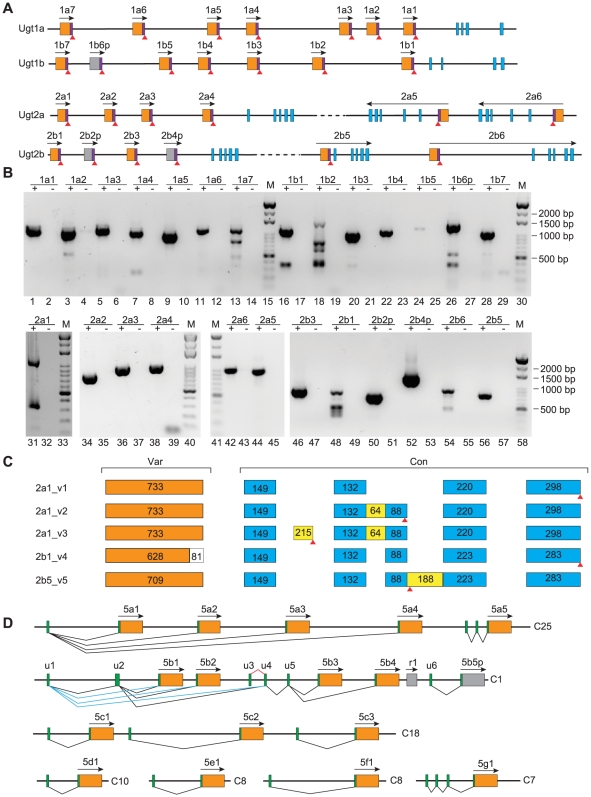
Alternative splicing of the zebrafish *Ugt* genes. (A) Each *Ugt1* or *Ugt2* member has a short-form transcript which corresponds to the variable exon (orange box) and its immediate downstream intronic sequences (purple box). Red triangles indicate the potential translational stop codons. Gray box indicates pseudogene. Transcription directions are marked by an arrow above each gene. (B) Detection of the short-form cDNA by RT-PCR and agarose electrophoresis. “+” and “−” indicate with and without reverse transcriptase, respectively. Amplification bands are detected only in “+” lanes. M: 1 kb marker. (C) Alternatively spliced variants for the *Ugt2a1*, and *Ugt 2b1* and *2b5* genes. The exon length is shown in each box. (D) Alternative splicing of the zebrafish *Ugt5* genes. Green boxes represent 5′ noncoding exons or short noncoding exonic sequences immediate upstream of the ATG codon.

In addition to the cDNAs containing both variable and constant regions, we also cloned a set of unusual short-form cDNAs from the adult zebrafish for each member of the clustered *Ugt1* and *Ugt2* genes. These short-form cDNAs correspond to the variable exons and their immediate downstream intronic sequences (Fig. 2A). Such short-form cDNAs have also been found for members of the *Pcdh α* and *γ* clusters, corresponding to the *Pcdh* variable exons and their immediate downstream intronic sequences [4], [24]. However, the physiological function for the encoded short-form *Pcdh* proteins remains to be established.

All of the *Ugt1* and *Ugt2* cDNAs have these short variants (Fig. 2B). However, we do not know whether the RNA molecules corresponding to these cDNAs are polyadenylated. In addition, we found that few EST sequences match these short-form variants of the *Ugt* cDNAs. Nevertheless, they may encode short-form polypeptides containing only the N-terminal signal peptide and the aglycone-recognition domain, but lacking the donor-binding domain and the ER-anchoring transmembrane segment. Therefore, they do not have intrinsic UDP-glucuronosyltransferase activities. However, they may form hetero- or homo-oligomers with full-length Ugt proteins. It has long been suggested that Ugts functions in ER membranes as dimers in monoglucuronide formation, and may form tetramers in diglucuronide formation [25], [26]. The amino terminal domain has been suggested to be involved in human UGT2B1 dimerization [27]. Alternatively, the short *Ugt* RNA variants may play some roles in the regulation of the *Ugt* gene expression and never encode any polypeptide. For example, they may be processed to small regulatory RNA molecules such as miRNAs. However, we could not find any miRNA sequence in the public miRNA databases matching these short *Ugt* RNA variants. Further experiments need to be performed to elucidate their physiological functions.

Each of the full-length *Ugt2* mRNAs is generated by splicing of one variable exon to the set of five constant exons (Figs. 1A and 2C). Interestingly, cloning the full-length coding sequences revealed at least four additional transcripts (v2 to v5) (Fig. 2C). *Ugt2a1*_*v2* is an intron-retention transcript that retained the entire 64-nucleotide intronic sequences between the constant exons 2 and 3. *Ugt2a1*_*v3* contains an additional 215-nucleotide alternative exon within the intronic sequences between the constant exons 1 and 2. Cloning of the *Ugt2b1*_*v4* transcript revealed an alternative 5′ splice site within the variable exon that splices directly to the first constant exon to generate an 81-nucleotide shorter mRNA molecule. Finally, we cloned a cDNA molecule (*Ugt2b1_v5*) that retained the entire 188-nucleotide intronic sequences between the constant exons 3 and 4. All of these four types of alternatively spliced transcripts may encode short-form Ugt proteins (Fig. 2C). Interestingly, the Guillemette's group has recently identified a novel alternative exon at the 3′ end of the mammalian *Ugt1* cluster which generates a new set of Ugt1a isoforms [28], [29]. These isoforms can decrease the enzymatic activity of the normal Ugt1 proteins [28], [29].

The transcription start site of each member of the *Ugt1* and *Ugt2* clusters has been mapped to the immediate upstream of the corresponding variable exon. Each variable exon of these two *Ugt* gene clusters is associated with its own promoter. Therefore, these *Ugt1* and *Ugt2* genes belong to a large class of genes whose expression patterns are determined by their respective promoters of multiple variable first exons [1]. By contrast, sequencing of the cloned *Ugt 5a1* to *5a4* cDNAs and comparing them with the genomic DNA sequences revealed that a common 5′ noncoding exon is spliced to each of the four mutually exclusive coding exons (Fig. 2D). Therefore, the *Ugt 5a1* to *5a4* share a common upstream noncoding exon and are actually transcribed from a single promoter (Fig. 2D). Each of these four transcripts appears to be separately polyadenylated. For example, two clones (BC109447 and BC124407) that correspond to two of these four transcripts contain poly(A) tails. Therefore, this cluster of *Ugt5* genes belongs to a class of genes that are alternatively polyadenylated [30]. Interestingly, their downstream paralogous *Ugt5a5* gene has two different 5′ noncoding exons (Fig. 2D).

The genomic organization of the *Ugt 5b1* to *5b4* is the most complex. In addition to the first 5′ noncoding exon *u1*, we also found an alternative 5′ noncoding exon *u2* (Fig. 2D) that can be included into each of the *Ugt5b1* to *5b4* mRNAs. This second 5′ noncoding exon is 364 bp in length and is located at about 3.4 kb downstream of the first 5′ noncoding exon *u1* and about 1.7 kb upstream of the *Ugt5b1* coding region. The *u2* exon can also be skipped in a second set of *Ugt5b* transcripts. Thus, the exclusion or inclusion of the exon *u2* generates two sets of mRNA variants for each member of the *Ugt 5b1* to *5b4* cluster. In addition, we discovered two additional constitutively included 5′ noncoding exons (Fig. 2D, *u4* and *u5*) located between the protein-coding region of the *Ugt 5b2* and *5b3*. The noncoding exon *u1* is spliced to the noncoding exon *u4*, followed by the noncoding exon *u5* and the protein-coding exon to generate a set of the *Ugt 5b3* and *5b4* mRNAs. Alternatively, inclusion of the noncoding exon *u2* generates a second set of the *Ugt 5b3* and *5b4* mRNAs that are 364-bp longer. Finally, we identified a novel 5′ noncoding exon (Fig. 2D, *u3*) that is located at the immediate upstream of the noncoding exon *u4*, potentially generating the third set of the *Ugt 5b3* and *5b4* mRNAs. Indeed, cloning and sequencing the *Ugt 5b3* and *5b4* cDNAs (Table S2, Ugt5b3_v3 and Ugt5b4_v3) confirmed that *u3* is spliced to *u4*, followed by *u5* and the protein-coding exon. In total, three sets of different mRNAs could be generated by alternative splicing for these *Ugt5* genes. The downstream paralogous *Ugt5b5p* pseudogene does not share any 5′-UTR exon with *Ugt 5b1* to *5b4*, but has its own 5′ noncoding exon *u6* (Fig. 2D).

The *Ugt 5b1* to *5b4* are likely derived from one common ancestral *Ugt5* gene that has 5′ noncoding exons. This ancestral gene may have experienced multiple rounds of coding-exon duplications, generating three more duplicated *Ugt* exons (Fig. 1D and 2D). The 5′ splice sites of the upstream 5′ noncoding exons are then spliced to the 3′ splice sites of these mutually exclusive exons, thus generating molecular diversity in the 5′-UTRs of *Ugt 5b1 to 5b4*. These dynamic 5′-UTR structure might play an important role in regulating mRNA stability or their translation efficiency, thus may determine the spatial or temporal expression patterns of the *Ugt5b* genes. The *Ugt 5a1* to *5a4* cluster may evolve similarly.

The clustered *Ugt5c* genes each have its own 5′ noncoding exons. Despite of high similarity among their coding regions, their 5′ noncoding exons do not show any sequence similarity. Their introns differ sharply from each other either in length or sequences. Similar to members of the *Ugt5c* family, *Ugt 5d1*, *5e1*, and *5f1* each have its own 5′ nonconding exon. Finally, the *Ugt5g1* gene contains three distinct 5′ noncoding exons. The second and third of these noncoding exons are only 58 bp and 71 bp, respectively. The splicing of each of these 5′ noncoding exons has been confirmed by cloning and sequencing (Table S2). Similar genomic organizations have been found for other family genes. For example, the entire coding sequences of hundreds of vertebrate olfactory receptor genes are also encoded by single exons. Moreover, the olfactory receptor genes appear to have 5′ noncoding exons [31].

### Lineage-Specific Birth and Death Evolution of the Vertebrate *Ugt1* Genes

**Figure 3:**
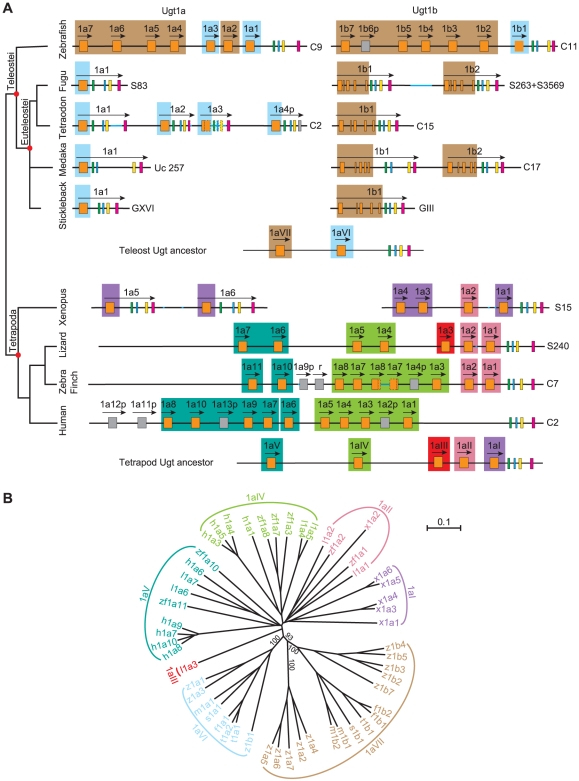
The evolution of the vertebrate *Ugt1* genes. (A) Comparison of the vertebrate *Ugt1* clusters with species names indicated on the left. The exons are indicated by boxes with different colors. Pseudogene (p) and relic (r) are indicated by gray boxes. The directions of transcription are indicated by arrows. Chromosomal or scaffold locations are shown on the right. Small gaps are represented by light blue lines and possible exons in the gaps by dotted boxes. The hypothetical ancestral teleost and tetrapod *Ugt1* clusters are shown with orthologous variable exons shaded in the same background color. (B) Phylogenetic tree of the zebrafish (z), fugu (f), tetraodon (t), medaka (m), stickleback (s), xenopus (x), lizard (l), zebra finch (zf), and human (h) *Ugt1* genes. The tree branches are labeled with the percentage support on the basis of 1,000 bootstrap replicates. Only bootstrap values (>50%) of the major nodes are shown. The trees are unrooted. The scale bar equals a distance of 0.1. The seven *Ugt1* groups (*1aI* to *1aVII*) are indicated with the same color as shaded in the panel A.

Members of the zebrafish *Ugt1* do not display orthologous relationships to those of the mammalian and avian *Ugt1* clusters [14]. To determine their evolutionary history, we annotated the complete *Ugt1* gene repertoire in other teleosts such as fugu, tetraodon, medaka, and stickleback, as well as in a wide variety of lower tetrapods. Each teleost has two *Ugt1* loci, designated *1a* and *1b* (Fig. 3). To our surprise, the *Ugt1* genes in none of these four teleosts are organized into variable and constant regions. Instead, they are single genes each organized into multiple exons (Fig. 3A). As in zebrafish, the *Ugt1a* genes in euteleosts each contain five exons. All of the corresponding exons are highly similar among members of the teleost *Ugt1a* genes.

The coding regions of the euteleosts *Ugt1a* first exons are 861 bp in length; however, the corresponding sequences in their *Ugt1b* genes are organized into five small exons (Fig. 3A). Although these *Ugt1b* genes display close relationships with the zebrafish *Ugt1b* genes in the phylogenetic tree (Fig. 3B), we cannot rule out that they may not be the zebrafish *Ugt1b* orthologs. The first exon of these euteleost *Ugt1b* genes appears to be a little longer than the corresponding *Ugt1a* region (Fig. S1). Thus, the mature proteins of the fugu *1b1* and *1b2*, tetraodon *1b1*, medaka *1b1* and *1b2*, and stickleback *1b1* could contain 34, 34, 29, 29, 27, and 42 more amino acid residues than that of the Ugt1a proteins, respectively.

Xenopus contains at least six *Ugt1* genes (Fig. 3A). The *Ugt 1a1* to *1a4* genes are organized into variable and constant regions and the *Ugt1a5* and *Ugt1a6* genes are single genes organized into five exons. The *Ugt 1a1* to *1a4* genes appear to be generated by tandem duplication of variable exons while the *Ugt1a5* and *Ugt1a6* genes seem to arise from the duplication of the entire five ancestral *Ugt1a* exons (Fig. 3A). Similar to humans, the green lizards and the zebra finches each contain a single *Ugt1* cluster, organized into variable and constant regions. Lizards contain seven *Ugt1* variable exons, much less than those in zebra finches and humans (Fig. 3A).

To determine the evolutionary history of the vertebrate *Ugt1* genes, we performed phylogenetic analyses using polypeptides (Fig. S1) encoded by the variable regions of the *Ugt1* clusters and the corresponding first exons of other *Ugt1* genes (Fig. 3B). The tree demonstrates that the vertebrate *Ugt1* variable exons can be grouped into seven clades, *Ugt 1aI* to *1aVII* (Fig. 3B), each may be derived from an ancestral variable exon. Teleosts have the clades *1aVI* and *1aVII*; while tetrapods have the clades *1aI* to *1aV* (Fig. 3A). In addition, the teleost and tetrapod *Ugt1* genes do not have any overlapping clade. Therefore, we propose that the ancestral teleost *Ugt1* cluster contained two variable exons (*1aVI* and *1aVII*) and that the ancestral tetrapod *Ugt1* cluster contained five variable exons (*1aI* to *1aV*) (Fig. 3A). Both zebrafish *Ugt 1a* and *1b* clusters maintained *1aVI* and *1aVII* after the fish-specific WGD. By contrast, the euteleosts lost *1aVII* in the *Ugt1a* cluster and lost *1aVI* in the *Ugt1b* cluster. Finally, members of the zebrafish *Ugt1* clusters expanded greatly through tandem duplication of the *1aVI* and *1aVII* variable exons (Fig. 3A).

The ancestral xenopus *Ugt1* cluster contains the *Ugt 1aI* and *1aII* variable exons. The *Ugt1aI* has been greatly expanded into five variable exons (*1a1*, and *1a3* to *la6*) (Fig. 3A). Lizards have the *1aII*, *1aIII*, *1aIV*, and *1aV* groups but have lost the other three ancestral variable exons. Zebra finches also have the ancestral *1aII* variable exon. In addition, this exon might have been duplicated before the divergence of zebra finches and lizards. Moreover, the *Ugt 1aIV* and *1aV* have also been duplicated in zebra finches and lizards. The *Ugt1aIII* was unique in lizards and might have been lost in other species. The humans have tandem duplications of *Ugt 1aIV* and *1aV* but have lost all of the other three *Ugt1* ancestral genes (Fig. 3A).

The mammalian *Ugt1* genes can be divided into constant-proximal bilirubin group and constant-distal phenol group [13], [14]. For example, human *UGT 1A1* to *1A5* genes belong to the bilirubin group and *1A6* to *1A10* belong to the phenol group. Thus, *1aIV* and *1aV* might correspond to the ancestors of the bilirubin and phenol groups, respectively (Fig. 3A). These evolutionary dynamics of the *Ugt1* clusters in different lineages of vertebrates might have contributed to their adaptation to specific habitats.

### Lineage-Specific Duplication of the Vertebrate *Ugt2* Genes

**Figure 4:**
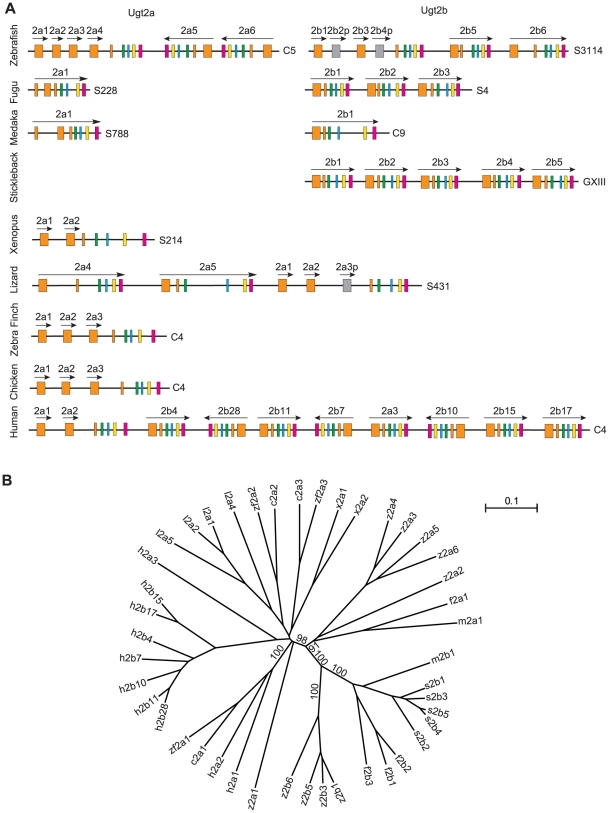
The evolution of the vertebrate *Ugt2* genes. (A) Comparison of the vertebrate *Ugt2* clusters with species indicated on the left. The exons are indicated by boxes with different colors. Pseudogenes (p) are indicated by gray boxes. The directions of transcription are indicated by arrows. Chromosomal or scaffold locations are shown on the right. (B) Phylogenetic tree of the zebrafish (z), fugu (f), medaka (m), stickleback (s), xenopus (x), lizard (l), zebra finch (zf), and human (h) *Ugt2* genes. The trees are unrooted. Bootstrap values (>50%) of only the major nodes are shown. The scale bar equals a distance of 0.1.

We have analyzed the evolution of the vertebrate *Ugt2* clusters. Zebrafish, fugu, and medaka have two unlinked *Ugt2* clusters, *2a* and *2b* (Fig. 4A). We only found one *Ugt2* cluster in the draft genomic sequences of stickleback. There may be a second stickleback *Ugt2* cluster that have not been sequenced yet. In the phylogenetic tree based on polypeptides (Fig. S2) encoded by the *Ugt2* variable exons, almost all of the vertebrate *Ugt2* display paralogous relationships (Fig. 4B). This suggests that members of the *Ugt2* clusters have undergone lineage-specific gene duplication.

Like the teleost *Ugt1* clusters, only zebrafish *Ugt 2a* and *2b* clusters contain variable and constant regions (Fig. 4A). In the zebrafish *Ugt2a* cluster, the variable exons are resulted from tandem duplication of an ancient variable exon. The variable exons *2a3* and *2a4* seem to be duplicated most recently. Similarly, the six-exon *Ugt 2a5* and *2a6* genes appear to be duplicated recently (Fig. 4B). In the zebrafish *Ugt2b* cluster, the six-exon *Ugt 2b5* and *2b6* genes are likely duplicated early. The variable exons *2b1* to *2b4* may be resulted from tandem duplication of an ancient variable exon (Fig. 4A).

The euteleost *Ugt2b* genes are single genes that are each organized into six exons. However, the euteleost *Ugt2a* genes are each organized into seven exons (Fig. 4A). The sizes of the first two *Ugt2a* exons are 231 and 490 bp in fugu, and 246 and 490 bp in medaka, respectively, corresponding to the 712∼733 bp in length of the zebrafish *Ugt2a* variable exons. The encoded amino acid sequences are highly similar to those encoded by the other teleost *Ugt 2a* and *2b* genes (Fig. S2). Compared to the variable and constant genomic organization of the zebrafish *Ugt2a* cluster, the fugu and medaka *Ugt2a* each contain only one single gene. A distinct feature of the euteleost *Ugt2* genes is that none of them share common constant exons or contain multiple variable exons (Fig. 4A).

Mammals have three *Ugt2a* and multiple *Ugt2b* genes [10], [11], [14]. The human *UGT2A* genes have orthologs in mice and rats, while the human *UGT2B* genes display paralogous relationship to those of mouse and rat [14]. Chicken and zebra finch have three *Ugt2a* variable exons (Fig. 4A). Each of these three variable exons displays strict orthologous relationships in the phylogenetic tree, indicating that their expansion occurred before the divergence of chicken and zebra finch. Lizard and Xenopus have two functional *Ugt2a* variable exons and these exons display paralogous relationships (Fig. 4B). Zebrafish has four *Ugt2a* variable exons and they display paralogous relationships (Fig. 4B). Because the variable exons encode the aglycone-recognition domains, these dynamic evolution of variable exons may be an adaptation of each organisms to its specific environmental niches.

### The *Ugt5* Genes Are Only Found in Lower Vertebrates

**Figure 5:**
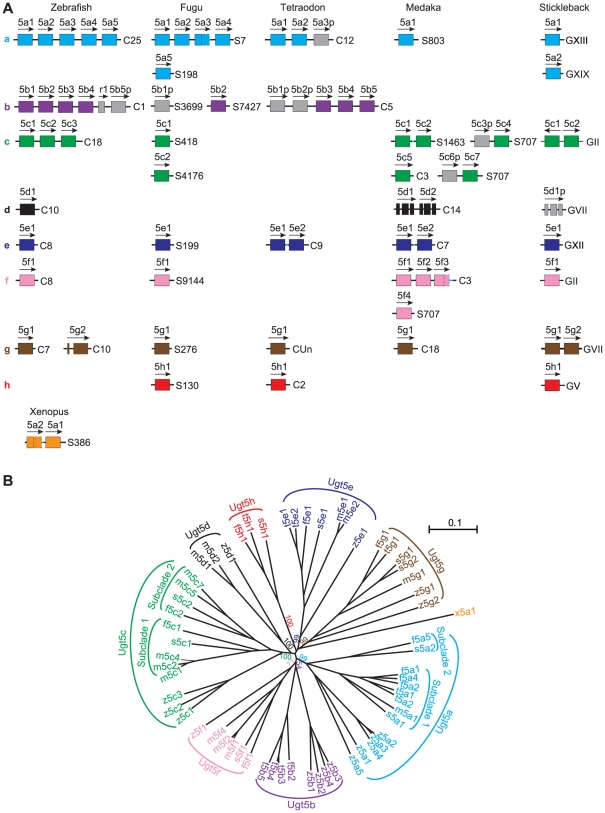
The *Ugt5* genes are expanded in teleosts. (A) Vertebrate *Ugt5* genes in teleosts and xenopus. Members of each *Ugt5* subfamily (*5a* to *5h*) are indicated by colored boxes. Pseudogene (p) and relic (r) are indicated by gray boxes. Transcription directions are indicated by arrows. Chromosomal or scaffold locations are shown on the right. Small gaps are represented by light blue lines and possible exons in the gaps by dotted boxes. (B) Phylogenetic tree of the zebrafish (z), fugu (f), tetraodon (t), medaka (m), stickleback (s), and xenopus (x) *Ugt5* genes. Genes labeled with same colors belong to the same subfamily. The trees are unrooted. Only bootstrap values (>50%) of the major nodes are shown. The scale bar equals a distance of 0.1.

The teleost *Ugt5* family can be divided into eight groups, *Ugt 5a* to *5h*, based on their sequence divergence (Fig. 5A). Members of the zebrafish *Ugt 5a*, *5b*, *or 5c* groups are clustered (Fig. 5A). All of the other members of the zebrafish *Ugt5* family are not clustered. *Ugt 5d to 5f* are single genes. The *Ugt5g* group contains two members: *5g1* and *5g2*.

One striking feature of members of the *Ugt5* family is that their entire open reading frames are encoded by single large exons. The only exception is the *Ugt5g2* gene which contains two exons that are 123 and 1482 bp in length (Fig. 5A). The zebrafish *Ugt5* family has eighteen members, representing about 40% of the total number of the zebrafish *Ugt* genes. To determine the evolutionary history of the *Ugt5* family, we searched a wide variety of vertebrate genomes. We identified *Ugt5* genes in amphibians and teleosts but could not find any *Ugt5*-like gene in reptiles, birds, and mammals (Fig. 5A).

Each teleost species has more than ten *Ugt5* genes (Fig. 5A). However, xenopus has only two *Ugt5* genes (Fig. 5A). It appears that the *Ugt5* genes decreased from teleosts to amphibians. Thus, a large number of *Ugt5* genes were lost during the transition from teleosts to amphibians. By contrast, the *Ugt1* and *Ugt2* families were expanded to generate diversity during the transition from amphibians to sauropsids and mammals.

We reconstructed a phylogenetic tree based on the full-length sequences of the encoded *Ugt5* proteins (Fig. 5B; Fig. S3). The tree shows that the teleost *Ugt5* genes form eight clades. In each clade, zebrafish *Ugt5* genes occupy a basal phylogenetic position among the teleost species. This is consistent with that zebrafish belongs to *Otocephala* while fugu, tetraodon, medaka, and stickleback belong to *Euteleostei*. All subfamilies of the zebrafish *Ugt5* genes have orthologs in other fishes. The *Ugt5a* orthologs in *Euteleostei* are grouped into two subclades. One subclade contains fugu *5a1 to 5a4*, tetraodon *5a1 and 5a2*, medaka *5a1*, and stickleback *5a1*. The other contains fugu *5a5* and stickleback *5a2* (Fig. 5B). The fugu *5a1 to 5a4* and tetraodon *5a1 to 5a3* genes are clustered, suggesting that they are resulted from tandem duplication of an ancient *Ugt5* gene. The *Ugt5b* orthologs are found in pufferfish species but not in *Smegmamorpha* (medaka and stickleback). Fugu *Ugt5b* genes have at least two members: *5b1* and *5b2*. The *5b1* gene appears to be a pseudogene because of a nonsense mutation. Tetraodon contains five tandem-arrayed *Ugt5b* genes two of which have frame-shift mutations and appear to be pseudogenes.

The *Ugt5c* and *Ugt5f* genes are present in the genomes of zebrafish, fugu, medaka, and stickleback, but are absent in the tetraodon genome. Like *Ugt5a*, the *Ugt5c* genes in euteleosts can be grouped into two subclades in the phylogenetic tree (Fig. 5B). One subclade contains the fugu *5c1*, medaka *5c1* to *5c4*, and stickleback *5c1*. The other contains the fugu *5c2*, medaka *5c5* to *5c7*, and stickleback *5c2*. Interestingly, we found that, in the medaka and stickleback genomes, the *Ugt5c* genes in the second subclade are always located directly upstream of the *Ugt5f* genes (data not shown).

The *Ugt5d* genes seem to be lost in pufferfishes (fugu and tetraodon) and duplicated in tandem in medaka. Surprisingly, the medaka and stickleback *Ugt5d* genes have three exons which are 288, 714, and 585 bp in length. The stickleback *Ugt5d* gene appears to be a pseudogene because it lacks the coding sequences for the N-terminal signal peptide. *Ugt5e* and *Ugt5g* exist in all of the five teleosts. The tetraodon and medaka *Ugt5e* and stickleback *Ugt5g* genes have been duplicated recently. Other fishes only contain one copy of the *Ugt5e* and *Ugt5g* gene. Finally, *Ugt5h* is absent in zebrafish and medaka but present as single copies in the fugu, tetraodon, and stickleback genomes.

### A Possible Mechanism for the Expansion of *Ugt5* Genes in Teleosts

How did so many *Ugt5* genes occur in the teleost genomes? We posit that retrotransposon might play a role in their expansion. If a retrotransposon inserts into a site near an ancient multi-exon *Ugt* gene, such as the ancestral *Ugt1* or *Ugt2* gene, the encoded retrotransposon proteins could reverse-transcribe the spliced mRNA sequences containing the full-length ORF from this *Ugt* gene. The reverse transcribed cDNA sequences are subsequently integrated into a new site downstream from a promoter [32]. Because the length of the 5′ noncoding sequences depends on the distance between the upstream promoter and the integration site, the initial 5′ noncoding region in the primary transcript could be very large in size [33]. Subsequently, 5′ and 3′ splice sites could be generated by mutations or the insertions of a retronuon (a nuon is any definable nucleic acid sequence) carrying such potential splice sites such as Alu sequences [33]. Splicing between these 5′ and 3′ splice sites could reduce the size of 5′-UTR in the mature mRNA. Recombination or additional rounds of retrotansposition might generate multiple teleost *Ugt5* genes. Similar mechanisms have been proposed to be involved in the expansion of G-protein coupled receptor superfamily with intronless protein coding regions [32], [33], such as the olfactory receptor genes [31], in the vertebrate genomes.

This mechanism is consistent with three observations of the zebrafish *Ugt5* genes. First, we found a polyprotein-like coding sequence located at the immediate upstream of the zebrafish *Ugt5c* cluster (data not shown). The encoded polyprotein contains the domains of protease, reverse transcriptase, and retroviral integrase, which could have retrotransposase activities. Second, the *Ugt5* genes were scattered at many loci in the zebrafish genome. Third, each of the zebrafish *Ugt5* genes contain at least one 5′ noncoding exon (Fig. 2D). Thus, the *Ugt5* loci appear to be hotspots for retrotransposition.

### Conclusions

We have identified and cloned the complete zebrafish *Ugt* repertoire. Zebrafish contains 45 *Ugt* genes that can be divided into three families: *Ugt1*, *Ugt2*, and *Ugt5*. Both *Ugt1* and *Ugt2* are organized into two unlinked clusters: *a* and *b*. Phylogenetic analyses show that both *a* and *b* clusters have orthologs in other teleost species, indicating that they may be resulted from the “fish-specific” WGD event that occurred in the ray-finned fish (*Actinopterygii*) lineage about 350 million years ago [17], [34]. We also cloned five distinct isoforms for members of the zebrafish *Ugt1* and *Ugt2* clusters. The coding region of the zebrafish *Ugt5* genes is encoded by a single large exon. These coding exons are each preceded by small 5′ noncoding exons. Finally, several of the 5′ noncoding exons can be alternatively spliced to generate additional diversity for the *Ugt5b* mRNAs. The diverse 5′-UTR sequences may play an important role in the regulation of the *Ugt5* mRNA stability or their translation efficiency.

Based on the inferred phylogenetic relationships of the vertebrate *Ugt1* genes, we propose that the *Ugt1* clusters in the teleost and tetrapod ancestors contained multiple *Ugt1* paralogous genes. In addition, these ancestral *Ugt1* loci have experienced differential gene loss and duplication in different vertebrate lineages. Moreover, the ancestral vertebrate *Ugt2* gene has undergone lineage-specific duplications. Finally, the *Ugt5* genes appear to be present only in lower vertebrates and have been expanded dramatically in teleosts. The dynamic evolution of the *Ugt* genes in different vertebrate lineages may contribute to the development of chemical defense system for their adaptation to ecological habitats.

## Materials and Methods

### Sequence Annotation

The zebrafish *Ugt* genomic sequences were identified by iterative BLAST search of the zebrafish genome assembly (*Danio rerio* Zv8) using the published vertebrate UGT protein sequences as queries and annotated as previously described [1], [14]. The *Ugt* genomic sequences of fugu, tetraodon, medaka, stickleback, xenopus, lizard, zebra finch, and chicken were identified and annotated similarly (*Takifugu rubripes* v4.0, *Tetraodon nigroviridis* v7, *Oryzias latipes* v1.0, *Gasterosteus aculeatus* v1.0, *Xenopus tropicalis* v4.1, *Anolis carolinensis* v1.0, *Taeniopygia guttata* v3.2.4, and *Gallus gallus* v2.1).

### Cloning and Sequencing of the Zebrafish *Ugt* Genes

Total RNA was prepared from the whole adult zebrafish with the Trizol reagent (Invitrogen, USA), treated with DNase I (Takara, Japan), and reverse-transcribed by using the Superscript III reverse transcriptase (Invitrogen, USA) with *Ugt*-specific primers. The full-length coding sequences were amplified by PCR with Taq DNA polymerase (Takara, Japan), cloned into the pGEM-T vector (Promega, USA), and sequenced in both directions. The primers used for reverse transcription and PCR reactions are listed in Tables S1 and S2. Sequences of the zebrafish *Ugt* cDNAs have been submitted to the GenBank. All of the zebrafish cDNAs and their accession numbers are listed in Table S2.

### Phylogenetic Analyses

The cloned or predicted *Ugt* coding sequences were translated, and the resulting amino acid sequences were aligned by using the ClustalX software (Version 2.0.11) [35]. Phylogenetic trees were reconstructed by using the Neighbor-joining method based on sequence distance matrix, and the trees were displayed using the NJplot program (http://pbil.univ-lyon1.fr/software/njplot.html). The robustness of the tree partitions was evaluated by using the bootstrap analysis with a neighbor-joining search.

## Supporting Information

Table S1 Sequences of primers used. All of the primers used to clone the zebrafish Ugt repertoire are listed. Their sequences are also shown.
(0.04 MB DOC)

Table S2 The GenBank accession numbers for all of the zebrafish Ugt clones. The zebrafish Ugt cDNA clones and their GenBank accession numbers are shown. The primers used in the RT-PCR reactions for each clone are also listed.
(0.04 MB DOC)

Figure S1 An alignment of the vertebrate Ugt1 protein sequences. The zebrafish (z), fugu (f), tetraodon (t), medaka (m), stickleback (s), xenopus (x), lizard (l), zebra finch (zf), and human (h) Ugt1 polypeptides were aligned by using the ClustalX software. The Ugt1 sequence names are indicated on the left and are presented according to the seven groups shown on the Figure 3. The amino acid residues are represented by capitalized single-letters with the degree of conservation indicated above the alignment.
(7.28 MB PDF)

Figure S2 An alignment of the vertebrate Ugt2 protein sequences. The zebrafish (z), fugu (f), medaka (m), stickleback (s), xenopus (x), lizard (l), zebra finch (zf), and human (h) Ugt2 polypeptides were aligned by using the ClustalX. The amino acid residues are represented by capitalized single-letters with the degree of conservation highlighted by different colors. The Ugt2 sequence names are indicated on the left.
(0.21 MB PDF)

Figure S3 An alignment of the vertebrate Ugt5 protein sequences. The zebrafish (z), fugu (f), tetraodon (t), medaka (m), stickleback (s), and xenopus (x) Ugt2 polypeptides were aligned by using the ClustalX. The amino acid residues are represented by capitalized single-letters with the degree of conservation highlighted by differential coloring. The names of the Ugt5 proteins are indicated on the left and presented according to the order of the eight Ugt5 groups (Ugt5a to Ugt5h) shown on the Figure 5.
(0.28 MB PDF)
