# Supplementary material for: Cloning and Comparative Analyses of the Zebrafish Ugt Repertoire Reveal Its Evolutionary Diversity
Source: PLoS One. 2010 Feb 10;5(2):e9144. doi: 10.1371/journal.pone.0009144 (PMC2819257; doi:10.1371/journal.pone.0009144)
Supplement: Table S1 — Sequences of primers used. All of the primers used to clone the zebrafish Ugt repertoire are listed. Their sequences are also shown. (0.04 MB DOC) [file pone.0009144.s001.doc]

**Primer name Primer sequences**

11-b1F GTGCTTGTGTCAGGTCTTCG

11-b1R GACTTCCCAGTTACAGCTTTGT

11-b1R1 TGGACAGCTTTTCTATTAGTTGC

11-b2F CACGAGAACACTGTGACTGGA

11-b2R AGCGCTATCCTGCTGAAGAA

11-b2R1 GCCCTCTCCTGTGGTTTGTA

11-b3F GTTTACAGTATCTGGAGAACACTTC

11-b3R ACACACCAGCAGTGTCCCTT

11-b3R1 GGCTACATCGTTGTCGCATA

11-b4F TTAACAATGCACTTAGGAGTGATAA

11-b4R GCCAACATGATGGAATCAGA

11-b4R1 CCCCTTAAACACTAAGGCCC

11-b5F TAAATATGCATGGAGTGATATTTAA

11-b5R AGGTGCCCTTTAATTGGAGA

11-b5R1 CCATTCTGGAAGAGTCCACAT

11-b6F CAAAGACAGTATATAGGCACAGAGGT

11-b6R AGCTTGTCCAGCCAAATGAT

11-b6R1 CACTAAGGCCTGGTTCAATG

11-b7F GCCAGAATGCCTGTTCCCT

11-b7R TTGCTTCCTCTAAAAATCTTGAA

11-b7R1 CCTCTTGTATGTGCTTATTTGATG

11-bR1 AGGCGTCAAGTTGCTTTCAT

11-bR2 AAAACATTAGAAACCATTGGTATTTA

9-a1F TTGTCTACTGTATAAACATTGGAGGG

9-a1R GCAAACATTTGCATCGACTC

9-a1R1 CGGGCCTTCAGATTAAAACA

9-a2F TGGGTATTTTCTTCTCCTGCC

9-a2R ATCACTTTACTTGGAGGGGG

9-a2R1 TGAAAGCAACGTGCTCAGTC

9-a3F GCCTCTCTGCTCCACAAGTT

9-a3R TCCAACTCTCTTTGATGCCC

9-a3R1 ACAAATGGTTAGGGCATGGA

9-a4F TTAAGCTGGCAAAGGTCACA

9-a4R CAAGAAAGTCCATCACTAGGCA

9-a4R1 TCCCTTTATTGCATAAGTGCTG

9-a5F GATCGTTAAATGGCTTCCGT

9-a5R CAAGCTTTTTGTTCAGTTGCAT

9-a5R1 GCTTTTCATTTCAACAGCAAAT

9-a6F TTGAATAACAGGATTAGATACAGTAGG

9-a6R GTGAAAATGCTCAGTGGGGT

9-a6R1 AAATGGATCCAAACTGTGGC

9-a7F TCTTGCCAATACTACCAGCTCA

9-a7R GATGCCGTTTTTGATTTTGC

9-a7R1 AATTTGCACATGGAAAAGCA

9-aR ACTTGAAGAGGGTCTGGGGT

9-aR2 GTGAGGCTTTATTCAAGTAATTTAT

23-1F GCGCACACACACTCCATCTA

23-1R1 CCTGTACTAAAATGGCGGCT

23-1R2 TGTGTCAGTTGGGGATTTGA

23-1R TCCATCCAAAGTATGTGGTGC

23-2F TCATACTCTGCCATTTGCAATC

23-2R1 GCATTGCTAAGGTCTGTTTTTC

23-2R2 TTTTGAGCAGGCCAGTTAAT

23-2R GGGACAAATATGTAACTTCTGGA

23-VR1 AAATTTCTCCCTCCAGACACA

23-VR CATCATGGACCCTTTAGGCA

23-V1F TCTCATACTCTGTCATTTGCAATC

23-V1R CACTGCTCCTCCACATTTCA

23-V1R1 AACTGGTTTTGTGGTCCAGG

23-V2R TGCTCAGGAACACAGACTGG

23-V2R1 AAAGGAACAAGCCCAAAACC

23-V2F2 GGCTGTACATATTCAAGCAGAA

23-V3F GTCCAAGCAGATCACTGATGA

23-V3R CCAGCCATCAAGCTTCTTTC

23-V3R1 ACTCTTTGTTGCGTTCCCTT

23-V4F GTTCAAGGAGATCACTGATAATTAT

23-V4R AGGGGGCTCTCAACCAAATA

23-V4R1 CGGTTTTGTGATTCGAGGTC

5-1F ACCTCAGTGACTTAAGGAACTATTTA

5-1R TCTATGCTCAGGACGCACAC

5-1R1 AACCAGGTCCAAGCAAAATG

5-1R3 TTTGGTGACAGGTGATGTCAA

5-1R4 GGTAGTCGTTATTTACACATGAACTC

5-2F GAAGTATATTGAGGACAAAGTCATTAC

5-2R CGGCTTTTAATTGCAGTATCTCA

5-2R1 GCCACAGCGGATTGAACTAT

5-2R2 GCCACTTCTGAGACCAAATGA

5-2R3 TCAGGAAGGGTAGTGGAAAA

5-a1F TCCAGTTTCCAGAATCATTCAC

5-a1R1 CAGAAACGTTGACTGGCTTG

5-a1R2 CACGTGAGCACAGGAAGAAC

5-a2F TTGTTTTCGAGTTCTTCTGCC

5-a2R1 TGGCTGAAACAACACTTGAGA

5-a2R2 TTCAAAGATGATTCCTTGAGTTT

5-a3F GTTCAGATCACTCTGCGCTCAT

5-a3R1 CTCCTCTAAATGGCAAAGCG

5-a3R2 AAGAACGGACCCCAGCATAG

5-a4F CAAGAGCAGGAACGCTTCAC

5-a4R1 ACCTTGTTTTAGCGAAGATGAC

5-a4R2 CGTCTAGTTTTGGCCTGCTC

5-aR TGATCGTCTGAAAGTTCCCC

5-aR2 TACTGCATCTCTGTGGGGAA

5-aF ATTTCGAGTATCCGCGTCC

10-1F CATGCATGAACAATTAAGGT

10-1R TGGACCATGTTTCCACATTTT

10-2F CCCTCGTTTTCCTTTGGACT

10-2R CAGCATTAACAGACTCATTCAACC

7-1F1 CATGGATGGACAAACTGACG

7-1R CTGCTGTTTGAGCCACAGAA

8-1F CCACACTGCAAAACTTCAGG

8-1R TGACAATGATCAAGGATGCG

8-1R2 TGGAACATCATGACGGTGAG

25-F CTGAGGGGAAATTCAAGCAG

25-1R TTTCATTTGAACTGATATTAAGCCA

25-2R TTGGCTGATTGTTGTGGAAC

25-3R GTATTGACAGGACTGCATTTGTTCT

25-4R AGTGGAGCTAAATTCAAATTAACTG

25-5R TAAATGTGCACCCTGATCCA

25-5F1 GTGGTTGTATTTTCAGGCGG

25-5R1 ATTGCTGGGAAGGCATACAG

25-3R1 GGAAGCAGCTACTGATGGCT

25-1R1 TCGGCTGATGCTAAACCTTC

18-1F1 GACGTCTATGCCAAAATAACTGT

18-1R CCATTAAGTGGCACTGGGAT

18-2F GACAGAAGCATATGCCGGTC

18-2R CTTTATAGTTCAAATGGTTCAATCAG

18-3F TGCGAGTGAATCCTGTTGAG

18-3R GAACCTACCAATCGCACCAC

18-4F TTCACGAGCTCGCTGCTAC

18-4R TTGGACTAAAGGAACTCTCTTCAC

18-1R1 TGCAGTTTCACAGGAAAGGA

18-2R1 GTCAAACACTGAACATTGTCAAA

18-3R1 TGCAGAGCAGTTAAGTGCAAG

1-F1 AACTCAAACACAACAGGGGG

1-F2 TTGTTTGGACGTTGTCGAAG

1-1R GCCAGTTCTTCTAAATACTCATTAT

1-2R AATGCCTGACCTGAATTTGC

1-3-1R TGACAGGTTCTTCAGTTGCC

1-3-3F TGGTAATGACTTGCATCCCA

1-4R TGTATGCCCAGCCTAAATCTTT

1-1R2 CACACACGTAGGAGTAAACCCTT

1-1R3 TGCAGACGGTCTCTTTCCTT

1-3R1 CACGCTCTATTTCAATCATCTTC

1-4R2 GCAGATGTTTTTGTCTGCTCC

1-5PF GGTGACTTACACTCCATGCAA

1-5PR TCCTCGACCGTCACAACATA

1-5PR1 CCAAAATATTTCCCCAAGCA
