# Supplementary material for: Cloning and Comparative Analyses of the Zebrafish Ugt Repertoire Reveal Its Evolutionary Diversity
Source: PLoS One. 2010 Feb 10;5(2):e9144. doi: 10.1371/journal.pone.0009144 (PMC2819257; doi:10.1371/journal.pone.0009144)
Supplement: Table S2 — The GenBank accession numbers for all of the zebrafish Ugt clones. The zebrafish Ugt cDNA clones and their GenBank accession numbers are shown. The primers used in the RT-PCR reactions for each clone are also listed. (0.04 MB DOC) [file pone.0009144.s002.doc]

**Clone Forward Reverse RT Accession**

**name primer primer primer number**

Ugt1a1_v1 9-a1F 9-aR2 9-aR2 GU299096

Ugt1a2_v1 9-a2F 9-aR2 9-aR2 GU299098

Ugt1a3_v1 9-a3F 9-aR2 9-aR2 GU299100

Ugt1a4_v1 9-a4F 9-aR2 9-aR2 GU299102

Ugt1a5_v1 9-a5F 9-aR 9-aR2 GU299104

Ugt1a6_v1 9-a6F 9-aR 9-aR2 GU299106

Ugt1a7_v1 9-a7F 9-aR 9-aR2 GU299108

Ugt1b1_v1 11-b1F 11-bR1 11-bR1 GU299110

Ugt1b2_v1 11-b2F 11-bR1 11-bR2 GU299111

Ugt1b3_v1 11-b3F 11-bR1 11-bR2 GU299113

Ugt1b4_v1 11-b4F 11-bR1 11-bR1 GU299115

Ugt1b5_v1 11-b5F 11-bR2 11-bR2 GU299117

Ugt1b7_v1 11-b7F 11-bR1 11-bR1 GU299120

Ugt2a1_v1 5-a1F 5-aR2 5-aR GU299122

Ugt2a2_v1 5-a2F 5-aR2 5-aR GU299169

Ugt2a3_v1 5-a3F 5-aR2 5-aR GU299127

Ugt2a4_v1 5-a4F 5-aR2 5-aR GU299129

Ugt2a5_v1 5-2F 5-2R 5-2R1 GU299131

Ugt2a6_v1 5-1F 5-1R 5-1R1 GU299133

Ugt2b1_v1 23-V2F2 23-VR 23-VR1 GU299135

Ugt2b3_v1 23-V1F 23-VR 23-VR GU299139

Ugt2b5_v1 23-V3F 23-VR 23-VR1 GU299142

Ugt2b6_v1 23-1F 23-1R 23-1R GU299145

Ugt1a1_SF 9-a1F 9-a1R1 9-a1R GU299095

Ugt1a2_SF 9-a2F 9-a2R1 9-a2R GU299097

Ugt1a3_SF 9-a3F 9-a3R1 9-a3R GU299099

Ugt1a4_SF 9-a4F 9-a4R1 9-a4R GU299101

Ugt1a5_SF 9-a5F 9-a5R1 9-a5R GU299103

Ugt1a6_SF 9-a6F 9-a6R1 9-a6R GU299105

Ugt1a7_SF 9-a7F 9-a7R1 9-a7R GU299107

Ugt1b1_SF 11-b1F 11-b1R1 11-b1R GU299109

Ugt1b2_SF 11-b2F 11-b2R1 11-b2R GU299170

Ugt1b3_SF 11-b3F 11-b3R1 11-b3R GU299112

Ugt1b4_SF 11-b4F 11-b4R1 11-b4R GU299114

Ugt1b5_SF 11-b5F 11-b5R1 11-b5R GU299116

Ugt1b6p_SF 11-b6F 11-b6R1 11-b6R GU299118

Ugt1b7_SF 11-b7F 11-b7R1 11-b7R GU299119

Ugt2a1_SF 5-a1F 5-a1R1 5-a1R2 GU299121

Ugt2a2_SF 5-a2F 5-a2R1 5-a2R2 GU299125

Ugt2a3_SF 5-a3F 5-a3R1 5-a3R2 GU299126

Ugt2a4_SF 5-a4F 5-a4R1 5-a4R2 GU299128

Ugt2a5_SF 5-2F 5-2R3 5-2R2 GU299130

Ugt2a6_SF 5-1F 5-1R3 5-1R4 GU299132

Ugt2b1_SF 23-V2F2 23-V2R1 23-V2R GU299134

Ugt2b2p_SF 23-V3F 23-V3R1 23-V3R GU299137

Ugt2b3_SF 23-V1F 23-V1R1 23-V1R GU299138

Ugt2b4p_SF 23-V4F 23-V4R1 23-V4R GU299140

Ugt2b5_SF 23-2F 23-2R2 23-2R1 GU299141

Ugt2b6_SF 23-1F 23-1R2 23-1R1 GU299144

Ugt2a1_v2 5-a1F 5-aR2 5-aR GU299124

Ugt2a1_v3 5-a1F 5-aR2 5-aR GU299123

Ugt2b1_v4 23-V2F2 23-VR 23-VR1 GU299136

Ugt2b5_v5 23-V3F 23-VR 23-VR1 GU299143

Ugt5a1 25-F 25-1R 25-1R1 GU299146

Ugt5a2 25-F 25-2R 25-2R GU299147

Ugt5a3 25-F 25-3R 25-3R1 GU299148

Ugt5a4 25-F 25-4R 25-4R GU299149

Ugt5a5 25-5F1 25-5R1 25-5R GU299150

Ugt5b1_v1 1-F1 1-1R 1-1R2 GU299151

Ugt5b1_v2* 1-F1 1-1R 1-1R2 GU299152

Ugt5b2_v1 1-F2 1-2R 1-2R GU299153

Ugt5b3_v1* 1-F1 1-3R1 1-3-1R GU299154

Ugt5b3_v2* 1-F1 1-3R1 1-3-1R GU299155

Ugt5b3_v3 1-3-3F 1-3-1R 1-3-1R GU299156

Ugt5b4_v1 1-F2 1-4R2 1-4R GU299157

Ugt5b4_v2 1-F2 1-4R2 1-4R GU299158

Ugt5b4_v3 1-3-3F 1-4R2 1-4R GU299159

Ugt5b5p 1-5PF 1-5PR1 1-5PR GU299160

Ugt5c1 18-1F1 18-1R1 18-1R GU299161

Ugt5c2 18-2F 18-2R1 18-2R GU299162

Ugt5c3 18-3F 18-3R1 18-3R GU299163

Ugt5d1 10-1F 10-1R 10-1R GU299164

Ugt5e1 8-1F 8-1R2 8-1R GU299165

Ugt5f1 18-4F 18-4R 18-4R GU299166

Ugt5g1 7-1F1 7-1R 7-1R GU299167

Ugt5g2 10-2F 10-2R 10-2R GU299168

*cDNAs that do not contain full-length coding sequences.

SF represents the short form.
