# Supplementary material for: Cloning and Comparative Analyses of the Zebrafish Ugt Repertoire Reveal Its Evolutionary Diversity
Source: PLoS One. 2010 Feb 10;5(2):e9144. doi: 10.1371/journal.pone.0009144 (PMC2819257; doi:10.1371/journal.pone.0009144)
Supplement: Figure S1 — An alignment of the vertebrate Ugt1 protein sequences. The zebrafish (z), fugu (f), tetraodon (t), medaka (m), stickleback (s), xenopus (x), lizard (l), zebra finch (zf), and human (h) Ugt1 polypeptides were aligned by using the ClustalX software. The Ugt1 sequence names are indicated on the left and are presented according to the seven groups shown on the Figure 3. The amino acid residues are represented by capitalized single-letters with the degree of conservation indicated above the alignment. (7.28 MB PDF) [file pone.0009144.s003.pdf]

```
                                                                 :* :*   . *        :  .*:
x1a1     -------------------------MRLLCTLEYSVYRVFALLFLGRVQLIEAEKLLAIPLDGSHWLSMRILVESLGQRGNQVVVLAPHSNIHIHPS--E   73
x1a3     -------------------------MELLASSNAAVG---LLFCLGALSVTEGGKLLVVPMDGSHWLSMSSVVERLAQNGHEIIVVKPESSIVKFDS--N   70
x1a4     -------------------------MELLASSNAAVW---LLFCLGALSVSEGGKLLVVPMDGSHWLSMSSVVERLAQNGHEIIVAKPENSIVKFDS--N   70
x1a5     -------------------------MGATGSSPSCCFFR-RFFILLALGCADGGRLLVVPQDGSHWLSMRVLVDRLAQNGHHIVMAIPEVNMFFNDL--D   72
x1a6     -------------------------MGATGSSPSCCFFR-RFFILLALGCADGGRLLVVPQDGSHWLSMRVLVDRLAQRGHYIVVAIPEVNMLFKDL--D   72
x1a2     -------------------------MPPLFLLLVGFG---VVCLLELPGLSEGGKVLVIPQDTNVWLDMHSMVNKLKKNGHEIVVVIPKNNDISHLS--E   70
l1a1     -----------------------------MLQSVCQIPSWILLTFFSWSLSDGGKVLVIPADGSHWLSLKPVMENLQQRGHELVVVAPEANLWIKAS--E   69
l1a2     --------------------------MA-CLKSRSHIAVGITCLLSFCHLAESGKLLVVPQDGSHWLSMKMALEELAQRGHEVVVLMPENTFLMQTS--E   71
zf1a1    -------------------------MVF-PKPGFLFLASWAVFFLLLWTSGEGGKLLVIPIDGSHWLSMHPVVEKLRDKGHEIVVVAPEINLRIHSS--P   72
zf1a2    -------------------------MTV-MWRHHVHAG--LVFFLAFWSLAHGGKLLVVPQDGSHWLSMRMVLEKLWEKGHEIVAVVPDAALLLKSS--Q   70
l1a3     -------------------------MAL-PLP-LCSLVLWAFSLLISSPSADAGKLLVLPMDGSHWLSMSKVLKKLSLKGHEIVVLVPETSLLLKD---L   70
l1a4     -------------------------------MAPVFYSNVILLLLASLNLASGGKLLVIPMDGSHWLSMKPVLKQLRQNGHEMVIIAPEVSIHIKPEE-D   68
l1a5     -------------------------------MAPVFYSNVILLLLASLNLASGGKLLVIPMDGSHWLSMKPMLKQLRQNGHEMVIVAPEVNMHIKPAE-D   68
zf1a3    -------------------------MAL-ELRASPPVV---LLLLSLLGLAAAGKLLVVPVDGSHWLSMQELLVLLQQKGHEVVVVAPEVSLHIKPS--K   69
zf1a7    -------------------------MAL-GLRASPPVG---LLLLSLLGLAAAGKLLVLPADGSHWLSMRELLDLLQQKGHEVVVVAPEVTLHIKPS--K   69
zf1a8    -------------------------MAL-GLRASPPVV---LLLLSLLGLAAAGKLLLVPVDGSHWLSMREVVDLLRQRGHEVVVVAPEVTLHIKPS--K   69
h1a1     -------------------------MAVESQGGRP-LVLGLLLCVLGPVVSHAGKILLIPVDGSHWLSMLGAIQQLQQRGHEIVVLAPDASLYIRDG--A   72
h1a3     -------------------------MATGLQVPLPWLATGLLLLLSVQPWAESGKVLVVPIDGSHWLSMREVLRELHARGHQAVVLTPEVNMHIKEE--N   73
h1a4     -------------------------MARGLQVPLPRLATGLLLLLSVQPWAESGKVLVVPTDGSPWLSMREALRELHARGHQAVVLTPEVNMHIKEE--K   73
h1a5     -------------------------MATGLQVPLPQLATGLLLLLSVQPWAESGKVLVVPTDGSHWLSMREALRDLHARGHQVVVLTLEVNMYIKEE--N   73
zf1a10   -------------------------MAL-FYQGAWILL---LLTLSL-ILAEGGKILVVPQDGSHWLSMRPVVEKLQQKGHEVVVVVPSTSLYMKSKEPQ   70
h1a6     -------------------------MAC-LLRSFQRISAGVFFLALWG-MVVGDKLLVVPQDGSHWLSMKDIVEVLSDRGHEIVVVVPEVNLLLKES--K   71
l1a6     -------------------------MDH-HFTCPRGFPICFLFLFAIICFGEGKRLLVIPVDGSHWLSMRAVLEKLTERGHEVVVLVPEVNLLIKES--K   72
l1a7     -------------------------MAF-ACHRPTSMPVFLLALLAIFASVDSEKLLVIPQDGSHWLSMRAVVEKLSEKGHEIVVVVPEVNLLLKES--R   72
zf1a11   -------------------------MAL-RLCCAWISI---LLLLP--SLSDGGKLLVVPMVGSHWLSMQEVVEKLSERGHEVVVLVPKVSWQMETT--Q   67
h1a9     -------------------------MAC-TGWTSP-LPLCVCLLLTCG-FAEAGKLLVVPMDGSHWFTMRSVVEKLILRGHEVVVVMPEVSWQLGRS--L   70
h1a7     -------------------------MAR-AGWTGL-LPLYVCLLLTCG-FAKAGKLLVVPMDGSHWFTMQSVVEKLILRGHEVVVVMPEVSWQLGRS--L   70
h1a10    -------------------------MAR-AGWTSP-VPLCVCLLLTCG-FAEAGKLLVVPMDGSHWFTMQSVVEKLILRGHEVVVVMPEVSWQLERS--L   70
h1a8     -------------------------MAR-TGWTSP-IPLCVSLLLTCG-FAEAGKLLVVPMDGSHWFTMQSVVEKLILRGHEVVVVMPEVSWQLGKS--L   70
z1a1     -------------------------MRTLPVPAQGLLTLLCLFSFESVQA---GKVLVLPVDGSHWLSMKILVEELSNRGHEMVVLVPETSILIKKS--G   70
z1a3     ------------------------MKRTLFVPALGLFAFLCLFSSESVQA---GKVLVMPVDGSHWLSMKILVEEMSSRGHEMVVLVPETSILIGKS--G   71
z1b1     ----------------------MLKKGWLWGLGSGLLLLWLSAGLRPVQG---GRVLVMPVEGSHWLSMKVLATELARRGHDVLVLVPEKNILIQSS--E   73
m1a1     ------------------------MGVRGWFPTAGLLAWLCCLSLGPVQG---GKVLVMPVDGSHWLSMKILVKELSRRGHEVTVLVPESSLLIKGS--K   71
s1a1     ------------------------MSGEVWFPALGLVAWLCCLSLGPAQG---GKVLVVPVDGSHWLSMKILVKELSHRGHEAVVLVPETSLLIHGS--K   71
f1a1     ------------------------MRGSGWFPTLGLLAWFCCFGPRPVEA---GKVLVLPVDGSHWLSMKILVKELVQRGHDVLVLVPETSLLIKSS--E   71
t1a1     ------------------------MRGSVGILILGLLAWISCFGPRPVQA---GKVLVLPVDGSPWLSMKILMKKLIQRGHDVLVLVPETSLLIKSS--E   71
z1a2     --------------------------------MACLWLLGSLLLLGSAEA---GKLLVIPTDGSHWLGLKPIVEELGRRGNQVVVVIPEASLSMGPS--E   63
z1a4     --------------------------------MACSWLLVGLFLFGSAEA---GKLLVIPSDGSHWLGMKPVVEELGRRGNQVVVVIPEASLSMGPS--E   63
z1a5     ---------------------------------MASVLLFCLFCLASAKA---GKILVVPSDGSHWTGIKPLVEELGRRGNQVVAVIPEASLSMGPS--Q   62
z1a6     --------------------------------MAAFVLLLYLFCLATAEA---GKILVVPSDGSHWTGMKPLVEELGRRGNQVVVVIPEASLSMGPS--Q   63
z1a7     -------------------------MNDRIVRMAASALVLCLFCLVSAEA---GNLLVIPALGSHWTGTRPLVEELGRRGNRVVVVFPEENVNMVPA--K   70
z1b2     -----------------------MRLGVIFILVVGVVCSAAVDQKPSRKSSWTGKLLVVPMDGSHWTGVKAVAEEMGRRGHTVIVVIPEISVLLGPG--Q   75
z1b3     -----------------------MHLGVIFCLVVGVVCSAAVDQKLSRKSSWTGKLLVVPMDGSHWTGVKAVAEEMGRRGHTVIVVIPEISMRLGPG--K   75
z1b4     -----------------------MHLGVIICLVVGVVCSAVVDQKLSRNSSWTGKLLVVPMDGSHWTGVKAVAEEMGRRGHTVIVVIPEISMRLGPG--K   75
z1b5     -----------------------MH-GVIFNLVVGVVCSAAMNQKPSRNSSWTGKLLVVPMDGSHWTGVKAVAEEMGRRGHTVIVVIPEISMRLGPG--K   74
z1b7     -----------------------MGLVAFIWCIVLTFASAGEAVK-SKEASWTGSLLVVPMDGSHWTGVKALAEEMGRRGHKVTVVIPEVSVLLGPG--K   74
f1b1     MRSAALLVIMLLSMQVKGAVDKTAGPVGGGNQRPEETKGTTNTAND-SDPPFLGNLLVVPMDGSHWVDLKALAQEMGRRGHRVTVVIPEVSMRMGPG--K   97
f1b2     MRSAALLVIMLLSMQVKGAVDKTAGPVGGGNQRPEETKGTTNTAND-SDPPFLGNLLVIPMDGSHWVDLKALAQEMGRRGHRVTVVIPEVSIRMGPG--K   97
t1b1     MRSATLFTVLLLSLQVKGGVR-TENSAGKGNQRPEE---TETPAND-SDAPFLGNLLVVPMDGSHWVDLKALAQELGRRGHRVTVVIPEFSIRMGPG--K   93
s1b1     ------MQVNGAAGEAGGSVAGGPGPETKLKAAEAEAANTIPASDT-APAGFLGNLLVVPMDGSHWVGIKAIAEEMGRRGHKVTVVIPEISVRMGPG--K   91
m1b1     --MKLLFVLLLLSMSVG--VRGAASVAD--DPRPEKTEKTKVSAEPGSSAAFSGKLLVVPMDGSHWVGIKAIAQELGRRGHRVTVVIPEVSMRMGPG--K   92
m1b2     --MRLLFVLLLLSMSVG--VRGVASVAD--EPRPKKTEKTKVSAEA--SAAFSGKLLVVPMDGSHWVGIKAMAQELGRRGHRVTVVIPEVSMRMGPG--K   90
```

```
                  .                                                    :: .      .    *:  :  .    . : .  : :  :
x1a1        NYILRTYPVPYSKELLKEQMVKSANDVFTK--RPLLDKITNMYQRITNVTGIVFSACQHLLHSKEIMQELQESAFDAALIDPFFPCGMIVAEYLHLPSVY  171
x1a3        LYTLRNFSTPYSNQDIHERLQQLVGSQFTA--SSLIEMVLNSYNSIMGVQHMINSMCESLLHNKQFIRNLQEEKFDAVLNDPMFFCGVILAEHLGIPSVH  168
x1a4        LYTLRNFSTPYSNQDIHEHLQQFVGSQFSG--SSLIEVVLTARNSLMDVQHMMINMCKSLLQNKQLIQNLQEEKFDAVLNDPMFFCGVILAEHLGIPSVH  168
x1a5        NYKVKTFSSPYSKDVLESRVRHMNLEIFAD--RILTDKIKFLYESVSNGTMSVVSACQHFLQNSTLIRELEKEHFDAMLTDSVFPCGEIVAEHLSIPSVS  170
x1a6        NYKVKTFSSPYSKDVLENRMRRMNQEIFVD--RILTEKITFWYESMSNATQWVVSACQHFLQNSTLIRELEKEHFDAMLTDSVFPCGEIVAEHLSIPSVS  170
x1a2        DFIVKTYTVPALDSGFQIYLEPTEP-LFQM--SSFLEAISTEYRRWKYTTSVLCNTCQHLLDDLELIRYLERSLFDAAIMDPWFPCGPVLAEHLSLKSIY  167
l1a1        AYTMKTFAVSHTTEYLKAEFQKLGHRIFAH--QPFLTKMTESFAKVRNITTLFFDNCKQLLYNKELITYLEENQFDVVLMDPVSPCGQILAEHLSIPSVY  167
l1a2        HFTVRTHSVPYTQEELDAHYRSLGTAIFDD--SPFLEKIFLMYINTTRITAFYASACRDLLYDKELITFLQQSKFDAMFSDPILPCGPILAEYLSLPTVY  169
zf1a1       IYTLKTYPVSYTKEFVEAEFKEMGHRSFAP--HPFLEKLS----KIANFTSMFLDSCKRLLSDKELIQYLEGSKFDAIFMDPFFPCGQIVAEHLSLPSVY  166
zf1a2       SFTIKTYLVPYTQEYVDQYYQKLGENSFET--LTLSFLLS----NITELTNMFTSACRHLLSDKELMKYLQDSKFDAIMMDPVLPCGPILAEYLSLPSVY  164
l1a3        SYTTKIYPVPHTQKELEEVYQKFGHSLFSD--EPFVKRVIQIHKNLREFINMMYSSCHSLLHNEDLMNYVNVSRFDAVLTATTVPCGSIVSDFFQIPVVN  168
l1a4        FYTLKTYPVPFTSEEMNENIQSFSDHVFED--VPFLVMIAKTFELMKKSSAMFLGSCTHLLYNEELKMYFRENKFDAVFSDPFWPCGQIVAEYLEIPSVF  166
l1a5        LYSLKTYPVPFTYEEMDENIQGISEQAFDD--VPFLLMIANTIEHMKKTSVFFLGSCTHLLYNKELRMYFRENKFDAILSDPFWPCGQIVAEYFDIPSVF  166
zf1a3       TIVMKTYPVPFTQEEMDKVFKGSVMDLFKG--GPFLERVIRQYQQAKKTSALFLATCTHLIYNKELLSYLEESRFDAILTDPILPCGAILAKYLSLPSVY  167
zf1a7       NFVMKMYSVPFTQEELEKAFQAFFHGSFEE--GWFFKRFFKVYRGMKTLTDCWVTSCEQLLQSKELIRYLEESKFDAILTDPVATCGLILAEHLSLPSMY  167
zf1a8       NFVMKMYSVPYTKEDLKKEFQAFFHFSFEQ--GPFLERLVKAYQGIKRITDFGVTSCEQLLQNKELVTYLEDSKFDAILTDPVQLCGAILAKHLSLPAVY  167
h1a1        FYTLKTYPVPFQREDVKESFVSLGHNVFEN--DSFLQRVIKTYKKIKKDSAMLLSGCSHLLHNKELMASLAESSFDVMLTDPFLPCSPIVAQYLSLPTVF  170
h1a3        FFTLTTYAISWTQDEFDRHVLGHTQLYFET--EHFLKKFFRSMAMLNNMSLVYHRSCVELLHNEALIRHLNATSFDVVLTDPVNLCAAVLAKYLSIPTVF  171
h1a4        FFTLTAYAVPWTQKEFDRVTLGYTQGFFET--EHLLKRYSRSMAIMNNVSLALHRCCVELLHNEALIRHLNATSFDVVLTDPVNLCGAVLAKYLSIPAVF  171
h1a5        FFTLTTYAISWTQDEFDRLLLGHTQSFFET--EHLLMKFSRRMAIMNNMSLIIHRSCVELLHNEALIRHLHATSFDVVLTDPFHLCAAVLAKYLSIPAVF  171
zf1a10      NYTVKVYPVPYTDEYLGEILKALVNAHFME--QSVLNVVLTLYQVMIEIPSVFFTNCKSLLQNEELMQHLKESKFDVVFTDPILMCGPLVAEYLSVPAVY  168
h1a6        YYTRKIYPVPYDQEELKNRYQSFGNNHFAE--RSFLTAPQTEYRNNMIVIGLYFINCQSLLQDRDTLNFFKESKFDALFTDPALPCGVILAEYLGLPSVY  169
l1a6        YYTMKKYAVPYMQDDLLQRFRLLGSNPFEE--VSFPAMVIGAYRTLMQLIELNSVSCDNLLKNREVMRYLAENRFDALFTDPAVPCGVILAEYLSVPSLY  170
l1a7        HYMLKTYSVPYEQKDLDAGFKKFSTMLFRK--VSFPYSILQEYRNNMYVINLFFDNCNSLLKDREVIKFLEQGHFDALFTDPALPCGVILAEYLSLPSVY  170
zf1a11      AYKVVTYPVRQTLGELDNAFQEYVAVHLMQ--KPFPLNALAMYKASVHVFSTFFGQCKDLFHSQETLRFLNQSSFDAILTDPAFMCGAILAHHLSLPFVF  165
h1a9        NCTVKTYSTSYTLEDLDREFKAFAHAQWKAQVRSIYSLLMGSYND---IFDLFFSNCRSLFKDKKLVEYLKESSFDAVFLDPFDNCGLIVAKYFSLPSVV  167
h1a7        NCTVKTYSTSYTLEDQDREFMVFADARWTAPLRSAFSLLTSSSNG---IFDLFFSNCRSLFNDRKLVEYLKESCFDAVFLDPFDACGLIVAKYFSLPSVV  167
h1a10       NCTVKTYSTSYTLEDQNREFMVFAHAQWKAQAQSIFSLLMSSSSG---FLDLFFSHCRSLFNDRKLVEYLKESSFDAVFLDPFDTCGLIVAKYFSLPSVV  167
h1a8        NCTVKTYSTSYTLEDLDREFMDFADAQWKAQVRSLFSLFLSSSNG---FFNLFFSHCRSLFNDRKLVEYLKESSFDAVFLDPFDACGLIVAKYFSLPSVV  167
z1a1        KYSTKTYPVSFTHDDLAENLKEIQNSALEK--APKLTDIVVNFRNLLQFLTMQSKTCEGLLYNEPLMKSLREMGFDAMLTDPFLPCGTIIADSFSIPAVY  168
z1a3        NFTTKSFRVPYSFDELNAHVDHIRKTAIEK--APRFIDIVGALGNLIQFTNMQVKACEGLLYDEPLMKSLRDMKFDALLTDPFLPCGSVIADYFSMPAVY  169
z1b1        LFRTETFPVKISKEQLSKSLKGFQQGVFTRS-PALMD-VFVQLERLLNFTGSQVEGCESLLYNEPLMRKLKEQNFELMLTDPFLPCGPIIATALGVPAVY  171
m1a1        DYKTEVHKVPFTQEELNGKFDKLRESVFVK--TPEFTDMFVNVQRLVEFTTLQVKGCESLLDSEDLMERLRGEGFDAMLTDPFLPCGSILAKMFSIPAVY  169
s1a1        SYRTEMYQVPFTKAELDGAFNQLKDGVFLK--PPTITDLFINIQRLVNFTSMQVGACESLLYNQPLMSKLKSEGFDLVLTDPFLPCGSILSHVFSIPAVY  169
f1a1        NYRTEIYQVPYSKEDLDGNFNELKDGLFDK--PPTMADIFINVERLVTFTTMQVSGCESLLRNQPLMTRLQEQGFEVVLTDPFLPCGSILSHLFNVPAVY  169
t1a1        NYRTEIYQVPYSKEDLDENFKLLKDGVFLK--PPSMADVFVNVERLMNYTTMQVTGCESLLRNQPLMTRLREQGFEVVLTEPFLPCGSILSHLFNIPAVY  169
z1a2        KTTTLTYPVNYTKVELEAVLASELNALLSIDISTDLAKFQSFFFTLNVLQTFILRNAEGLLFNKDLMKKLQDYNFDAILTDPFEPVGAIAGEYLSIPAIY  163
z1a4        KTTTLTYPVNYTKAELHMVLEGNLTEILSTDFSTELSKFFVFFLQLKVLQNFIVRNAEGLLFNEDLMKKLQDCKFDAILTDPFEPVGVIASEYLSIPAIY  163
z1a5        HTTTLTYPVPYTKAQIQDHISASVTNLISTHVSTDLARFQSFINAMDLLSNLITCNVEGLLLNKDLMKKLQDYNFDVILTDPFELVGVIAAEYLSVPTIY  162
z1a6        HTTTLSYPVPYTKAQIKEIVKAGVTTLISTYVSTDLARFQSFINRMNSLSNIIIRSAEGFLSNKDLIKKLQDYNFDVILTDPFETVGVIAAEYLSVPAIY  163
z1a7        HTTTLTYPVPYTKAQIQKGTDAAISKLFSADVSSDVGRFQNFFTTMDMLKVIISRNAEGLLLNKDLLKKLQDYNFDAILTDPFETVGVIASEYLSIPAIY  170
z1b2        HYITKMFSVKYDQKSLNKVLNERVLEVTNPG-NSFLSTVIRIVSNLRRMFNTMAATSESLFQDKELIESLRNENFDAVLTDPVLPMGAILAYNLSVPAVY  174
z1b3        HYITKKFPVKYDQKLFNEVLTEHVHEVTNPG-HSRLKTVTSTMANLLKMFNMMASTSESLFQDKELIKFLRDENFDAILTDPALPMGAVLAYNFSVPAVY  174
z1b4        HYITKKFPVQYKQDLINQIMVEHVNEVAASE-QSLLKTVTSIMYRFGKIIDHFISTSESLFQEQELIEFLRDQNFDAVLTNPAMPMGAILAYNLSVPAVY  174
z1b5        HYITKKFPVKYKPDVLNQMIAKLVNTLSAPE-QSLLKRVTSNIYNFGKVVDYFISTTESLFQEQELIEFLRDQNFDAVLTDPAMPTGAILAYNLSVPAVY  173
z1b7        HYVTRTFPVLYGKQQLDELQARNAQVMESKQ-LPLMEKISTRFSNMRKFVNLQRATAESLLLNQELVDFLRKQNFDAVLTSPAVPTGAILAYNLSLPAVY  173
f1b1        YYDTVTFPVPYDKDLVA-SLMGDNKGILEKSTVGFTEKIQNRISQFQTFASFLHTTAESLLFNASIISQLEQQNFDAVLTDPMVPTGALIARKLGLPIVN  196
f1b2        YYDTVTFPVPYDKAVID-SIMVANKDVIKKSTVSFMEKIQKQFTQFQKIKGFLHSTAESLLFNASIISQLEQQNFDAVLTDPMVPTGALIARKLGLPIVN  196
t1b1        YYDTVTYPVPYTQADID-SIIAANKDVLEKSTLSFTDKISKRFNQFRKIASFLHSTAESLLFNASVISQLEQQKFDAMLTDPMVPTGSLIARKLGIPVVN  192
s1b1        HYETVTFPVPYDKAHLD-FVMAVHKDVLRKSTQSFIQNIKAKYSQIMRITGFIHTTAESLLFNDSLISHLAQQNFDAVLTDPMIPSGSLIARKLGLPTIN  190
m1b1        HYDTLTHPVPYGQADID-VMVSMHGSMVR-SDGPFMERMKTKFHHIKRVIDFIHVTAESLLFNDSFISYLEQQEFDAVLTDPVIPSGSLIARKLGLPTIN  190
m1b2        HYDTLTHPVPYGQSEVD-AILSTQKDFLE-TAQPFMERIETKYHNFVRMVNFIHVTAESLLFNDTLISYLAKQDFDAVLTDPMLPTGSILARKLGLPTIN  188
```

```
                                             * : :*  . .                  :    :        :     .*: : ::     :* :*
x1a1        LLYGLPCGIDRLATQCPSPLSYVPRHFTGNTDHMSFPQRLKNALLWPVDTFLCNLLYSPYAQLASEFLHREMTILQLYSQTSVWLMKTDFVFDYPRPLMP   271
x1a3        FLRGIPCMYDYISAQCDSPLSYVPRHFTQNSDKMNFLQRVGNTLLSLAEPILCGAAFRGWEHLASEFLQREVTAIQLLSRTSVWLMRVDFVFDHPRPVMP   268
x1a4        FLREVPCMYDYISAQCDSPLSYVPRHFTQNSDKMNFLQRVGNTLLRLAEPIFCGAAFRGWEHLASEFLQREVTAIQLLSRTSVWLMRVDFVFDYPRPVMP   268
x1a5        FTRGAAFGMDQIGSQTPSPPSYVPRMFTVYTDNMSFSQRVKNFLIHSLEHIVCHIFYYPYAQMASDFLKKEVTAMSLFSRTSIWLMRYDFVFEFPRPIMP   270
x1a6        FMRGTIFGMDQIASQTPSPPSYVPRMFTVYTDNMSFSQRVKNFLIHSLEHIVCHIFYYPYAQMASDFLKKEVTAMSLFSRTSIWLMRYDFVFEFPRPIMP   270
x1a2        VMPGFSCGWEYTATQCP-------KSPYQHYGQMDFTQRMKSLLAESLEYIACQEIKAEYTRLASQFLHKHINILQVFSQAELWLFRYDFVVESAKPVMP   260
l1a1        YLRGIPCGLDFEASQCPNPHSYVPKFFTRNTDRMVFNQRVKNFLVGSLEFIICYLLYSPYEVLSKEFLQRDMTITELYSQASIWLLRYDFVFEYPRPVMP   267
l1a2        FLRGLPCGLDYKAAQCPVPLSYVPWGFTSFSDHMSFIERVKNLITSPLQYLLCRLFFANHEELASEFLQRTTTISELFSKTSIWLMRYDFVFEYPRPVMP   269
zf1a1       FLRGLPCSLEFQAMMCPSPPSYVPRFFTRSTDRMSFWQRLGNFLATMGSSLACSVLYSPYDPLIQEFLQREATVPELFSHAAVWLMKYDFVFEYPRPLMP   266
zf1a2       FMRGLPCTLDYKAAQSPSPVSYVPKTLSSLSDHMTFPERVKNFLIGLSEPLLCHLFYLKYESLASEFLQRDVTIQELFSQASVWLMRYDFVFEYPRPIMP   264
l1a3        FLRGIACNLDLKAAQCPSPPSFVPNMFSPFTDNMTFLQRVANMLIRSMDFLLCKYVYSQFDDLASEFLHKDVTLLDLFSKSPVWLLRYDFVFEYPRPTMP   268
l1a4        FLRGIPCGYEFEATQCPRPPSYVPRGFTRYSDHMTFPQRVKNMLFHFTEFFLCSSVYTPYAKLASEFLQREMTFVELVSNGSVWLIREDLAFTYPKPLMP   266
l1a5        FLRGIPCGYEFEATQCPRPPSYVPRALTRYSDHMTFLQRVKNMLFHLTEFFLCSFVYSPYAKLASEFLQRDMTFAELISNGSVWLIREDFAFTYPKPLMP   266
zf1a3       FLQQIPCGLEYQATQCPNPPSYVPRVFTELTDHMTFLQRVKNMLYDIPNFFLCDVVFQPYAELASEFFKQEVTVPDLLRQASVWLMKLDFVLHFPKPLMP   267
zf1a7       FLRGVPCGLDLDDRLCPSPPSYVPRVFTDLTDHMTFFQRVKNLLFEIPNVFLCDFAFQPYSKLASEFLQREVTVQDLLRKGSVWLLRLDFVLDYPRPLMP   267
zf1a8       FLRGLHCGLDFEATQCPRPPSYVPRGFTQFTDHMAFLQRVKNLLYDIPDSFICDFAFDPFSKMASEFLQQDVTVQDLLRQASVWLLKLDFVHDYPRPLMP   267
h1a1        FLHALPCSLEFEATQCPNPFSYVPRPLSSHSDHMTFLQRVKNMLIAFSQNFLCDVVYSPYATLASEFLQREVTVQDLLSSASVWLFRSDFVKDYPRPIMP   270
h1a3        FLRNIPCDLDFKGTQCPNPSSYIPRLLTTNSDHMTFMQRVKNMLYPLALSYICHAFSAPYASLASELFQREVSVVDILSHASVWLFRGDFVMDYPRPIMP   271
h1a4        FWRYIPCDLDFKGTQCPNPSSYIPKLLTTNSDHMTFLQRVKNMLYPLALSYICHTFSAPYASLASELFQREVSVVDLVSYASVWLFRGDFVMDYPRPIMP   271
h1a5        FLRNIPCDLDFKGTQCPNPSSYIPRLLTTNSDHMTFLQRVKNMLYPLALSYLCHAVSAPYASLASELFQREVSVVDLVSHASVWLFRGDFVMDYPRPIMP   271
zf1a10      FLRGFPCGMDSAATQCPSPPSYVPRLFLDNTDSMMFSQRVKNMLVNLLELFYCKPIYDNFEELAYKVFKKKVTATELLSRGSFWLMRYDFVFEFPRPVMP   268
h1a6        LFRGFPCSLEHTFSRSPDPVSYIPRCYTKFSDHMTFSQRVANFLVNLLEPYLFYCLFSKYEELASAVLKRDVDIITLYQKVSVWLLRYDFVLEYPRPVMP   269
l1a6        FFRGFPCNLELAVSKCPDPVSYVPRCYTAYTDYMTFRQRLLNLFVSLLETTLFKILYSRYQEIASELFQREVHLPTLYRNGSIWLLRYDFVFEYPRPVMP   270
l1a7        FFRGFPCSLEHAICKTPNPVSYIPRCYTRHTDRMSFFQRVINLIVNSFETSLFKMLYTKYQDIASEFLQRDVHLPTLYRNGSIWLLRYDFVFEFPRPLMP   270
zf1a11      FMRGFGCNLHFAAPQCPSPPSYTPRLFSFNSDHMNFFQRVENALILLLELEYCNSFYAEALKLSSEVLQRDVSLMDLVNSAAVWLLRFDFVFEYVRPVMP   265
h1a9        FARGILCHYLEEGAQCPAPLSYVPRILLGFSDAMTFKERVRNHIMHLEEHLLCHRFFKNALEIASEILQTPVTEYDLYSHTSIWLLRTDFVLDYPKPVMP   267
h1a7        FARGIFCHYLEEGAQCPAPLSYVPRLLLGFSDAMTFKERVWNHIMHLEEHLFCPYFFKNVLEIASEILQTPVTAYDLYSHTSIWLLRTDFVLEYPKPVMP   267
h1a10       FTRGIFCHHLEEGAQCPAPLSYVPNDLLGFSDAMTFKERVWNHIVHLEDHLFCQYLFRNALEIASEILQTPVTAYDLYSHTSIWLLRTDFVLDYPKPVMP   267
h1a8        FARGIACHYLEEGAQCPAPLSYVPRILLGFSDAMTFKERVRNHIMHLEEHLFCQYFSKNALEIASEILQTPVTAYDLYSHTSIWLLRTDFVLDYPKPVMP   267
z1a1        FLRLIPCRLDEAAAQCPSPPSFMPRYSSGFTDKMTFPQRLVNTLLTVVEGFLCRSMYESADELASRYLQKETTYAELLGHGAVWLLRYDFAFEFPRPQMP   268
z1a3        FLRGIPCRLDEAAAQCPSPPSFIPRFFTGYTDKMTFSQRMINTFMTVFEKYLCHQLFASFDELATRYLKKDTSYAELLGHGAVWLLRYDFSFEYPKPQMP   269
z1b1        FLRGMPCGIDLLASQCPSPPSYVPRFHSGTSDKMNFVERIRNFFMSGFELVLCKVMYASFDELAARYLKKDVTYKEIIGRGALWLHRYDFTFEYPRPIMP   271
m1a1        FLRGLPCELDYKANQCPSPPSFVPVFYSGSTDKMNFLQRVKNFFMYNVQSYVCTVLYQHFDDLVSRRIESGMSYKDLLSHGAIWLFRYDFTFEWPRPLMP   269
s1a1        FLRGLPCELDSRANHCPAPVSYVPVAFTGNTDIMNFPQRVKNMLMSILESYVCRVLYYNFEDLVSRYLGNDITYKGLLSNGAIWLLRYDFTFEWPKPLLP   269
f1a1        FLRGLPCELDLKANQCPAPPSYVPMAFSGNSDVMNFPQRVKNMLMYFVQSYMCKIIYREFDRLVTRHMSDVQSYRELLSRGAFWLLRYDFTFEYPRPVMP   269
t1a1        FLRGLPCELDSKANQCPAPPSYVPVAFSGNSDVMTFPQRVKNMLMYFVESYLCKVMYREFDRLVTRHMSDIQSYRELISRGAFWLLRYDFTFEYPKPVMP   269
z1a2        MQINHPCGVDTLASQCPAPPSYVPQTFTHFTDRMNLWQRSINFVRALLQPKACKQIFARADEIASHLLQRKTSMVEIFSRAALWFLRFNFALEFPRPVMP   263
z1a4        MQVNLPCGVDSLASQCPSPPSYVPQRNTHFTDQMNIWQRCTNLVRTLLQHMACRYMYTEADEIASRFLQRRASIVEIMNRATLWLMRFDFAFEFPRPLMP   263
z1a5        IQTSHPCGADTLASQCPSPPSYVPQRLTHFSDRMNLWQRSVNFVRTLIQPMACRRLFTRADEIASRVLQRKTSIMEIISCAALWFVHSDFALEFPHPLMP   262
z1a6        IQTSHPCNVDTLASQCPSPPSYVPHILTHFSDRMNLWQRSVNFVRTLIQPMACRRLFTRADEIASRVLQRKTSIMEIMSRAALWFVHSDFALEFPHPLMP   263
z1a7        MQTSHPCGADVLASQCPAPPSYVPKGLTHFTDRMNLWQRSVNFVRTLVQPVACSRMFAHADEIASKVLQKKTSVMEIMSRAALWFMHFDFAFEFPRPVMP   270
z1b2        LLRGMACGLDATATTCPNPSSYIPRFHTRNTDRMSFGERVMNMLMSILEQTVCKFMYRSFEEMIFNFLQRDVSMTEILRTGAVWLMRYDFTLEFPKPLMP   274
z1b3        MLRGMPCALDATATACPNTPSYIPRFHTGNTDRMSFGERVMNVLMSILEQVACKVMYWSFEEVTPNFLQRDVSLTEILSTGAVWLMRYDFTLEFPKPLMP   274
z1b4        MLRGIMC--ESKATACPNPPSYIPRFFTQISDRMTFSERVINTLVSLLEPLMCRLMLGSFEEMTSKFLHRDVSITEILSTAAVWLMRYDFTLEFPKPLMP   272
z1b5        MLRGLMC--ESKATACPDPPSYIPRLFTQNSDHMNFGERLMNTLASLMEPLMCKLKFSAFEEVTSKFLHRDVSMTEILSTGAVWLMRYDFTLEFPKPLMP   271
z1b7        MLRGLPCGLDSVATACPNPPSYIPRFFTHSSDRMSFGQRVLNVLVSMLEPLLCRLIYWTTEDVASRFMQRDVSVTEVLSSGALWLLRYDFTLEFPKPLMP   273
f1b1        LLRGIPCMLDMKSAGCPSPPSYVPRYMTGYTDKMNFKERTINTMVALLEPLMCKLLFWQFDYISHQFLGEEVGMAEVLSESAVWLLRIDMTLELPRPLMP   296
f1b2        LLRGIPCLLDMKSAGCPSPPSYVPRFFTGYTDKMNFKERTINTMVALLEPLMCKLLFWQFDYISHQFLGEEVSIAEVLSESAVWLLRIDMTLELPRPLMP   296
t1b1        LLRGIPCLMDMKAAGCPSPPSYVPRFMTGYTDKMTFRERTINTLIALLEPLMCKLFFWHFDNISHQFLGEKVGIAEVLAESDVWLLRIDMTLELPRPLMP   292
s1b1        LLRGIPCSLDMKSAGCPSPPSYVPRFLTKYSHVMNFKERTVNTLVALLEPLLCRLMYWYFDQIAYRFLGEEVGVAEVLSESAIWLLRMDFTLEFPRPIMP   290
m1b1        LLRGIPCSLDMKAAGCPSPPSFVPRFFTGLSDRMNFKERLINTLVALLEPLFCRLLFWRFNNIAHKFLKEEVSVAEVLSESDIWLLRIDFTLEFPRPLMP   290
m1b2        LLRGIPCALDMKAAGCPSPPSFVPRPFTGLSDRMNFKERLINTLVALLEPLFCRLLFWRFNNIAHKFLNEEVSVAEILSESDIWLLRIDFTLEFPRPLMP   288
```

```
                   *   :..        :  :.   :.    .*::**::*: .   *   :  :  .:  :** *.**: *       .*. .:.*:******. .  :.*:
x1a1        NMVFVGGINCVGEQHLNQEFEKLVNSSGEHGFVVFSLGSMVSEIPMNKAMDIAEALKSIPQKVFWRYTGKAPPNLGENTHLVKWLPQNDLLAHPKARAFI    371
x1a3        NMVYIGGINCAGTKPLSQEFEKLVNSSGEHGFVVFSLGSMVSEIPMNKAMDIAEALKSIPQKVFWRYTGKAPPNLGENTHLVKWLPQNDLLAHPKARAFI    368
x1a4        NMVYIGGINCAGTKPLSWEFEKLVNSSGEHGFVVFSLGSMVSEIPMNKAMDIAEALKSIPQKVFWRYTGKAPPNLGENTHLVKWLPQNDLLAHPKARAFI    368
x1a5        NVILIGGINCAKQKALPQEFEKLVKSSGEHGFVVFSFGSVVSEIPMNIAMDIAEALRYIPQKVFWRYTGKAPSNLGDNTHLVKWLPQNDLLAHPKARAFI    370
x1a6        NVILIGGINCAKQKALPQEFEKLVKSSGEHGFVVFSFGSMVSEIPMNIAMDIAEALRYIPQKVFWRYTGKAPPNLGDNTHLVKWLPQNDLLAHPKARAFI    370
x1a2        NMIYIGGDTCKEKEKLPHEFEKLVNSSGEHGFVVFSLGSMVSEIPMNKAMDIAEALKSIPQKVFWRYTGKAPPNLGENTHLVKWLPQNDLLAHPKARAFI    360
l1a1        NMVFIGGINCQRKSPLSQEFEAIVNESGEHGIVVFSLGSMVSEIPMKKAMEIAEGLGTIPQTVLWRYTGETPPNLAKNTKLVKWLPQNDLLAHPKTRAFI    367
l1a2        NMIYIGGITCAQKKPLSKEFEAIVNESGEHGIVVFSLGSMVSEIPMKKAMEIAEGLGTIPQTVLWRYTGETPPNLAKNTKLVKWLPQNDLLAHPKTRAFI    369
zf1a1       NMVLVGGIGCTRENKLSQEFEAMVNASGEHGIVVFSLGSMVSEIPMKKAMEIAEGLGKVPQMVLWRYTGKAPPNLPKNVKLVKWLPQNDLLAHPKTRAFI    366
zf1a2       NMVYIGGINCLQKKPLSKEFEAMVNASGEHGIVVFSLGSMVSEIPMKKAMEIAEGLGKVPQMVLWRYTGKAPPNLPKNVKLVKWLPQNDLLAHPKTRAFI    364
l1a3        NMVFIGGINCVQKKNLTQEFEAIVNESGEHGIVVFSLGSMVSEIPMKKAMEIAEGLGTIPQTVLWRYTGETPPNLAKNTKLVKWLPQNDLLAHPKTRAFI    368
l1a4        NMIMIGGINCAGKKPLSQEFEAIVNESGEHGIVVFSLGSMVSEIPMKKAMEIAEGLGTIPQTVLWRYTGETPPNLAKNTKLVKWLPQNDLLAHPKTRAFI    366
l1a5        NMIMIGGINCAGKEPLSQEFEAIVNESGEHGIVVFSLGSMVSEIPMKKAMEIAEGLGTIPQTVLWRYTGETPPNLAKNTKLVKWLPQNDLLAHPKTRAFI    366
zf1a3       NMVLISGVNCAYK-KLNQEFEAMVNASGEHGIVVFSLGSMVSEIPMKKAMEIAEGLGKVPQMVLWRYTGKAPPNLPKNVKLVKWLPQNDLLAHPKTRAFI    366
zf1a7       NIIPIGGVNCAHK-ELPQEFEAMVNASGEHGIVVFSLGSMVSEIPMKKAMEIAEGLGKVPQMVLWRYTGKAPPNLPKNVKLVKWLPQNDLLAHPKTRAFI    366
zf1a8       NIIPIGGVNCAHK-ELPQEFEAMVNASGEHGIVVFSLGSMVSEIPMKKAMEIAEGLGKVPQMVLWRYTGKAPPNLPKNVKLVKWLPQNDLLAHPKTRAFI    366
h1a1        NMVFVGGINCLHQNPLSQEFEAYINASGEHGIVVFSLGSMVSEIPEKKAMAIADALGKIPQTVLWRYTGTRPSNLANNTILVKWLPQNDLLGHPMTRAFI    370
h1a3        NMVFIGGINCANRKPLSQEFEAYINASGEHGIVVFSLGSMVSEIPEKKAMAIADALGKIPQTVLWRYTGTRPSNLANNTILVKWLPQNDLLGHPMTRAFI    371
h1a4        NMVFIGGINCANGKPLSQEFEAYINASGEHGIVVFSLGSMVSEIPEKKAMAIADALGKIPQTVLWRYTGTRPSNLANNTILVKWLPQNDLLGHPMTRAFI    371
h1a5        NMVFIGGINCANGKPLSQEFEAYINASGEHGIVVFSLGSMVSEIPEKKAMAIADALGKIPQTVLWRYTGTRPSNLANNTILVKWLPQNDLLGHPMTRAFI    371
zf1a10      NMAFIGGINCVQKNKLSQEFEAMVNASGEHGIVVFSLGSMVSEIPMKKAMEIAEGLGKVPQMVLWRYTGKAPPNLPKNVKLVKWLPQNDLLAHPKTRAFI    368
h1a6        NMVFIGGINCKKRKDLSQEFEAYINASGEHGIVVFSLGSMVSEIPEKKAMAIADALGKIPQTVLWRYTGTRPSNLANNTILVKWLPQNDLLGHPMTRAFI    369
l1a6        NMVFIGGINCEEGKRLSQEFEAIVNESGEHGIVVFSLGSMVSEIPMKKAMEIAEGLGTIPQTVLWRYTGETPPNLAKNTKLVKWLPQNDLLAHPKTRAFI    370
l1a7        NMAFIGGINCEEGKDLPQEFEAIVNESGEHGIVVFSLGSMVSEIPMKKAMEIAEGLGTIPQTVLWRYTGETPPNLAKNTKLVKWLPQNDLLAHPKTRAFI    370
zf1a11      NMVFVGGINCAKKKPLPKEFEAMVNASGEHGIVVFSLGSMVSEIPMKKAMEIAEGLGKVPQMVLWRYTGKAPPNLPKNVKLVKWLPQNDLLAHPKTRAFI    365
h1a9        NMIFIGGINCHQGKPLPMEFEAYINASGEHGIVVFSLGSMVSEIPEKKAMAIADALGKIPQTVLWRYTGTRPSNLANNTILVKWLPQNDLLGHPMTRAFI    367
h1a7        NMIFIGGINCHQGKPVPMEFEAYINASGEHGIVVFSLGSMVSEIPEKKAMAIADALGKIPQTVLWRYTGTRPSNLANNTILVKWLPQNDLLGHPMTRAFI    367
h1a10       NMIFIGGINCHQGKPLPMEFEAYINASGEHGIVVFSLGSMVSEIPEKKAMAIADALGKIPQTVLWRYTGTRPSNLANNTILVKWLPQNDLLGHPMTRAFI    367
h1a8        NMIFIGGINCHQGKPLPMEFEAYINASGEHGIVVFSLGSMVSEIPEKKAMAIADALGKIPQTVLWRYTGTRPSNLANNTILVKWLPQNDLLGHPMTRAFI    367
z1a1        NMVQIGGINCVKRAPLTKELEEFVNGSGEHGFVVFTLGSMVSQLPEAKAREFFEAFRQIPQRVLWRYTGPVPENAPKNVKLMKWLPQNDLLGHPKVRAFV    368
z1a3        NMVQIGGINCAKRAPLTKELEEFVNGSGEHGFVVFTLGSMVSQLPEAKAREFFEAFRQIPQRVLWRYTGPVPENAPKNVKLMKWLPQNDLLGHPKVRAFV    369
z1b1        NMVFIGGINCQKSAEISAEVEEFVNGSGEHGIVVFSLGSLVSSMPKEKADIFFKAFSMIPQRVLWRYTGEIPDNVPENVKLMKWLPQNDLLGHPKARAFI    371
m1a1        NMVLIGGINCAKKAPLPTDLQEFVDGSGDDGFIVFTLGSMVSTMPAEKAKQFFDAFRQIPQRVLWRYTGELPEDMPKNVKVMSWLPQNDLLAHPKIKVFI    369
s1a1        NMVFIGGINCAKKAPLPADLKEFVEGSGDDGFIVFTLGSMVSDMPKKKAKQFLDAFRQIPQRVVWRYTGNFTEDVPKNVKLMKWLPQNDLLAHPKAKVFM    369
f1a1        NTAFIGGINCAKKAALPADLEEFVNGSGDDGFIVFTLGSMVENMPEQKAKEFFTAFGQVPQRVLWRYNGAVPENAPKNVKVMKWLPQNDLLAHPKAKVFI    369
t1a1        NTAFIGGINCVKKAPLPADLEEFVNGSEDHGFIVFTLGSMVDNMPEEKAKQFFDAFAQIPQRVLWRYNGAVPENVPKNVKLMKWLPQNDLLAHPKAKVFV    369
z1a2        NMVMIGATVNQKPKPLTHELEEFVNGSGEHGFVVFTLGSMVSQLPEAKAREFFEAFRQIPQRVLWRYTGPVPENAPKNVKLMKWLPQNDLLGHPKVRAFV    363
z1a4        NMVMIGGMATKMPKPLSKELEEFVNGSGEHGFVVFTLGSMVSQLPEAKAREFFEAFRQIPQRVLWRYTGPVPENAPKNVKLMKWLPQNDLLGHPKVRAFV    363
z1a5        NMIIVGGMDNRKAEPLSQELEEFVNGSGEHGFVVFTLGSMVSQLPEAKAREFFEAFRQIPQRVLWRYTGPVPENAPKNVKLMKWLPQNDLLGHPKVRAFV    362
z1a6        NMIIVGGMDNRKAEPLSQELEEFVNGSGEHGFVVFTLGSMVSQLPEAKAREFFEAFRQIPQRVLWRYTGPVPENAPKNVKLMKWLPQNDLLGHPKVRAFV    363
z1a7        NMVVIGGVDTKKPEPLSQELEEFVNGSGEHGFVVFTLGSMVSQLPEAKAREFFEAFRQIPQRVLWRYTGPVPENAPKNVKLMKWLPQNDLLGHPKVRAFV    370
z1b2        NMQFIGGINCGVRNPLTKEVEEFVNGSGEHGIVVFSLGSLVSSMPKEKADIFFKAFSMIPQRVLWRYTGEIPNNVPENVKLMKWLPQNDLLGHPKARAFI    374
z1b3        NMQFIGGINCGVKNPLMKEVEEFVNGSGEHGIVVFSLGSLVSSMPKEKADIFFKAFSMIPQRVLWRYTGEIPNNVPENVKLMKWLPQNDLLGPPKARAFI    374
z1b4        NMITIGGINCEVKNPLTKEVEEFVNGSGEHGIVVFSLGSLVSSMPKEKADIFFKAFSMIPQRVLWRYTGEIPNNVPENVKLMKWLPQNDLLGHPKARAFI    372
z1b5        NITNIGGINCEVNNPLTKEVEEFVNGSGEHGIVVFSLGSLVSSMPKEKADIFFKAFSMIPQRVLWRYTGEIPNNVPENVKLMKWLPQNDLLGHPKARAFI    371
z1b7        NMVLIGGINCAIRHPLTKEVEEFVNGSGEHGIVVFSLGSLVSSMPKEKADIFFKAFSMIPQRVLWRYTGEIPNNVPENVKLMKWLPQNDLLGHPKARAFI    373
f1b1        NTILVGGINCNVRDALPEDLLPWVS--GEHGFIVFTLGSMVSEMPEETTTIFLEAFRQIPQKVIWRYTGQIDGNVPDNVKIMKWVPQNDLLAHPGARAFI    394
f1b2        NTILVGGINCNVRDALPEDLLPWVS--GEHGFIVFTLGTAVSEMPEETTTIFLEAFRQIPQKVIWRYTGQIDGNLPDNVKIMKWVPQNDLLAHPGARAFI    394
t1b1        NTILVGGINCNVRDALPEDLLPWVS--GEHGFIVFTLGSMVSEMPEEITSVFIEAFRQIPQKVIWRFTGEIGGSIPENVKIMKWVPQNDLLAHPGARAFI    390
s1b1        NVVLVGGINCNVRNPLPEDLESWLS--GEHGFVVFTLGTMVSDLPEEITSVLLEAFRQIPQKVIWRYTGKVPDRVPENVKFMTWVPQNDLLAHSGARAFI    388
m1b1        NMILVGGINCDLRKPLPQDLEQWVS--GRHGFIVFTLGSMIPDMPEEITSVFIEAFRQIPQTVIWRYAGKVPDNIPKNVKLMKWVPQNDLLAHPGARAFI    388
m1b2        NMILVGGINCYLRKPLPQDLEQWVS--GEHGFIVFTLGSVFSETPEEITSVFVEAFRQIPQTVIWRYTGKVPDNLPNNVKMMKWVPQNDLLAHSGVRAFI    386
```

```
              * *:** :*.:*..***::.*: .:* :*. ::  :...  * :  :  . :  .:  :  :  **:.:  :* :: *:*: ** *: .*.*.**:*:*
x1a1          THAGSHGIYEGICNAVPMVMMPLFGDQMDNAKRIESRGAGLTLNVLHMTPEDLSNAVRAVINNPVYKENIQRMSSLHLDRPIHPLDLAVHWVEFVMRHKG   471
x1a3          THAGSHGIYEGICNAVPMVMMPLFGDQMDNAKRIESRGAGLTLNVLHMTPEDLSNAVRAVINNPVYKENIQRMSSLHLDRPIHPLDLAVHWVEFVMRHKG   468
x1a4          THAGSHGIYEGICNAVPMVMMPLFGDQMDNAKRIESRGAGLTLNVLHMTPEDLSNAVRAVINNPVYKENIQRMSSLHLDRPIHPLDLAVHWVEFVMRHKG   468
x1a5          THAGSHGLYEGICNAVPMVLMPLFGDQMDNAKRIESRGAGLILNVQNLIPDDLSKAVMAVIDNPSYKENIQRLSSLHRDRPVQPLDLAVHWVEFVMKHKG   470
x1a6          THAGSHGVYEGICNAVPMVMLPLIGDQMDNAKRIESRGAGLILNVQNLIPDDLSNAVMAVIDNPSYKENIQRLSSLHRDRPVQPLDLAVHWVEFVMKHKG   470
x1a2          THAGSHGIYEGICNAVPMVMMPLFGDQMDNAKRIESRGAGLTLNVLHMTPEDLSNAVRAVINNPVYKENIQRMSSLHLDRPIHPLDLAVHWVEFVMRHKG   460
l1a1          THAGSHGVYEGICNGVPMVLMPLFGDQMDNAKRVESRGAGVALNIIEMTAKDISEALKAVIYDKKYKENIQRLSELHLDRPIHPLDLAVHWVEFVMKHKG   467
l1a2          THAGSHGVYEGICNGVPMVLMPLFGDQMDNAKRVESRGAGVALNIIEMTAKDISEALKAVIYDKKYKENIQRLSELHLDRPIHPLDLAVHWVEFVMKHKG   469
zf1a1         THGGSHGVYEGICNAVPMVLMPLFGDQMDNAKRVESRGAGLTLNILEMTSNDISNALKAVINDKKYKENIQRLSDLHLDRPIHPLDLAVHWVEFVMRHKG   466
zf1a2         THGGSHGVYEGICNAVPMVLMPLFGDQMDNAKRVESRGAGLTLNILEMTSNDISNALKAVINDKKYKENIQRLSDLHLDRPIHPLDLAVHWVEFVMRHKG   464
l1a3          THAGSHGVYEGICNGVPMVLMPLFGDQMDNAKRVESRGAGVALNIIEMTAKDISEALKAVIYDKKYKENIQRLSELHLDRPIHPLDLAVHWVEFVMKHKG   468
l1a4          THAGSHGVYEGICNGVPMVLMPLFGDQMDNAKRVESRGAGVALNIIEMTAKDISEALKAVIYDKKYKENIQRLSELHLDRPIHPLDLAVHWVEFVMKHKG   466
l1a5          THAGSHGVYEGICNGVPMVLMPLFGDQMDNAKRVESRGAGVALNIIEMTAKDISEALKAVIYDKKYKENIQRLSELHLDRPIHPLDLAVHWVEFVMKHKG   466
zf1a3         THGGSHGVYEGICNAVPMVLMPLFGDQMDNAKRVESRGAGLTLNILEMTSNDISNALKAVINDKKYKENIQRLSDLHLDRPIHPLDLAVHWVEFVMRHKG   466
zf1a7         THGGSHGVYEGICNAVPMVLMPLFGDQMDNAKRVESRGAGLTLNILEMTSNDISNALKAVINDKKYKENIQRLSDLHLDRPIHPLDLAVHWVEFVMRHKG   466
zf1a8         THGGSHGVYEGICNAVPMVLMPLFGDQMDNAKRVESRGAGLTLNILEMTSNDISNALKAVINDKKYKENIQRLSDLHLDRPIHPLDLAVHWVEFVMRHKG   466
h1a1          THAGSHGVYESICNGVPMVMMPLFGDQMDNAKRMETKGAGVTLNVLEMTSEDLENALKAVINDKSYKENIMRLSSLHKDRPVEPLDLAVFWVEFVMRHKG   470
h1a3          THAGSHGVYESICNGVPMVMMPLFGDQMDNAKRMETKGAGVTLNVLEMTSEDLENALKAVINDKSYKENIMRLSSLHKDRPVEPLDLAVFWVEFVMRHKG   471
h1a4          THAGSHGVYESICNGVPMVMMPLFGDQMDNAKRMETKGAGVTLNVLEMTSEDLENALKAVINDKSYKENIMRLSSLHKDRPVEPLDLAVFWVEFVMRHKG   471
h1a5          THAGSHGVYESICNGVPMVMMPLFGDQMDNAKRMETKGAGVTLNVLEMTSEDLENALKAVINDKSYKENIMRLSSLHKDRPVEPLDLAVFWVEFVMRHKG   471
zf1a10        THGGSHGVYEGICNAVPMVLMPLFGDQMDNAKRVESRGAGLTLNILEMTSNDISNALKAVINDKKYKENIQRLSDLHLDRPIHPLDLAVHWVEFVMRHKG   468
h1a6          THAGSHGVYESICNGVPMVMMPLFGDQMDNAKRMETKGAGVTLNVLEMTSEDLENALKAVINDKSYKENIMRLSSLHKDRPVEPLDLAVFWVEFVMRHKG   469
l1a6          THAGSHGVYEGICNGVPMVLMPLFGDQMDNAKRVESRGAGVALNIIEMTAKDISEALKAVIYDKKYKENIQRLSELHLDRPIHPLDLAVHWVEFVMKHKG   470
l1a7          THAGSHGVYEGICNGVPMVLMPLFGDQMDNAKRVESRGAGVALNIIEMTAKDISEALKAVIYDKKYKENIQRLSELHLDRPIHPLDLAVHWVEFVMKHKG   470
zf1a11        THGGSHGVYEGICNAVPMVLMPLFGDQMDNAKRVESRGAGLTLNILEMTSNDISNALKAVINDKKYKENIQRLSDLHLDRPIHPLDLAVHWVEFVMRHKG   465
h1a9          THAGSHGVYESICNGVPMVMMPLFGDQMDNAKRMETKGAGVTLNVLEMTSEDLENALKAVINDKSYKENIMRLSSLHKDRPVEPLDLAVFWVEFVMRHKG   467
h1a7          THAGSHGVYESICNGVPMVMMPLFGDQMDNAKRMETKGAGVTLNVLEMTSEDLENALKAVINDKSYKENIMRLSSLHKDRPVEPLDLAVFWVEFVMRHKG   467
h1a10         THAGSHGVYESICNGVPMVMMPLFGDQMDNAKRMETKGAGVTLNVLEMTSEDLENALKAVINDKSYKENIMRLSSLHKDRPVEPLDLAVFWVEFVMRHKG   467
h1a8          THAGSHGVYESICNGVPMVMMPLFGDQMDNAKRMETKGAGVTLNVLEMTSEDLENALKAVINDKSYKENIMRLSSLHKDRPVEPLDLAVFWVEFVMRHKG   467
z1a1          THGGSHGIYEGICNGVPMVMLPLFGDQGDNAQRLVSRGVAESLTIYDVTSEKLLVALKKVINDKSYKEKMMKLSAIHRDRPIEPLDLAVFWTEFVMRHKG   468
z1a3          THGGSHGIYEGICNGVPMVMLPLFGDQGDNAQRLVSRGVAESLTIYDVTSEKLLVALKKVINDKSYKEKMMKLSAIHRDRPIEPLDLAVFWTEFVMRHKG   469
z1b1          THGGTHGIYEGICRGVPMVMLPLFGDQADNVHRVATRGVGVILSIHDITVETLLDALNSVINNSSYKQKMQKLSAIHNDRPIQPLDLAVFWTEFVMRHKG   471
m1a1          THGGTHGIYEGICNGVPMLMFPLFGDQGDNVHRLVARGVAEKLSMLDVTTETLLAALKKLIYDKSYKERMATLSSIHLDRPIEPLELATFWTEFVMRHKG   469
s1a1          THGGTHGIYEGICNAVPMLMFPLFGDQGDNVHHLVSRGVAEKLLIYDVTTETLLVAIKKIINDKSYKEKITTLSDIHLDRPVEPLDLAVFWTEFVMRHKG   469
f1a1          THGGTHGIYEGICNGVPMLMFPLFGDQGDNVNHLVHRGVAERLGIYDVTSEKVVAALKKMTHDKSYKEKIVKLSQINQDRPVAPLELSVFWTEFVMRHKG   469
t1a1          THGGAHGIYESICNGVPMLMFPLFGDQSDNVQRMVHRGVAETLSIYDVTSQKLVAALKKMVQDKSYKENMVTLSQLNQDRPVAPLDLAVFWTEFVMRHQG   469
z1a2          THGGSHGIYEGICNGVPMVMLPLFGDQGDNAQRLVSRGVAESLTIYDVTSEKLLVALKKVINDKSYKEKMMKLSAIHRDRPIEPLDLAVFWTEFVMRHKG   463
z1a4          THGGSHGIYEGICNGVPMVMLPLFGDQGDNAQRLVSRGVAESLTIYDVTSEKLLVALKKVINDKSYKEKMMKLSAIHRDRPIEPLDLAVFWTEFVMRHKG   463
z1a5          THGGSHGIYEGICNGVPMVMLPLFGDQGDNAQRLVSRGVAESLTIYDVTSEKLLVALKKVINDKSYKEKMMKLSAIHRDRPIEPLDLAVFWTEFVMRHKG   462
z1a6          IHGGSHGIYEGICNGVPMVMLPLFGDQGDNAQRLVSRGVAESLTIYDVTSEKLLVALKKVINDKSYKEKMMKLSAIHRDRPIEPLDLAVFWTEFVMRHKG   463
z1a7          THGGSHGIYEGICNGVPMVMLPLFGDQGDNAQRLVSRGVAESLTIYDVTSEKLLVALKKVINDKSYKEKMMKLSAIHRDRPIEPLDLAVFWTEFVMRHKG   470
z1b2          THGGTHGIYEGICHGVPMVMLPLFGDQADNVHRVATRGVGVILSIHDITVETLLDALNSVINNSSYKQKMQKLSAIHNDRPIQPLDLAVFWTEFVMRHKG   474
z1b3          THGGTHGIYEGICHGVPMVMLPLFGDQADNVHRVATRGVGVILSIHDITVETLLDALNSVINNSSYKQKMQKLSAIHNDRPIQPLDLAVFWTEFVMRHKG   474
z1b4          THGGTHGIYEGICHGVPMVMLPLFGDQADNVHRVATRGVGVILSIHDITVETLLDALNSVINNSSYKQKMQKLSAIHNDRPIQPLDLAVFWTEFVMRHKG   472
z1b5          THGGTHGIYEGICHGVPMVMLPLFGDQADNVHRVATRGVGVILSIHDITVETLLDALNSVINNSSYKQKMQKLSAIHNDRPIQPLDLAVFWTEFVMRHKG   471
z1b7          THGGTHGIYEGICHGVPMVMLPLFGDQADNVHRVATRGVGVILSIHDITVETLLDALNSVINNSSYKQKMQKLSAIHNDRPIQPLDLAVFWTEFVMRHKG   473
f1b1          THAGSHGLYEGLCHAVPMVMVPLIAEQPDNAEKMASRGAGIVLNILSVSTEDIVQALNNVINDTRYKDNIKTLSELHKDQPINPLELSVYWTEFVMRHKG   494
f1b2          THAGSHGLYEGLCHAVPMVMVPLLAEQPDNAEKMASRGAGILLNIFSVTTEDIVQALNNVINDTRYKDNIKTLSELHKDQPINPLELSVYWTEFVMRHKG   494
t1b1          THAGSHGLYEGLCHAVPMVMVPVSAEQPDNAEKMASRGAGIVLNILTVTSENIVQALNAVINDTRYKENVKKLSELHKDRPVDPLELSVYWTEFVMRHKG   490
s1b1          THAGSHGLFEGLCHAVPMLMMPLGGDQPDNAERLASRNAGLVLDITTISTESLLQGLNAVINDTRYKENMKKLSALHKDRPVDPLDLSVYWTEHVMRHKG   488
m1b1          THAGSHGTFEGLCHAVPMLMFPIAGDQPDNAAKMANRGVGVVLDISTVTTEGLLQGLNELINNTRYKENIQRLSALHKDRPVDPLGLSSFWTEFVMRHKG   488
m1b2          THTGSHGTFEGLCHAVPMLMFPILGDQTENAVRMAKKGAGIMLDISTVTTEGLLQGLNELINNTRYKENIQRLSALHKDRPVDPLELSVFWTEFVMRHKG   486
```

```
              : **: * : *.  ** .:*:  .:    :      .*
x1a1          APHLRPAAHDLNWFQYYSLDVIGFLLAVLLTALFISLKCCTFVFRRCFKRNSKKQRKSKSQ--   532
x1a3          APHLRPAAHDLNWFQYYSLDVIGFLLAVLLTALFISLKCCTFVFRRCFKRNSKKQRKSKSQ--   529
x1a4          APHLRPAAHDLNWFQYYSLDVIGFLLAVLLTALFISLKCCTFVFRRCFKRNSKKQRKSKSQ--   529
x1a5          APHLRPAAHDLNWIQYQSLDVMAFLLAVLLTTLFISLKCCTFACRCCCKRNTKPKRKTKSE--   531
x1a6          APHLRPAAHDLNWIQYQSLDVMAFLLAVLLTTLFISLKCSTFACRCCCKRNTKPKRKTKSE--   531
x1a2          APHLRPAAHDLNWFQYYSLDVIGFLLAVLLTALFISLKCCTFVFRRCFKRNSKKQRKSKSQ--   521
l1a1          APHLRPAAHDLNWVQYHSIDVLAFLLAASLLALFISMKCCMFCCRKCFCKSGRLTKKSKSKSH   530
l1a2          APHLRPAAHDLNWVQYHSIDVLAFLLAASLLALFISMKCCMFCCRKCFCKSGRLTKKSKSKSH   532
zf1a1         APHLRPAAHDLNWVQYHSLDVIAFLAAITLLFLFISFKCCLCCCRCCFCKKGRTGKATKAKSH   529
zf1a2         APHLRPAAHDLNWVQYHSLDVIAFLAAITLLFLFISFKCCLCCCRCCFCKKGRTGKATKAKSH   527
l1a3          APHLRPAAHDLNWVQYHSIDVLAFLLAASLLALFISMKCCMFCCRKCFCKSGRLTKKSKSKSH   531
l1a4          APHLRPAAHDLNWVQYHSIDVLAFLLAASLLALFISMKCCMFCCRKCFCKSGRLTKKSKSKSH   529
l1a5          APHLRPAAHDLNWVQYHSIDVLAFLLAASLLALFISMKCCMFCCRKCFCKSGRLTKKSKSKSH   529
zf1a3         APHLRPAAHDLNWVQYHSLDVIAFLAAITLLFLFISFKCCLCCCRCCFCKKGRTGKATKAKSH   529
zf1a7         APHLRPAAHDLNWVQYHSLDVIAFLAAITLLFLFISFKCCLCCCRCCFCKKGRTGKATKAKSH   529
zf1a8         APHLRPAAHDLNWVQYHSLDVIAFLAAITLLFLFISFKCCLCCCRCCFCKKGRTGKATKAKSH   529
h1a1          APHLRPAAHDLTWYQYHSLDVIGFLLAVVLTVAFITFKCCAYGYRKCLGKKGRVKKAHKSKTH   533
h1a3          APHLRPAAHDLTWYQYHSLDVIGFLLAVVLTVAFITFKCCAYGYRKCLGKKGRVKKAHKSKTH   534
h1a4          APHLRPAAHDLTWYQYHSLDVIGFLLAVVLTVAFITFKCCAYGYRKCLGKKGRVKKAHKSKTH   534
h1a5          APHLRPAAHDLTWYQYHSLDVIGFLLAVVLTVAFITFKCCAYGYRKCLGKKGRVKKAHKSKTH   534
zf1a10        APHLRPAAHDLNWVQYHSLDVIAFLAAITLLFLFISFKCCLCCCRCCFCKKGRTGKATKAKSH   531
h1a6          APHLRPAAHDLTWYQYHSLDVIGFLLAVVLTVAFITFKCCAYGYRKCLGKKGRVKKAHKSKTH   532
l1a6          APHLRPAAHDLNWVQYHSIDVLAFLLAASLLALFISMKCCMFCCRKCFCKSGRLTKKSKSKSH   533
l1a7          APHLRPAAHDLNWVQYHSIDVLAFLLAASLLALFISMKCCMFCCRKCFCKSGRLTKKSKSKSH   533
zf1a11        APHLRPAAHDLNWVQYHSLDVIAFLAAITLLFLFISFKCCLCCCRCCFCKKGRTGKATKAKSH   528
h1a9          APHLRPAAHDLTWYQYHSLDVIGFLLAVVLTVAFITFKCCAYGYRKCLGKKGRVKKAHKSKTH   530
h1a7          APHLRPAAHDLTWYQYHSLDVIGFLLAVVLTVAFITFKCCAYGYRKCLGKKGRVKKAHKSKTH   530
h1a10         APHLRPAAHDLTWYQYHSLDVIGFLLAVVLTVAFITFKCCAYGYRKCLGKKGRVKKAHKSKTH   530
h1a8          APHLRPAAHDLTWYQYHSLDVIGFLLAVVLTVAFITFKCCAYGYRKCLGKKGRVKKAHKSKTH   530
z1a1          AEHLRPAAHDLNWIQYHSLDVIGFLLLILLTVIFVTVKSCMFCFRKCFKKSQKKKKA------   525
z1a3          AEHLRPAAHDLNWIQYHSLDVIGFLLLILLTVIFVTVKSCMFCFRKCFKKSQKKKKA------   526
z1b1          ADHLRPAAHELNWLQYHSLDVIGFMLLIVLIVTLAMLKCCSLCWRRCCRKTQKRKED------   528
m1a1          ATHLRVAAHDLNWFQYHSLDVIGVLAIVVITVLWVTLKCCSFGVRKCCGRRSSKKKSE-----   527
s1a1          AAHLRVAAHDLNWIQYNSLDVFVFFAVVLVGVLWVTLKGCLFCGRKCFRKGTPKRKQA-----   527
f1a1          AEHLRVAAHDLNWFQYHSLDVIGFLAVVLLTVLWALLKCCSCCIRRCLRTGKKKKE-------   525
t1a1          AQHLRVAAHDLNWFQYHCLDIIGFLAVVLLTVLWVLLKCCSSCTRRCLRIGKMKKE-------   525
z1a2          AEHLRPAAHDLNWIQYHSLDVIGFLLLILLTVIFVTVKSCMFCFRKCFKKSQKKKKA------   520
z1a4          AEHLRPAAHDLNWIQYHSLDVIGFLLLILLTVIFVTVKSCMFCFRKCFKKSHKKKKA------   520
z1a5          AEHLRPAAHDLNWIQYHSLDVIGFLLLILLTVIFVTVKSCMFCFRKCFKKSHKKKKA------   519
z1a6          AEHLRPAAHDLNWIQYHSLDVIGFLLLILLTVIFVTVKSCMFCFRKCFKKSHKKKKA------   520
z1a7          AEHLRPAAHDLNWIQYHSLDVIGFLLLILLTVIFVTVKSCMFCFRKCFKKSQKKKKA------   527
z1b2          ADHLRPAAHELNWLQYHSLDVIGFMLLIVLIVTLAMLKCCSLCWRRCCRKTQKRKED------   531
z1b3          ADHLRPAAHELNWLQYHSLDVIGFMLLIVLIVTLAMLKCCSLCWRCCCRKTQKRKED------   531
z1b4          ADHLRPAAHELNWLQYHSLDVIGFMLLIVLIVTLAMLKCCSLCWRRCCRKTQKRKED------   529
z1b5          ADHLRPAAHELNWLQYHSLDVIGFMLLIVLIVTLAMLKCCSLCWRRCCRKTQKRKED------   528
z1b7          ADHLRPAAHELNWLQYHSLDVIGFMLLIVLIVTLAMLKCCSLCWRRCCRKTQKRKED------   530
f1b1          AKHLRPAVHDLNWIQYYCLDVVAFLFTILLLVVVLTVKCLKVFLRKLGGKRKRD---------   548
f1b2          AKHLRPAVHDLNWIQYYCLDVVAFLFTILLLVVVLTVKCLKVFLRKLGGKRKRD---------   548
t1b1          AKHLKPAVHELNWIQYYCLDVLAFLFTILLLVVVLTVKCLKLCFGKLGRKRKQD---------   544
s1b1          AKHLRWAGRGLNVLQYFSLDVIALLATVVLVFVILLVKCLKLFIRKVGRKRKQE---------   542
m1b1          TKHLKAAVHDLNWIQYYCLDVAALLAAIVLLFVILTVKCMKLCLQKLGKKKKQD---------   542
m1b2          AKHLKAAVHDLNWIQYYCLDVAALLAAIVLLFVILTVKCMKLCLQKLGKKKKQD---------   540
```
