# Supplementary material for: Cloning and Comparative Analyses of the Zebrafish Ugt Repertoire Reveal Its Evolutionary Diversity
Source: PLoS One. 2010 Feb 10;5(2):e9144. doi: 10.1371/journal.pone.0009144 (PMC2819257; doi:10.1371/journal.pone.0009144)
Supplement: Figure S2 — An alignment of the vertebrate Ugt2 protein sequences. The zebrafish (z), fugu (f), medaka (m), stickleback (s), xenopus (x), lizard (l), zebra finch (zf), and human (h) Ugt2 polypeptides were aligned by using the ClustalX. The amino acid residues are represented by capitalized single-letters with the degree of conservation highlighted by different colors. The Ugt2 sequence names are indicated on the left. (0.21 MB PDF) [file pone.0009144.s004.pdf]

```
                                            .::    : * * : . ::. :  : * .  :
h2a1            ------MLNN-LLLFSLQISLIGTTLGGNVLIWPMEGSHWLNVKIIIDELIKKEHNVTVLVASGALFIT---PTSNPSLTFEIYRVPFGKERIEGVIKDF   90
h2a2            ------MPKKFVQMLVFNLTLTEVVLSGNVLIWPTDGSHWLNIKIILEELIQRNHNVTVLASSATLFIN---SNPDSPVNFEVIPVSYKKSNIDSLIEHM   91
h2b28           ----MALKWTSVLLLIHLGCYFSSGSCGKVLVWTGEYSHWMNMKTILKELVQRGHEVTVLASSASILFD---PNDAFTLKLEVYPTSLTKTEFENIIMQQ   93
h2b11           ----MTLKWTSVLLLIHLSCYFSSGSCGKVLVWAAEYSHWMNMKTILKELVQRGHEVTVLASSASILFD---PNDASTLKFEVYPTSLTKTEFENIIMQQ   93
h2b10           ----MALKWT-TVLLIQLSFYFSSGSCGKVLVWAAEYSLWMNMKTILKELVQRGHEVTVLASSASILFD---PNDSSTLKLEVYPTSLTKTEFENIIMQL   92
h2b7            ----MSVKWTSVILLIQLSFCFSSGNCGKVLVWAAEYSHWMNIKTILDELIQRGHEVTVLASSASILFD---PNNSSALKIEIYPTSLTKTELENFIMQQ   93
h2b4            ----MSMKWTSALLLIQLSCYFSSGSCGKVLVWPTEFSHWMNIKTILDELVQRGHEVTVLASSASISFD---PNSPSTLKFEVYPVSLTKTEFEDIIKQL   93
h2b15           ----MSLKWTSVFLLIQLSCYFSSGSCGKVLVWPTEYSHWINMKTILEELVQRGHEVTVLTSSASTLVN---ASKSSAIKLEVYPTSLTKNYLEDSLLKI   93
h2b17           ----MSLKWMSVFLLMQLSCYFSSGSCGKVLVWPTEYSHWINMKTILEELVQRGHEVIVLTSSASILVN---ASKSSAIKLEVYPTSLTKNDLEDFFMKM   93
h2a3            ----MRSDKSALVFLLLQLFCVGCGFCGKVLVWPCDMSHWLNVKVILEELIVRGHEVTVLTHSKPSLID---YRKPSALKFEVVHMPQDRTEENEIFVDL   93
l2a1            -----MSMKWVLVVPLMMMFLLGSASSGNVLIWPADYSHWMNMKSIIHELAARGHQVTVLLPSFYQEAE---SSGPSNFHLEVISVPFSKKDMDNFMQDF   92
l2a2            -----MSMKWVLVVPLMVMLLLGSASSGNVLVWPANNSHWMNIKSIIHELTARGHQVTVLLPSFYQEAE---SSGPSNFHLEVISVPFSKKDMDNFLQDF   92
l2a5            -----MSMKLMLVVPFIMMFLLGSASTGNVLVWPYDYSHWMHMKSVIHELTARGHRVTVVMASSYQAAE---SSESSNFHVEVIPVPFTRKEMEMLTQDA   92
l2a4            ----------MLVFPIATACFVGSSSAGKVLVWPVDNSHWINMKFILNELTDRGHQVTVLLPSFFMAVK---ASEPSPFQLEEIPFLFSREDMDTMLQDF   87
zf2a1           MAMKTTSSKKYLQLLIFQVALLGPVFCGKILVWPTEGSHWLNMNVMVQELIRRGHSFTILVSNTTLFIE---PKPKTTEKFEIYNVPFDKDLPETLLNGI   97
c2a1            MATEATSFKKCLQLLAFHVALVAPVFCGNVLVWPTEGSHWLNVKMVIQELIRRGHSVTILVSNASLFIQ---PKAETTEKFEVYNVPFKKDTIANLIEDI   97
zf2a2           -----MAGKEVAVLLLLAVLGC--GSGEKVLVWPADNSHWLNMEHILQELVARGHEVTVLLPSCFLIVN---PTQPSPFQFEVIEVPITKKEMSDFMDKM   90
c2a2            -----MSGKKVAVLWLLAALGS--VYGGKVLVWPVDNSHWLNVEYILHELVARGHEVTVLLPSCFLILN---GTQPSPFQFEMIEVPISKSEMAAAMDEG   90
zf2a3           -----MLGPKFPWLLWAYTCCCSTVFCGRVVVWPTDASHWINMKVLLQELVLRGHEVTVLVPSSNLLINY--QDTSSPFTFEVLQVPFSQETLDAAMEDF   93
c2a3            -----MTGQRFSWLLWTFACCWNVGFCGKVVVWPTDASHWINMKVLLEELILRGHEVTVLVPSINMLIDY--QDTSSPFTFEVLKVNFTQETFDALMVDF   93
x2a1            -------MLWFHVSLLLQATFLCTVSSEKILAWPADASHWLNLKLILEEVASRGHEVTALIHSANMQMG---NFKSSPVKMEIFPVPFTKERNFNILNEF   90
x2a2            -------MLWIHVFLLLQLGFFSAVSSEKILAWPADASHWLNLRLILEELAHKGHEVTALVHSANMLID---HNLPSAIKAEVFPVAFTKETFLTLQRDF   90
z2b1            --------MRLVVCFLSLLTVFSAGQCGNILVWYTEGSHWINLKIVLDTLLDRGHNVTVLMPDGALFMK---AKESDRFSYQHFSVSTSAQDMQDFLDEF   89
z2b3            --------MRLVVCFLSLLTVFSAGECGNILVWHTEGSHWINLKIVLDTLLDRGHNVTVLMPDGALFMK---AKESDRFSYQNFSVSTSAQDMQDFLDEL   89
z2b5            --------MRLVVCFLSLLTVFSAGQCGNILVWYTEGSHWINLKIVLDTLLDRRHNVTVLVPDGALFMK---AKESDRFSYQHFSVSKSAQDMQDFFDEL   89
z2b6            --------MRPLACFLSLLSLLSAGECGNVLVWFTEGSHWINMKIVLETLIDRGHDVTVLVPDASLFMK---ANASDRFSYQPFNVSMDEQEMKVFIEEF   89
f2b1            ----MKPAQRLPLSVFLVLTVTLSVNGGNILVWYTEGSHWINLKPVLDTLIDRGHHVTVLVPSSSMFIN---TSESSRFRYEPFNVSISKKEMEKIMHDV   93
f2b2            ----MKPAQRLPLSVFLVFSVTLSAHGGNILVWYTEGSHWINLKPVLDTLIDRGHHVTVLVPSSSMFMN---TSEPSRFRYEPFNVSISREVMEKSMEEF   93
f2b3            ----MSTVWCLSLALLLVLSATRIAHGGNILVWYTEGSHWINLKPVLDTLIDRGHRVTVLVPSTSMFMN---RSEPSRFHYEPFDVSISLEVIEASNEEF   93
s2b4            ----MKSGQSLSACVLLLLCGTWSTDGGNILVWYTEGSHWINMKPLLETLIDRGHQVTVLVPSTSMFMS---TKEPSRFGYEPFNVSVSMETMEKFMKDF   93
s2b5            ----MKSGQSLSACVLLLLCGTWSTDGGNILVWYTEGSHWINMKPLLETLIDRGHQVTVLVPSTSMFMS---TKEPSRFSYEPFNVFVSMETMEKFMKDF   93
s2b3            ----MKSGQSLSACVLLLLCGTWSTDGGNILVWYTEGSHWINMKPLLETLIDRGHQVTVLVPSTSMFMN---TKEPSRFVYEPFNVSVS---MEKFIKDF   90
s2b1            ----MKSGQSLSACVLLLLCGTWSTDGGNILVWYTEGSHWINMKPLLETLIDRGHQVTVLVPSTSMFMN---TKEPSRFSYEPFNVSVSMETMEKFMKDF   93
s2b2            ----MKSGQSLSACVLLLLCETWSTDVGNILVWYTEGSHWINMKPLLETLIDRGHQVTVLVPSTSMFMS---TKEPSRFVYQPFNVSVSMETLEELFEEY   93
m2b1            ----MELRLSL-----LLLCLTSSASGGNILVWYTEASHWINMKPLLETLVDRGHSVTVLVPSTSMFMS---TKEPAHFHYLPFNVSVSLEDMENYLEEL   88
z2a3            -----MTSGALLVC--LLLCGVDVGWSGRVLVMPGEYSHWHNMRAIIEALVDRNHSVTVLVGSSSPTVP---HTQKARFDYHVFKVNIEKEEADAVWSDF   90
z2a4            -----MTSGVLLVC--LLLCGVDVGWSGRVLVMPGEYSHWHNMRAIVEALVDRNHSVTVLVSSSSPTVP---HTQKERFDYHLFKVNMDKEEAKVIWSDF   90
z2a5            -----MTSGALLVC--LLLCGVDVGWSGRVLVMPGEYSHWHNMRPVIEALVDKNHSVTVLVGSSSPTVP---HTQKERFDYHVFKVNMDKEEADAAMRDM   90
z2a6            -----MTSGTLLVC--LLLCGVDVGWSGRVLVMPGEYSHWHNMRPVIEALVDKNHSVTVLVGSSSPTVP---HTQKERFDYHVFKVNMEKEVVDYLWIHF   90
z2a2            -----MMKCLLKPSPLVLIFIFSFTHAGNVLVLPGEYSHWQNMRNIVDELLNRNHTVTVLVCSASPTIN---FTQQERFQYLVFEVHLSAQELDGASKEL   92
z2a1            -MSPRGSTGVFLLLALLLNQNLLCVSGGKILVWPAEFSHWLNIKVILDVLIERGHNITVVTHTATPSVQ---TTPSAGYNAEILQVPYTKQEIVDNLERM   96
f2a1            --------MKLYHLSLLLLCMSSPAFGGHVLVFPGEYSHWLNARTIMDELVRRNHSVTVLVADASPSVSYNNSRDAAKFNFLVFKVPFSRAELHGLTEEL   92
m2a1            ---MKLATVSLFSSGLLLAVFSTAAHGGKVLAFPGEYSHWLNMRTIIEELVRRNHSITVLVPDASPSVNYNNSRDAAKFDFLVFKVPFSRSDCVSMMQEF   97

                                                       .     :       :: :. *     .:: *  : :  : :   :     *   *    *
h2a1            VLTWLENRPSPSTIWRFYQEMAKVIKDFHMVSQEICDGVLKNQQLMAKLKKSKFEVLVSDPVFPCGDIVALKLGIPFMYSLRFSPASTVEKHCGKVPYPP   190
h2a2            IMLWIDHRPTPLTIWAFYKELGKLLDTFFQINIQLCDGVLKNPKLMARLQKGGFDVLVADPVTICGDLVALKLGIPFMYTLRFSPASTVERHCGKIPAPV   191
h2b28           VKRWS--DIQKDSFWLYFSQEQEILWEFHDIFRNFCKDVVSNKKVMKKLQESRFDIIFADAFFPCGELLAALLNIPFVYSLCFTPGYTIERHSGGLIFPP   191
h2b11           VKRWS--DIRKDSFWLYFSQEQEILWELYDIFRNFCKDVVSNKKVMKKLQESRFDIVFADAVFPCGELLAALLNIRFVYSLRFTPGYTIERHSGGLIFPP   191
h2b10           VKRLS--EIQKDTFWLPFSQEQEILWAINDIIRNFCKDVVSNKKLMKKLQESRFDIVFADAYLPCGELLAELFNIPFVYSHSFSPGYSFERHSGGFIFPP   190
h2b7            IKRWS--DLPKDTFWLYFSQVQEIMSIFGDITRKFCKDVVSNKKFMKKVQESRFDVIFADAIFPCSELLAELFNIPFVYSLSFSPGYTFEKHSGGFIFPP   191
h2b4            VKRWA--ELPKDTFWSYFSQVQEIMWTFNDILRKFCKDIVSNKKLMKKLQESRFDVVLADAVFPFGELLAELLKIPFVYSLRFSPGYAIEKHSGGLLFPP   191
h2b15           LDRWIY-GVSKNTFWSYFSQLQELCWEYYDYSNKLCKDAVLNKKLMMKLQESKFDVILADALNPCGELLAELFNIPFLYSLRFSVGYTFEKNGGGFLFPP   192
h2b17           FDRWTY-SISKNTFWSYFSQLQELCWEYSDYNIKLCEDAVLNKKLMRKLQESKFDVLLADAVNPCGELLAELLNIPFLYSLRFSVGYTVEKNGGGFLFPP   192
h2a3            ALNVLP-GLST---WQSVIKLNDFFVEIRGTLKMMCESFIYNQTLMKKLQETNYDVMLIDPVIPCGDLMAELLAVPFVLTLRISVGGNMERSCGKLPAPL   189
l2a1            LHVWSYE-KHEKSFWDFSITLYDFIIQFMSRYKFFCDNVFWNEKLLQKLESAAFDVLISDPVMLGGELLAEKLHVPFLYSLRFSFGNTMERICGNIPAPP   191
l2a2            IHVWSYE-KHEKPF-EFSITLYDLIVQLMSIYKLCCDSVFGNEKLLQKLESAAFDVLISDPAIVGGELLAEKLHIPFLYSLRVTFGNTMERICGNIPAPP   190
l2a5            LYLWSYE-THKIPFWDSFIKIYSHLMKMQSKYKSFCDNVFWNEKLLQKLESAAFDVLISDGVMPGGELLAEKLRVPFLYSLRLTPGNTMERICGHVPAPS   191
l2a4            LFMWSYE-SNELSFWQFFIKTHDFSREMERRNRILCDRVVLNQTLMERLESVGFDILIADPLLPCGELVAEKLHLPFVYTFRFSVGNTMECVCGVIPAPP   186
zf2a1           VDLWLNNRPTILTFWQFYKELGRLSVGWQKMNKMMCDAVLTNQELMAHLQGSGFDLLLSDAVTPCGELLALKLDIPFVYSLRFSPAFTLERHCGKIPAPP   197
c2a1            MTLWLNNRPTASTFWQFYKELGKLSKSWHQVNRQMCDAVLTNQELMAHLQGSGYDLLLSDPVTPCGDLVALKLALPFVYSLRFSPASTVERHCGKIPAPP   197
zf2a2           FYFFFNE-DRKIPIWKSTYKIAQMVLQMKNITKIICDGVVKNEALMERLRASAFNVLLADPLFPSGELVAEKLGIPFVYTFRFSMGNTVERICGTLPAPP   189
c2a2            FYFWFYE-ERRLPFWESLYKMIDLLRKFENITRTICDAVLKDKVLLERLRTSAFDVLLADPLMPSGELFAEKLGIPLVYTIRFSMGNTVERLCGGLPAPP   189
zf2a3           LNFWMNK-ASNLFPWEIMWTMKEQLEVFTNMSKQTCDTLVMNSHLIAKLQRAKFDVLIADPLSVGGELVAEILEIPFVYSFRFSDGNVVERRCGGLLSPP   192
c2a3            LDLWIND-LPNLFPWEVMWRMKEAIYSFSNLSKHSCDALVSNHQLIAKLHQAKFDVLIADPLAVCGELAAQLLEIPFVYSFRFSEGNVVERLCGGLPSPP   192
x2a1            IQLWVYD-TPYISYWEFYVRTKELLKDAVELQMYTCDGVVKNKDLLYKLQNEKFDVVVADPLVPCGELIAEILGVPFVYTFRFSMGNSVERLCGQLPSPF   189
x2a2            FNFWIYG-IHNMSYWELYEHLKVAVASSLKLQRDTCDGVLKNKELMYKLQNQKFDIVVADPVAPCGELIAESLGVPFVYSFRFSMGNSLERLCGQLPSPF   189
z2b1            LHFSVFE-LENSNLLQIQMKFFDLLSKHQDMSLSYCDGILKSPELMDKLKKGKFDVLLSDPMYPCSDIVAEELNVPLVYTFRFSLAHTMERMCGQIPAPP   188
z2b3            LHFSVFE-MEQLNLLQIQMKFFDLGSRHQDMGLSYCDGILKSPELMDKLKTGKFDVLLSDPMYPCSDIVAEELNVPLVFTLRFSIAHTMERMCGQIPAPP   188
z2b5            MRFSVFE-MDNSSFLEIQMKFFNLGSRHQDMSLSYCDGILKSPELMDKLKKGKFDVVLSDPMYPCSDIVAEELNVPLVYTFRFSVAHAAERMCGQIPAPP   188
z2b6            LYFSLYE-MDQLNFFQMQSKVYEFTSKLQDMSISYCDGVLKSPGLLDKLRKHKFDVVFSDPIYQCSDIVAEELNVPLVYTFRFSLAHAAERMCGQIPAPP   188
f2b1            LQFLIYD-MDHMNYWEIFLKFLETTKVNLECSLKLLDGVVKSETMMKRLREGNYDLLLSDPIYSGSELLAEMLDLPLVFSLRFSLANNWERHCGQIPAPP   192
f2b2            LHFSIYE-MDHMSYWEIYNKFQAMVHINLQNNLKCLDGVVKSETMMKRLREGNYDLLLSDPLYPGSDLLAEMLDLPLVFSLRFSLANNWERHCGQIPAPP   192
f2b3            LQFSMYE-IDHMNYIEMYIKFIDLAMIELRNNLKYLDGVVKSETIMKRLKEAKYDLLLADPLYPGSELLADLLNLPLVFSLRFSPALNWERHCGQIPAPP   192
s2b4            LQFSMYE-IDHMSYVQIYIRFMDMMEDNLNKMFIYLDGVVKSEPIMKKLKDRKYDLLLADPVYRGGDLVADILGIPLIFSLRFSLAHHWERQCGQLPAPP   192
s2b5            LQFSMYE-IDHMSYVQIYIRFMDMMEDNLNKMFIYLDGVVKSEPIMKKLKDGKYDLLLADPVYRGGDLVADILGIPLVFSLRFSLAHHWERQCGQLPAPP   192
s2b3            IHFSMYE-IDHLSYMQIYIRFIDMIKDNLKHTLICIDGVVKSEPIMKKLKEGKYDLLLADPVYPGGDLVADILGIPLVFSLRFSLAHHWERQCGQLPAPP   189
s2b1            LHFSMYE-MDHMSYVQIYIRFMDMMKDNLKYSLMYLDGVVKSEPIMKKLKEAKYDLLLSDPVYPGSDLVADILGIPLVFSLRFSFANNWERQCGQLPAPP   192
s2b2            LHFSMYE-IDHMSYVQMYIRYMDLMSQDLQNALKYVDGVVKSEPIMKKLKEGKYDLLLADPIFHGGDLVADILGIPLVLSVRFSLAHHWERQCGQLPAPP   192
m2b1            LHFSMYE-MDYMNYFQIYKKFIDLMEFEMSYSLDYLDGVVKSETTMKKLKEGHFDLLLSDPIYPGSDLVADILGLPLVLSLRFSMVNNWERYCGQLPSPP   187
z2a3            IYLWMNET-ATN--YETVSNIFQVMSRFMTLTDEVCKGMF-NEDLLQMLRESHYDVLFSDPMMPCSDLMAQTLNIPLVLSLRATFAYSFERMCGQMPAPP   186
z2a4            THLWMKET-DSK--LERGFLIWRVMSNFRRLAADVCRSLLQNDDLLQMLGESHYDVLFSDPMMPCSDLMAQTLNIPQVISLRLTFAYTFERMCGQMPAPP   187
z2a5            IHLFTNDT-AAK--YEGEFMMWKVMSKFVEFGDVYFRSLF-DVGLLKTLRDSNYDVLFSDPMMPFSDLMAQKLNIPQVISMRATFAYAAERLCGQMPAPP   186
z2a6            HELWMNET-IST--YEKTLEVWQLMSLFRTHSEELVKGLF-DVGLLKTLRDSNYDVLFSDLTMPFSDLMAQKLNIPHVLSMRISFASALERLCGLMPVPP   186
z2a2            IHIWTQHP-SPNR-LQTALQIIELLGRVRVMHRAMCDSMLRNDALIAQLSALKFDVLFNDPTFMCADLLAEMLGLPLVLSVRISPGFCLERMCGQMPAPP   190
z2a1            LKYWTHDLPNDNI-ITASLKIHEMITTATAQNKITCNAPFSRADLLEKWKMEKFDVILADPLYICGEILAQKLDIPLIFSLRFTFGNTLERLCGQMPAPP   195
f2a1            VHFAMYE-YPTASFLEKVWKIYDLLRRFASFGLKQCDAILKNQQLMATLRDAAFDAVLLDPMIMCGDLVADVLGLPLVISLRFSQGGVMERHCGHVPAPP   191
m2a1            MDFSMYE-MHSSSLLQVFWRMSYFMQRSSDLGMQQCDAMLKNQQLMATLRDASYDVVLVDPLVMCGDLVADLLGVPLILSLRFSFGSTLERHCGHAPFPP   196
```

```
        *. *         . * * :*  .                        .  ::.  * .:.     .  ..:: *:*. *.:. * *  **...:**:** **  *.
h2a1    SYVP--AVLSELTDQMSFTDRIRNFISYHLQDYMFETL-WKSWDSYYSKALGRPTTLCETMGKAEIWLIRTYWDFEFPRPYLPNFEFVGGLHCKPAKPLP   287
h2a2    SYVP--AALSELTDQMTFGERIKNTISYSLQDYIFQSY-WGEWNSYYSKILGRPTTLCETMGKAEIWLIRTYWDFEFPRPYLPNFEFVGGLHCKPAKPLP   288
h2b28   SYIP--VVMSKLSDQMTFMERVKNMIYVLYFDFWFQMCDMKKWDQFYSEVLGRPTTLFETMGKADIWLMRNSWSFQFPHPFLPNIDFVGGLHCKPAKPLP   289
h2b11   SYIP--IVMSKLSDQMTFMERVKNMIYVLYFDFWFQMSDMKKWDQFYSEVLGRPTTLFETMGKADIWLMRNSWSFQFPHPFLPNVDFVGGFHCKPAKPLP   289
h2b10   SYVP--VVMSKLSDQMTFMERVKNMLYVLYFDFWFQIFNMKKWDQFYSEVLGRPTTLSETMRKADIWLMRNSWNFKFPHPFLPNVDFVGGLHCKPAKPLP   288
h2b7    SYVP--VVMSELTDQMTFMERVKNMIYVLYFDFWFEIFDMKKWDQFYSEVLGRPTTLSETMGKADVWLIRNSWNFQFPYPLLPNVDFVGGLHCKPAKPLP   289
h2b4    SYVP--VVMSELSDQMTFIERVKNMIYVLYFEFWFQIFDMKKWDQFYSEVLGRPTTLSETMAKADIWLIRNYWDFQFPHPLLPNVEFVGGLHCKPAKPLP   289
h2b15   SYVP--VVMSELSDQMIFMERIKNMIHMLYFDFWFQIYDLKKWDQFYSEVLGRPTTLFETMGKAEMWLIRTYWDFEFPRPFLPNVDFVGGLHCKPAKPLP   290
h2b17   SYVP--VVMSELSDQMIFMERIKNMIYMLYFDFWFQAYDLKKWDQFYSEVLGRPTTLFETMGKAEMWLIRTYWDFEFPRPFLPNVDFVGGLHCKPAKPLP   290
h2a3    SYVP--VPMTGLTDRMTFLERVKNSMLSVLFHFWIQDYDYHFWEEFYSKALGRPTTLCETVGKAEIWLIRTYWDFEFPQPYQPNFEFVGGLHCKPAKALP   287
l2a1    SYVP--ASMGEQTDRMSFMERIANMLTYLSHDIVFHHLIFKEWNKYYSDVLGRPTTLCETMGKAEIWLIRTYWDFEFPRPLLPNFEFVGGLHCQPAKPLP   289
l2a2    SYVP--ASMGELTDRMSFMERMANMLTYFSHDIMFHRLIYKEWNKYYSNVLGRPTTLCETMGKAEIWLIRTYWDFEFPRPLLPNFEFVGGLHCQPAKPLP   288
l2a5    SYVP--TGLVGLTDRMSFMERTANMLTYLSLDIMFHHFFNKDWDQYFSDVLGKPTTLCEIMGKAEIWLIRTYWDFEFPRPLLPNFEYVGGLHCQPAKPLP   289
l2a4    SFVP--ASLGGLTDRMSFMQRTRNMLFYALHDILFHQVLWKDWSKYYSDVLGRPTTLCETMGKAEIWLIRTYWDFEFPRPLLPNFEFVGGLHCQPAKPLP   284
zf2a1   SYTP--AALSELTDRMSFGERVKNFVSYHLQDYVFRSYWG-HWDNYYSKVLGRPTTLCETMGKAEIWLIRTYWDFEFPRPFLPNFEFVGGLHCQPAKPLP   294
c2a1    SYTP--AALSELTDHMSFGERIKNILSYHLQDYIFQSYWG-EWDIYYSKILGRPTTLCETMGKAEIWLIRTYWDFEFPRPFLPNFEFVGGLHCQPAKPLP   294
zf2a2   SYVP--TTLTSLTDRMTFWQRLKNILGYALHDFVFHYVLWTSWDQYYSEVLGRPTTLCETMGKAEIWLIRTYWDFEFPRPFLPNFEFVGGLHCQPAKPLP   287
c2a2    SYVP--ASLSSLTDKMTFTERLKNMFSYALQDLAYHYVLWGHWNQFYSDILGRPTTLCETMGKAEIWLIRTYWDFEFPRPFLPNFEFVGGLHCQPAKPLP   287
zf2a3   SYVP--ASTMGLTHQMSFMERLQNFLFYIFTDLFFMKFWRDEWDGYYSNVLGRPTTLCETMGKAEIWLIRTYWDFEFPRPFLPNFEFVGGLHCQPAKPLP   290
c2a3    SYVP--ASTTGLTDQMSFVERLWNFFFYLSMDLFFLKFWRDEWDGYYSNVLGRPTTLCETMGKAEIWLIRTYWDFEFPRPFLPNFEFVGGLHCQPAKPLP   290
x2a1    SYVP--GTMAELIDRMSFGERLVNTIHYLFQDLLFALLFQWHWDEYYSEVLGRPAKVCEIMGKAEIWLIRTYWDFEYPRPILPNFKFVGGLHCAPAKPLP   287
x2a2    SYVP--GAMTALSDKMSFGQRLANTVLYAFQDLIFSLVFQKNWDNYYSEILGRPAKVCEIMGKAEIWLIRTYWDFEYPRPILPNFKFVGGLHCAPAKPLP   287
z2b1    SHVP--GATSKLTDKMSFTERICSMLFYLSID-TFYRLIWKRFDNYYTEYLGRPTSYCEMMGRADIWLIRTYWDFEFPRPFVPNFKYIGGLHCTPAKPLP   285
z2b3    SYVP--GAMSKLTDKMSFTERIFNTLFYLSMD-TFSVIAWKKFDNYYTELFGRPTSYCEMMGRADIWLIRTYWDFEFPRPFVPNFKYIGGLHCTPAKPLP   285
z2b5    SYVP--GAMSKLTDKMSFTERIFNMLFYLSQD-TLSRLIWRRFDNYYTEYFGRSTSYCEMMGKADIWLIRTYWDFEFPRPFVPNFKYIGGLHCTPAKPLP   285
z2b6    SYVP--GAMSKLTDKMSFTERIYNMLFYLSQD-AFAIFAWKKIDNYYTEYFGRPTSYCEMMGRADIWLIRTYWDFEFPRPFVPNFKYIGGLHCTPAKPLP   285
f2b1    SFVP--IAMSRMTDKMNFFERVLNFYLYALQDIVIENTFWKDLDAYYSEVKGTPTSACKMMSNVDIWLMRTYWDFDFPRPFLPNFKFVGGIHCRPAKPLP   290
f2b2    SFVP--IAMSKMTDKMNFFERVLNFFLYALQDIVIENTLWKDLDAYYSEVKGTPTSACKVMSNTDIWLMRTYWDFDFPRPFLPNFKFVGGIHCRPAKPLP   290
f2b3    SFVP--GAMSKLTDTMDFSERLWNFLFYAVQDIVMQFTYWKEVDNYYSEFKGEPTSSCELMSKADIWLMRTYWDFDFPHPYLPNIKFVGGIHCRPAKPLP   290
s2b4    SFVP--GAMSKLTDRMDFSERVWNFLFYALQDIVIDHVSWNVLDKYYSEVKGTPTSACETMGRADIWLMRNYWDFDFPRPSLPNFKFVGGIHCRPAKPLP   290
s2b5    SFVP--GAMSKLTDRMDFSERVWNFLFYALQDFVIDHVHWKVLDKYYSEVKGTPTSACETMGRADIWLMRSYWDFDFPRPSLPNFKFVGGIHCRPAKPLP   290
s2b3    SFVP--GAMSKLTDRMDFSERVWNFLFYALQDIVINHVSWKVLDKYYSEVKGTPTSACETMGRADIWLMRSYWDFDFPRPSLPNFKFVGGIHCRPAKPLP   287
s2b1    SFVP--GAMSKLTDRMDFSERVWNFLFYALQDILLENIFWKELDKYYSQVKGTPTSACETMGRADIWLMRTYWDFDFPRPSLPNFKFVGGIHCRPAKPLP   290
s2b2    SFVP--GAMSKLTDRMDFSERVWNFLFYALQDIVIDHVNWKVLDKYYSEVKGTPTSACETMGRADIWLMRTYWDFDFPRPSLPNFKFVGGIHCRPAKPLP   290
m2b1    SFVP--ASMSKLTDKMDFFDRVWNFLFYALNDVVLEPIYWTRVDKYYSEVRGMPTNGCQLMGRADIWLIRTYWDFEFPRPFLPNFKFVGGIHCRPAKPLP   285
z2a3    SYVPAASLRDYLTDRMSFMERVENMLLYFSHDVFFKLYMMFTFDRIYSEIRGKPTTMCETMGKADIWLIRTYWDFEYPRPFPPNFKFVGGLHCKPAKPLS   286
z2a4    SYVPAVALSDHLTDRMSFMERVENMLLYFIHTTVFRLNTMLTVDRLYTEIRGKPTTMCETMGKADIWLIRTYWDFEYPRPFPPNFKFVGGLHCKPAKPLS   287
z2a5    SYVPAVALTDHLTDRMSFMERVENMLLYIVHSIAFPLVATFTFDGYYSEILGKPTTMCETMGKVDICLIRTYWDFEYPRPFPPNFKFVGGLHCKPAKPLS   286
z2a6    SYVPAAVAQGHLTDRMSFTERVENMLLYITHTAMFQLTTKFTFDHIYAEISGEPTTMCETIGKTDIWLIRTYWDFEYPRPFPPNFKFVGGLHCKPAKPLA   286
z2a2    SFVP--ITQTVLTDRMCFMERVQNMIANIVFSVSFYMVAWISLDSYYTDVLGKPTTMCETMGKADIWLIRTYWDFEYPRPFPPNFKFVGGLHCKPAKPLS   288
z2a1    SYVP--AAASEKTDQMDFIDRLKNFLFYGMQDFLFYLVTKFKWDHYYSEVLGKPTTMCETMGKADIWLIRTYWDFEYPRPFPPNFKFVGGLHCKPAKPLS   293
f2a1    SFVP--PPPLPYSDLMTFTERLTSVMTYVSASAISELFWRWSLDHYYSQIKGRPSSACETLGKADIWLMRTFWDMEPPRPLMPNFKHVGGLHCKPAGPLP   289
m2a1    SFVP--TAPLPYSDHMSFVQRLTNTLMTVSMSVLHEALWRLKLDPYYTEVKGSPSSYCGTFGKADIWLIRTFWDLETPRPSPPNFKFVGGLHCKPANQLP   294

        :::* . :***. *:::.::*:.. . . .  : :* *:.:: *:*:**: *  * :*. *:   .*:******. . ::.*::*** **::**:.* :*
h2a1    KEMEEFIQSSGKNGVVVFSLGSMVKNLTEEKANLIASALAQIPQKVLWRYKGKKPATLGNNTQLFDWIPQNDLLGHPKTKAFITHGGTNGIYEAIYHGVP   387
h2a2    KEMEEFIQSSGKNGVVVFSLGSMVKNLTEEKANLIASALAQIPQKVLWRYKGKKPATLGNNTQLFDWIPQNDLLGHPKTKAFITHGGTNGIYEAIYHGVP   388
h2b28   KEMEEFVQSSGENGVVVFSLGSVISNMTAERANVIATALAKIPQKVLWRFDGNKPDALGLNTRLYKWIPQNDLLGLPKTRAFITHGGANGIYEAIYHGIP   389
h2b11   KEMEEFVQSSGENGVVVFSLGSVISNMTAERANVIATALAKIPQKVLWRFDGNKPDALGLNTRLYKWIPQNDLLGHPKTRAFITHGGANGIYEAIYHGIP   389
h2b10   KEMEEFVQSSGENGVVVFSLGSMVSNMTEERANVIATALAKIPQKVLWRFDGNKPDALGLNTRLYKWIPQNDLLGHPKTRAFITHGGANGIYEAIYHGIP   388
h2b7    KEMEDFVQSSGENGVVVFSLGSMVSNMTEERANVIASALAQIPQKVLWRFDGNKPDTLGLNTRLYKWIPQNDLLGHPKTRAFITHGGANGIYEAIYHGIP   389
h2b4    KEMEEFVQSSGENGVVVFSLGSMVSNTSEERANVIASALAKIPQKVLWRFDGNKPDTLGLNTRLYKWIPQNDLLGHPKTRAFITHGGANGIYEAIYHGIP   389
h2b15   KEMEEFVQSSGENGIVVFSLGSMISNMSEESANMIASALAQIPQKVLWRFDGKKPNTLGSNTRLYKWLPQNDLLGHPKTKAFITHGGTNGIYEAIYHGIP   390
h2b17   KEMEEFVQSSGENGIVVFSLGSMISNMSEESANMIASALAQIPQKVLWRFDGKKPNTLGSNTRLYKWLPQNDLLGHPKTKAFITHGGTNGIYEAIYHGIP   390
h2a3    KEMENFVQSSGEDGIVVFSLGSLFQNVTEEKANIIASALAQIPQKVLWRYKGKKPSTLGANTRLYDWIPQNDLLGHPKTKAFITHGGMNGIYEAIYHGVP   387
l2a1    KEMEEFVKSSGEHGIVVFSLGSMIKNLTDDKSNMVALALSQLPQKVIWRYQGKRPETLGANTRLYDWIPQNDLLGHPKTKAFITHGGTNGIYEAIYHGIP   389
l2a2    KEMEEFVKSSGEHGIVVFSLGSMIKNLTDDKSNMVALALSQLPQKVIWRYQGKRPETLGANTRLYDWIPQNDLLGHPKTKAFITHGGTNGIYEAIYHGIP   388
l2a5    KEMEEFVQSSGEHGIVVFSLGTMIQNLTDEKGNLVALAFSRLPQKVVWRYKGKRPETLGTNTRIYDWIPQNDLLGHPKTKAFITHGGTNGIYEAIYHGIP   389
l2a4    KEMEEFVQSSGEHGIVVFSLGSMLKNLTDEKSNLVALALSQLPQKVIWRYKGKRPETLGANTRLYDWIPQNDLLGHPKTKAFITHGGTNGIYEAIYHGIP   384
zf2a1   KEMEEFVQSSGEHGVIIFTLGSMVHSLSDEKSNVIAKALSQLPQKVLWRYKGKKPETLGSNTRIFDWIPQNDLLGHPLTKAFITHGGTNGLYEAIYHGIP   394
c2a1    KEIEEFVQSSGEHGIVVFSLGSMVYNLTSERSNVIARALSQLPQNVLWRYKGKKPEALGSNTRIYDWIPQNDLLGHPLAKAFITHGGTNGIYEAIYHGIP   394
zf2a2   KEMEEFVQSSGEHGVIIFTLGSMVHSLSDEKSNVIAKALSQLPQKVLWRYKGKKPETLGSNTRIFDWIPQNDLLGHPLTKAFITHGGTNGLYEAIYHGIP   387
c2a2    KEIEEFVQSSGEHGIVVFSLGSMVYNLTSERSNVIARALSQLPQNVLWRYKGKKPEALGSNTRIYDWIPQNDLLGHPLAKAFITHGGTNGIYEAIYHGIP   387
zf2a3   KEMEEFVQSSGEHGVIIFTLGSMVHSLSDEKSNVIAKALSQLPQKVLWRYKGKKPETLGSNTRIFDWIPQNDLLGHPLTKAFITHGGTNGLYEAIYHGIP   390
c2a3    KEIEEFVQSSGEHGIVVFSLGSMVYNLTSERSNVIARALSQLPQNVLWRYKGKKPEALGSNTRIYDWIPQNDLLGHPLAKAFITHGGTNGIYEAIYHGIP   390
x2a1    EEIEKVVESSGEHGIVVFSMGSMIKNFSDERTNIIAAALSQLPQKVLWRYSGKKPDALGENTITYDWIPQNDLLGHPKTKAFITHGGTNGIYEAIYHRVP   387
x2a2    EEIEKVVESSGEHGIVVFSMGSMIKNFSDERTNIIAAALSQLPQKVLWRYSGKKPDALGENTITYDWIPQNDLLGHPKTKAFITHGGTNGIYEAIYHRVP   387
z2b1    KDMEEFVQSSGDDGIVVFTLGSMIDKVPKEMSNRIASALAQIPQKVLWRYGGEKPDTLGENTRIYKWMPQNDLLGHPKTRAFITHGGTNGIYEAIYHGVP   385
z2b3    KDMEEFVQSSGDDGIVVFTLGSMIDKVPKEMSNRIASALAQIPQKVLWRYGGEKPDTLGENTRIYKWMPQNDLLGHPKTRAFITHGGTNGIYEAIYHGVP   385
z2b5    KDMEEFVQSSGDDGIVVFTLGSMIDKVPKEMSNRIASALAQIPQKVLWRYGGEKPDTLGENTRIYKWMPQNDLLGHPKTRAFITHGGTNGVYEAIYHGVP   385
z2b6    KDMEEFVQSSGDDGIVVFTLGSLVGKVPKEISNRIASALAQIPQKVLWRYGGEKPDTLGENTRIYKWIPQNDLLGHPKTRAFITHGGTNGIYEAIYHGVP   385
f2b1    EDMEEFVQSSGDAGVVIFTLGSMVKNITREKGNTIASALAQIPQKVLWRFSGQKPETLGSNTRIYDWIPQNDLLGHPKTRAFVTHGGTNGIYEAIYHGVP   390
f2b2    EDMEEFAQSSGDAGIVVFTLGSMIKNITKEKSNMIASALAQIPQKVLWRYSGEKPDTLGANTRLFDWMPQNDLLGHPKTRAFITHGGTNGIYEAIYHGVP   390
f2b3    KDMEEFAQSSGDAGIVVFSLGSFINNITTEKANMVASALAQIPQKVLWRYSGKVPDTLGANTRIYQWIPQNDLLAHPKTRAFITHGGTNGIYEAIYHGVP   390
s2b4    EDMEEFVQSSGDAGIVVFSLGSLISNITTEKANIIASALAQIPQKVLWRYSGEAPATLGVNTRIYNWIPQNDLLGHPKTRAFITHGGANGIFEAIYHGVP   390
s2b5    EDMEEFVQSSGDAGIVVFSLGSLISNITTEKANIIASALAQIPQKVLWRYSGEAPATLGANSRIYNWIPQNDLLGHPKTQAFITHGGANGIFEAIYHGVP   390
s2b3    EDMEEFVQSSGDAGIVVFSLGSLISNITTEKANMIASALAQIPQKVLWRYSGEAPATLGVNTRIYNWIPQNDLLGHPKTRVFITHGGANGIFEAIYHGVP   387
s2b1    EDVEEFVQSSGDAGIVVFTLGSFIKNITAEKANMIASALAQIPQKVLWRYSGDKPATLGGNTRLYDWIPQNDLLGHPKTRAFITHGGTNGIYEAIYHGVP   390
s2b2    EDVEEFVQSSGDAGIVVFSLGSFIKNITAEKANMIASALAQIPQKVLWRYSGDKPATLGGNTRLYDWIPQNDLLGHPKTRAFITHGGTNGIYEAIYHGVP   390
m2b1    KDMEEFVQSSGDAGIVVFTLGSMIKNITTSKANMIASALAQIPQKVVWRYSGEKPVTLGPNTKLYDWIPQNDLLGHPKTRAFITHGGTNGLYEAIYHGVP   385
z2a3    KELEEFVQSSGDHGVVVFSLGSMIKNLTSERANTIAAALGQIPQKVVWRYSGKTPETLAPNTKIYDWIPQNDLLGHPKTKAFIAHGGTNGLYEAIYHGVP   386
z2a4    KELEEFVQSSGDHGVVVFSLGSMIKNLTSERANTIAAALGQIPQKVVWRYSGKTPETLAPNTKIYDWIPQNDLLGHPKTKAFIAHGGTNGLYEAIYHGVP   387
z2a5    KELEEFVQSSGDHGVVVFSLGSMIKNLTSERANTIAAALGQIPQKVVWRYSGKTPETLAPNTKIYDWIPQNDLLGHPKTKAFITHGGTNGLYEAIYHGVP   386
z2a6    KEMEEFVQSSGDHGIVVFSLGSMIKNLTVQKANTIAAALGQISQKVVWRYSGKTPEALAPNTKIYDWIPQNDLLGHPKTKAFITHGGTNGLYEAIYHGVP   386
z2a2    KELEEFVQSSGDHGVVVFSLGSMIKNLTSERANTIAAALGQIPQKVVWRYSGKTPETLAPNTKIYDWIPQNDLLGHPKTKAFIAHGGTNGLYEAIYHGVP   388
z2a1    KELEEFVQSSGDHGVVVFSLGSMIKNLTSERANTIAAALGQIPQKVVWRYSGKTPETLAPNTKIYDWIPQNDLLGHPKTKAFIAHGGTNGLYEAIYHGVP   393
f2a1    EDVEAFVQSSGEAGVIVVSFGSMVTDLPTERAEVMAAAFGRMRQKVIWRYSGAAPQTLSANTKLMKWIPQNDLLGHTQTRVFVTHGGTNGLYEAIHHAVP   389
m2a1    EEMEAFVQSSGNAGVVVVTFGSMVTNLTAERAEVIASALGRIPQKVIWRYCGHRPATLAPNTKLYDWIPQNDLLGHSRTRAFVTHGGTNGLYEALFHGVP   394
```

```
             ::* *:* :* ::: ::   . *  :. :  :     :    :. :: .  :: .  :** :::::*  .    :*:*:*:.* : ******  ::.* * *:
h2a1         MVGVPMFADQPDNIAHMKAKGAAVEVN-LNTMTSVDLLSALRTVINEPSYKENAMRLSRIHHDQPVKPLDRAVFWIEFVMRHKGAKHLRVAAHDLTWFQY   486
h2a2         MVGVPMFADQPDNIAHMKAKGAAVEVN-LNTMTSVDLLSALRTVINEPSYKENAMRLSRIHHDQPVKPLDRAVFWIEFVMRHKGAKHLRVAAHDLTWFQY   487
h2b28        MVGIPLFWDQPDNIAHMKAKGAAVRLD-FHTMSSTDLLNALKTVINDPSYKENVMKLSIIQHDQPVKPLHRAVFWIEFVMCHKGAKHLRVAARDLTWFQY   488
h2b11        MVGIPLFFDQPDNIAHMKAKGAAVRLD-FNTMSSTDLLNALKTVINDPLYKENIMKLSRIQHDQPVKPLDRAVFWIEFVMPHKGAKHLRVAAHDLTWFQY   488
h2b10        MVGIPLFFDQPDNIAHMKAKGAAVRVD-FNTMSSTDLLNALKTVINDPSYKENIMKLSRIQHDQPVKPLDRAVFWIEFVMRHKGAKHLRVAAHNLTWFQY   487
h2b7         MVGIPLFADQPDNIAHMKARGAAVRVD-FNTMSSTDLLNALKRVINDPSYKENVMKLSRIQHDQPVKPLDRAVFWIEFVMRHKGAKHLRVAAHDLTWFQY   488
h2b4         MVGVPLFADQPDNIAHMKAKGAAVSLD-FHTMSSTDLLNALKTVINDPLYKENAMKLSRIHHDQPVKPLDRAVFWIEFVMRHKGAKHLRVAAHDLTWFQY   488
h2b15        MVGIPLFADQHDNIAHMKAKGAALSVD-IRTMSSRDLLNALKSVINDPVYKENVMKLSRIHHDQPMKPLDRAVFWIEFVMRHKGAKHLRVAAHNLTWIQY   489
h2b17        MVGIPLFADQHDNIAHMKAKGAALSVD-IRTMSSRDLLNALKSVINDPIYKENIMKLSRIHHDQPVKPLDRAVFWIEFVMRHKGAKHLRVAAHNLTWIQY   489
h2a3         MVGVPIFGDQLDNIAHMKAKGAAVEIN-FKTMTSEDLLRALRTVITDSSYKENAMRLSRIHHDQPVKPLDRAVFWIEFVMRHKGAKHLRSAAHDLTWFQH   486
l2a1         MVGTPMFGDQPDNIAHMRAKGMAVELN-IHTMTAQDLVDAVNTVIHNATYKENAVRLSKIHHDQPMKPLDRAVFWIEFVMRHKGAKHLRVAAHDLTWYQY   488
l2a2         MVGTPMFGDQPDNIAHMRAKGMAVELN-IHTMTAQDLVDAVNTVIHNATYKENAVRLSKIHHDQPMKPLDRAVFWIEFVMRHKGAKHLRVAAHDLTWYQY   487
l2a5         MIGIPMFGDQSDNIAHVCAKGMAIELN-INTMTAQDLVDAVNTIIHNTTYKENAVRLSQIHHDQPMKPLDRAVFWIEFVMRHKGAKHLRVAAHDLTWYQY   488
l2a4         MVGIPMFADQPDNIAHMRAKGMAVELN-MYTMTAQDLLDAVNTVIHNTTYKENAVRLSKIHHDQPMKPLDRAVFWIEFVMRHKGAKHLRVAAHDLTWYQY   483
zf2a1        MVGIPMFADQHDNLVHMVAKGAAVQVD-FNTMKTQDLVDALNTVIYNSTYKENALKLSKIQHDQPVKPLDRAVFWIEFVMRHKGAKHLRPAAHHLTWYQY   493
c2a1         MVGIPMFADQHDNVAHMRAKGAAVELD-FSTLTTQNLVDAVNTVINNSTYKESVLKLSKIHHDQPIKPLDRAVFWIEFVMRHKGAKHLRPAAHHLTWYQY   493
zf2a2        MVGIPMFADQHDNLVHMVAKGAAVQVD-FNTMKTQDLVDALNTVIYNSTYKENALKLSKIQHDQPVKPLDRAVFWIEFVMRHKGAKHLRPAAHHLTWYQY   486
c2a2         MVGIPMFADQHDNVAHMRAKGAAVELD-FSTLTTQNLVDAVNTVINNSTYKESVLKLSKIHHDQPIKPLDRAVFWIEFVMRHKGAKHLRPAAHHLTWYQY   486
zf2a3        MVGIPMFADQHDNLVHMVAKGAAVQVD-FNTMKTQDLVDALNTVIYNSTYKENALKLSKIQHDQPVKPLDRAVFWIEFVMRHKGAKHLRPAAHHLTWYQY   489
c2a3         MVGIPMFADQHDNVAHMRAKGAAVELD-FSTLTTQNLVDAVNTVINNSTYKESVLKLSKIHHDQPIKPLDRAVFWIEFVMRHKGAKHLRPAAHHLTWYQY   489
x2a1         IVGIPLFAEQPDNIVHMKSKGMAIMLD-FNKMGTQDLLDAVNTVINNPTYKENAMRLSRIHHDQPVKPLDSAVFWIEFVMRHKGAKHLRPASHELTWYQY   486
x2a2         IVGIPLFAEQPDNIVHMKSKGMAIMLD-FNKMGTQDLLDAVNTVINNPTYKENAMRLSRIHHDQPVKPLDSAVFWIEFVMRHKGAKHLRPASHELTWYQY   486
z2b1         MVGIPLFGDQPDNMVHMTTRGAAVVVDSIKSMQPQELVDKLNTVINDPSYKENAMRLSRIHHDRPMKPLDESVFWIEFVMRNKGAKHLRVEAHNLTWYQY   485
z2b3         MVGIPLFGDQPDNMVHMTTRGAAVVVDSIKSMQPQELVDKLNTVINDPSYKENAMRLSRIHHDRPMKPLDESVFWIEFVMRNKGAKHLRVEAHNLTWYQY   485
z2b5         MVGIPLFGDQPDDMVHMATRAAAVVVDSIKSMQPQELVDKLNTVINDPSYKENAMRLSRIHHDRPMKPLDESVFWIEFVMRNKGAKHLRVEAHNLTWYQY   485
z2b6         MVGIPLFGDQPDNMVHMTTRGAAVVVDSIKSMQPQELVDKLNTVINDPSYKENAMRLSRIHHDRPMKPLDESVFWIEFVMRNKGAKHLRVEAHNLIWYQY   485
f2b1         MVGIPLFGDQPENLVHMKAKGAAVDVN-FKTMTTEGLRDAINAVINNKLFKENAMRLSKIHHDRPLSARDEAVFWIEFTMRNKGAKHLRVHSHDLTWYQY   489
f2b2         MVGIPMFGDQPENLVHMMAKGVATTVD-FTTMTTEGLRDAINDVINNKSYKENAMRLSKIHHDRPLSARDEAVFWIEFTMRNKGAKHLRVHSHDLTWYQY   489
f2b3         MVGIPMFADQPDNLVHMMAKGAAVMVN-LNFINAEGLRDAINTVINDKSYKENAMRLSKIHHDRPMSPRDEAVFWIEFTMRNKGAKHLRVQAHELTWYQY   489
s2b4         MVGIPMFADQPDNMVHVEAKGAAVIVN-LNFMKQDDLRDAVNTVINDKSYKENALRLSRIHHDRPMSPLDEAVFWMEFTMRNKGAKHLRVQAHELTWYQY   489
s2b5         MVGIPMFFDQPDNMVHVEAKGAAVIVN-LNFMKQDDLRDAVNTVINDKSYKENALRLSRIHHDRPMSPLDEAVFWMEFTMRNKGAKHLRVQAHELTWYQY   489
s2b3         MVGIPMFADQPDNMVHVEAKGAAVTVS-LNFMKQDDLRDAVNTVINDKSYKENALRLSRIHHDRPMSPLDEAVFWMEFTMRNKGAKHLRVQAHELTWYQY   486
s2b1         MVGIPMFADQPDNMVHVEAKGAAVTVN-LNFMKKDDLRDAVNTVINDKSYKENALRLSGIHHDRPMSPLDEAVFWMEFTMRNKGAKHLRVQAHELTWYQY   489
s2b2         MVGIPMFADQPDNMVHVEAKGAAVTVN-LNFMKKDDLRDAVNTVINDKSYKENALRLSGIHHDRPMSPLDEAVFWMEFTMRNKGAKHLRVQAHELTWYQY   489
m2b1         MVGIPMFADQPDNMNHMKAKGAAVILN-LNFMTSEDFRDAINTVVNDKSYKENAMRLSGIHHDRPMSARDEAVFWIEFTMRNKGAKHLRVHSHKLTWYQY   484
z2a3         MVGLPLFADQPDNLLHMKSKGAAVVLD-INTLESKDLVDALKTVLNNPSYKESIMRLSRVHHDQPMKPLDQAVYWIEFVMRNKGAKHLRVQAHELSWYQY   485
z2a4         MVGLPLFADQPDNLLHMKSKGAAVVLD-INTLESKDLVDALKTVLNNPSYKESIMRLSRIHHDQPMKPLDQAVYWIEFVMRNKGAKHLRVQAHELSWYQY   486
z2a5         MVGLPLFGDQPDNLLHIKTKGAAVVLD-IHTMGSKDLVDALKAVLNNPSYKESIMRLSRIHHDQPMKPLDQAVYWIEFVMRNKGAKHLRVQAHELSWYQY   485
z2a6         MVGLPLFGDQPDNLMHLKSKGAAVVLD-FFTLESTDLVDALKAVVNNPSYKESIMRLSRIHHDQPMKPLDQAVYWIEYVMRNKGAKHLRVQAHELSWYQY   485
z2a2         MVGLPLFADQPDNLLHVKSKGAAVVLD-INTLESKDLVDALKTVLNNPSYKESIMRLSRIHHDQPMKPLDQAVYWIEFVMRNKGAKHLRVQAHELSWYQY   487
z2a1         MVGLPLFADQPDNLLHMKSKGAAVVLD-INTLESKDLVDALKTVLNNPSYKESIMRLSRIHHDQPMKPLDQAVYWIEFVMRNKGAKHLRVQAHELSWYQY   492
f2a1         VVGIPLFGDQPDNLARLSRLGVAVVLD-FNQMTSDQLTEALNGVIAKKSYKSNMLRLSLIHHDQPATPLETAVFWVEFVMRQGGAKHLRVASHDLNWFQY   488
m2a1         VVGVPLFGDQPDNLARISRHGAALVLN-FNSMTADDLTEALQAVINQPSFRTSMQRLSAIQRNQPVAPLSTAVFWVEFVMRHGGAKHLRLASHDLTWIQY   493


             :.:*.   .          .          :
h2a1         HSLDVIGFLLVCVTTAIFLVIQCCLFSCQKFGKIGKKK--------KRE   527
h2a2         HSLDVIGFLLVCVTTAIFLVIQCCLFSCQKFGKIGKKK--------KRE   528
h2b28        HSLDVIGFLLACVATVIFVVTKFCLFCFWKFARKGKKG--------KRD   529
h2b11        HSLDVIGFLLACVATVIFIITKFCLFCFWKFARKGKKG--------KRD   529
h2b10        HSLDVIGFLLACVATVLFIITKCCLFCFWKFARKGKKG--------KRD   528
h2b7         HSLDVIGFLLVCVATVIFIVTKCCLFCFWKFARKAKKG--------KND   529
h2b4         HSLDVTGFLLACVATVIFIITK-CLFCVWKFVRTGKKG--------KRD   528
h2b15        HSLDVIAFLLACVATVIFIITKFCLFCFRKLAKKGKKK--------KRD   530
h2b17        HSLDVIAFLLACVATMIFMITKCCLFCFRKLAKTGKKK--------KRD   530
h2a3         YSIDVIGFLLACVATAIFLFTKCFLFSCQKFNKTRKIE--------KRE   527
l2a1         HCLDVIAFLISCVALFMFIIVKCCTFCCRKCGITRKTK--------LE-   528
l2a2         HCLDVIAFLISCVALFMFIIVKCCTFCCRKCGITRKTK--------LE-   527
l2a5         HCLDVIAFLISCVALFMFIMVKCCSFCCRKCGITRKTK--------IE-   528
l2a4         HCLDVIAFLISCVALFMFIIVKCCSFCCRKCGITRKTK--------IE-   523
zf2a1        HSLDVLAFLFTCTATIVFILFKCCLCCCRRCGRIAKRK--------KE-   533
c2a1         HCLDVLAFLFICAAIAGFILVKCCMFCCRKCGRVTKKK--------KE-   533
zf2a2        HSLDVLAFLFTCTATIVFILFKCCLCCCRRCGRIAKRK--------KE-   526
c2a2         HCLDVLAFLFICAAIAGFILVKCCMFCCRKCGRVTKKK--------KE-   526
zf2a3        HSLDVLAFLFTCTATIVFILFKCCLCCCRRCGRIAKRK--------KE-   529
c2a3         HCLDVLAFLFICAAIAGFILVKCCMFCCRKCGRVTKKK--------KE-   529
x2a1         HCLDVIGFMFVCLLLFFYITITFLTFCYKKCCRTTKEK--------NQ-   526
x2a2         HCLDVIGFMFVCLLLFFYITITFLTFCYKKCCRTTKEK--------NQ-   526
z2b1         HCLDVFAFLTTVLTLVLYICSKMAKFFIMRCCFRSKRK-------SKKE   527
z2b3         HCLDVFAFLTTVLTLVLYICFKMAKFFIMRCCFRSKRK-------SKKE   527
z2b5         HCLDVFAFLTTVLTLVLYICFKMAKFFIMRCCFRSKRK-------SKKE   527
z2b6         HCLDVFAFLTTVLTLVPYICFKMAKFFIMRCCFRSKRK-------SKKE   527
f2b1         HSLDVLALLLSVVLLSMFVFIKTCRFCCRRCCSRR---------KNKTE   529
f2b2         HSLDVLAFLLTIILLLAALFTKTCCFCLRRCCGRKGPTKKTKKMKKKAE   538
f2b3         HSLDVLAFLLTVALLLVFFFIKICCFCIRRCCSRKAQG------KRKFE   532
s2b4         HSLDVLAFVLAIVLVLVLLFVKTCSFCFRRCCGRKAKAKK-----RKAE   533
s2b5         HSLDVLALVLAIDLVLVLLFVKTCSFCFCRCCGRKAKAKK-----RKAE   533
s2b3         HSLDVLAFVLAIVLVLVLLFVKTCSFCFRRCCGRKAKAKK-----RKAE   530
s2b1         HSLDVLAFVLAIVLVFVLLFVKTCSFCFRRCCGRKATAKK-----RKAE   533
s2b2         HSLDVLAFVLAIVLVLVLLFVKTCSFCFRRCCGRKAKAK------RKAE   532
m2b1         HSLDVLVAFLCLDLFLIFLFIGTCRFCFRRCCG--SKAVK-----TKAE   526
z2a3         HCLDVAAFLLSIAALITFLWVKTCCFLFRRCVRKTHPERKTQ--KNKKE   532
z2a4         HCLDVAAFLLSIAALITFLWVKTCCFLFRRCVRKTHPERKTQ--KNKKE   533
z2a5         HCLDVAAFLLSITALITFLWVKACCFLFRRCVRKTRPERKTQ--KNKKE   532
z2a6         HCLDVAAFLLSITALITFLWVKACCFLFRRCVRKTRPERKSK--KE---   529
z2a2         HCLDVAAFLLSIAALITFLWVKTCCFLFRRCVRKTHPERKTQ--KNKKE   534
z2a1         HCLDVAAFLLSIAALITFLWVKTCCFLFRRCVRKTHPERKTQ--KNKKE   539
f2a1         HSFDVVGVLLVTLMTFVALCWS-VVRCLLRCCRRQRRL--------KGD   528
m2a1         YSLDTGATLLLGLLTLGAVSWAGLLHILQRCRRRERRE--------KKD   534
```
