# Supplementary material for: Cloning and Comparative Analyses of the Zebrafish Ugt Repertoire Reveal Its Evolutionary Diversity
Source: PLoS One. 2010 Feb 10;5(2):e9144. doi: 10.1371/journal.pone.0009144 (PMC2819257; doi:10.1371/journal.pone.0009144)
Supplement: Figure S3 — An alignment of the vertebrate Ugt5 protein sequences. The zebrafish (z), fugu (f), tetraodon (t), medaka (m), stickleback (s), and xenopus (x) Ugt2 polypeptides were aligned by using the ClustalX. The amino acid residues are represented by capitalized single-letters with the degree of conservation highlighted by differential coloring. The names of the Ugt5 proteins are indicated on the left and presented according to the order of the eight Ugt5 groups (Ugt5a to Ugt5h) shown on the Figure 5. (0.28 MB PDF) [file pone.0009144.s005.pdf]

```
                                        .:*: . :.*:* ::  ::  *  :** . :.*   . ::   :  :  . :
z5a1        ------MHHSARLCLFSLLCVLSGTYGGKVLVFPLDGSHWVNMKVLIEELHRKGHNVTVVRPSNSWYIKEESPFYSPITIPNTGGF---DEEFSGLLINR    91
z5a2        ------MHSHIHLGLFLLLCVLSRSYAGKLLVVPVEGSHWVNMKVLIVELHSRGHSITVVRGSSSWYIEEQSPLYTSITVDN-GVF---DDDFMEKFFPH    90
z5a3        ------MHPSTHLGLFLLLCVLSRSYAGKLLVVPVEGSHWVNMKVLIVELHSRGHSITVVRGSSSWYIEEQSPLYTSITVDN-GVF---DDDFMETFLPH    90
z5a4        -----MTHPSTHLKLFLLLCVLSRSHAGKLLVVPVDGSHWINMKVLLVELHSKGHSITVVRASNSWYIEEQSPLYTSITVEN-GEF---DADFEEKFLPH    91
z5a5        -------MPSLPATLLAFLSILSLVFGGKVLVFPLDGSHWVNMKVLIEELHTRGHNVTVLRASNSWYIKKESPFYTSITVYNSGGI---EKELFEEFSRN    90
f5a1        ------MLRPTSLPLLLLICTVSVVNAGKVLVYPFDGSHWINMKVLIEELHTRGHEVTVVRPADSWYIKAESSYYKSITLNYSAGI---NEETLGLYVTK    91
f5a2        ------ML----LQAVVLLYLTSVVNAGKVLVYPLDGSHWINMKVLIEELHARGHEVTVVRPSDSWYIKAESSYYKSITLSSSAGF---DEEGFGLFATE    87
f5a4        ------MLRPTSLPLLLLICTVSVVNAGKVLVYPLDGSHWVNMKVLIEELHSRGHEVTVVRPADSWYIKAESSYYKSITLSSSAGF---DEEGFGLFVEK    91
f5a5        ---MTWPLSPLLTVALLFIQVPVPSSAGKILVFPMDGSHWVNMKVLIEELHTKGHEVTVVRASDSWYISQKSPFYRSVTLLGSNGF---EDN-METYLVQ    93
t5a1        ------MLPPTSLSLLLLLLCSSLVNGGKVLVYPFDGSHWINMKVLIEELHARGHELTVLRPADSWYIKAESPHYKSITLKSSAGV---DKETLGVYVTR    91
t5a2        ------MLPPTSLSLLLLLCCFSVANGGKVLVYPLDGSHWINMKVIIEELYSRGHEVTVLRPADSWYIKAESPHYKSITLKSSAGF---DEEGFELFATE    91
m5a1        ------MYRQTLVALAVFFYSSFLVNGGKVLVFPVDGSHWVNMKVIIEELHARGHEVTVLRPSDSWYIKPDSPHYKAITLNSPAGF---DSKGFGSFVTR    91
s5a1        ------MYRPALAALAVLLCSSLPVNGGKILVVPVDGSHWINMKVLVEELHARGHEITVMRSSDSWYIKPESPHYRSITVNSSAGF---DEEGFGLFVDT    91
s5a2        ---MRALRSSLIIFALLLVQLSS-SSWGKILVFPLDGSHWVNMKVIIEELHARGHEITVVRPSDSWYITEKSPFYTSVTISSPGGF---NQKYFEAFLAR    93
z5b1        -MIGQTFQPCGQILIALLLVTVPTAQSEKVLVFPVDGSHWVNMNILVEALHAKGHNITVIRMADSWYIKEFSPHYTSINLKSAGGF---DEEFFETFAFR    96
z5b2        -MNGQWFQVIWPITLTLLLPSVPVAQCGKVLVFPVDGSHWVNMNILVEALSAKGHDVTVMRTASSCYIKEFSPHYTSINLKSEGGY---NKEVLEQFASR    96
z5b3        -MNGKVFHMRWPVTLTLLLVTVPVVESGNVLVFPVDGSHWVNMNVLVEALHAKGHNITVIRMADSWYIKEFSPHYTSVNLKSSGGL---SENFIGPFVSR    96
z5b4        -MNRKVLHMLWPVTLCLLLVMAPVVESGNVLVFPVDGSHWVNMNILVEALHAKGHNITVIRMSDSWYIKEFSPHYTSITLKSSGGF---SEDILETFASR    96
f5b2        ------MQVLW-LWMALCFLCPTVVHGGKVLVFPIDGSHWVNMKVIIEALHSRGHQVSVVRSSDSWYIKEKSPLYNSITLDSPSGF---DESFITTFVAR    90
t5b3        ------MQRLW-LWIDICVLCLTAVHGGKVLVYTMDGSQWVNMEGIIEALNARGHQISVVRSSDSWYIKEKSQFYTSITLDSPSGF---DEDFITMFVAR    90
t5b4        ------MQRLW-LWIDLCVLCSTAVHGGKVLVFPMDGSHWVNMEGIIEALHARGHQISVVRSSDSWYIKEKSQFYTSITLDSPSGF---DEDFITMLVTR    90
t5b5        ------MQRLW-LWIGLCVLCSTVVHGGKLLVFPSDGSHWLNMEVMIEALHSRGHQIVVVRTSDSWYIKEKSQFYTSITLDRPTGF---DRDFITTLVAR    90
z5c1        MAQLSKNTFSVSLWITVMIIFGSLCDGGKILVAPLEGSHWVNMDILIKALHGQGHTVDVIRNTNSWFVKENSTYYNSITIPDSKGM---DEEFVEDVLTK    97
z5c2        MTRVPNNMVSVSLWTTVITIFGSLCDGGKILVAPLEGSHWVNMDILIKALHGQGHTVDVIRNANSWFIKENSTYYDTITIPNTKGW---DEEFAADFVYK    97
z5c3        MAPLFKNLCSVSLWITIIPLFGFLCDGGKILVVPLEGSHWLNMDILIKALHGKGHSIDVIRNTNSWYVKEKSDYYNSITIPQSKGM---DEEFVDDMISK    97
f5c1        --MSLQGKAAFVFVLSISSLLWGVSHGGRILVVPLEGSHWVNIDILIEALHSRGHTVDVIRSSQNWYIQDGAQHYNTITVPVTEGF---NHDFIIPIVKR    95
f5c2        ----MRPIFFCSTSVLLFVARPRSCQGGNILVVPIEGSHWINMKILLQALHARGHNITIVRSSKSWYISENSTYYKTVTVPVERSL---DQRSITRIVSE    93
m5c1        -MISRRSSSGVFLFFSVIFCSWRIGQAGRILVVPMEGSHWVNMDIMIKALHFRGHHVDVVRTNKSWYIKDDAPHYKTVTVSVAKTF---DKDFLNPILKK    96
m5c2        -MISRRSSSGVFLFFSVIFCSWRIGQAGQILVVPMEGSHWVNMDIMIKALHFRGHHVDVVRTNKSWYIKDDALHYKTVTVSVVKTF---DKDFLNPILKK    96
m5c4        -MISRRSSSGVFLVFSVIFWSWRIGQAGRILIFPLEGSHWVNMDIMIKALHSRGHHIDVVRTNKSWYIKDDAPHYKTVTVSVAKTF---DKDFINPILKK    96
m5c5        ----MK-LPLCCCALLVLVLTTSFCHGGNILVFPAEGSHWINMDILLQALHSRGHSLTVLRSSKSWYIKEHAPHYKSFTVPVERSM---DQKLITEFITE    92
m5c7        ----MK-LPLCCCVLLVLLLTPSFCHGGNILVFPAEGSHWINMDILLQALHSRGHSLTVLRSTKSWYIKEHAPHYKSFTVPVERSI---DEKFISKTVAD    92
s5c1        -MPPNRYAT-SLLLLSIVLLSWRICHGRKILIFPLEGSHWVNMDIVVKALHSRGHSVDVVRTDKSWYIKEDSPHYKTITVPVTEAF---NPDFINPILKK    95
s5c2        ----MR-LIYCSSVLLLLVLFPRECQGGNILVLPIEGSHWINMDVLLQALHSRGHNITIVRSSKSWYVKDKSPYYNILTVQVERSM---DQKFIGEAINH    92
z5d1        MKSFFSQSNVITGLFLALSAFTLSCSGGKILLYPVDGSHWVNMKVLIEELHSRGHSITVIRPKSSWYITEKSPLYTSITIQDNVND---FENFFEDYLSK    97
m5d1        ---MHQVCGVFVFFNLCLHFLAPSCSGGNILVYPIDGSHWINMKILLEELHARGHNITVIRVSTSWYIKEKSPIYSSITVEV-EEA---LEDFFDVFIKE    93
m5d2        ---MYRVCGGLACFSLCLIFLVPSCSGGNILVYPVDGSHWINMKILLEELHARGHNITVIRVSTSWYIKEKSPIYSSITLEM-EEA---LEDFFDVFVKE    93
z5e1        ----MLLHPQISVLVAFIWSLPSLSLAGKILVYPVDGSHWLNMNILLQELHQRGHKLTVVRSANSWYIPENATHYTSITIQVNHLSSLEDPKYMASFLKR    96
f5e1        ----MASYPVLTFLVFFSAALSSTCDGGKVLVYPLDGSHWLNMKILLELLHSRGHEITVIRSSTSWYISEVSPYYTSITIAQDHSHHIESQDFMTSFLKR    96
t5e1        ----MASCPALVVLLFFSAALSSTCQGGKVLVYPLDGSHWLNMKVLVELLHSQGHEVTVVRSSTSWYISEVSPDYTSITITQKHSHHIESQDSMTSFLKR    96
t5e2        ----MASCPALVVLLFFSAALSSTCQGGKVLVYPLDGSHWLNMKILVELLHSQGHEITVVRSSTSWYISEGSPDYTSITITQKHSQHIESQDFMTSFLKR    96
m5e1        ----MASFPALMFFLLFTTFLNSGCESGKVLVYPMDGSHWTNMIKILEVLHSKGHHITVFRSSNSWYISKSSPYYTSITIPQEKPQNVESQTFMTTFLRN    96
m5e2        ----MASFPALMFFLLFTTFLNSGCESGKVLVYPVDGSHWTNMIKILEVLHSKGHQITIFRSSNSWYISESSPYYTSITIPQEKPQNVESQTYMTTFLRK    96
s5e1        ----MESHPALAFLLLFSAALSSGCDGGKVLVYPVDGSHWLNMKILVEALHSQGHQITVVRSSTSWYVSEFSPHYTSITVTQEHTQNLESQDYMTLFLKR    96
z5f1        ------MDIIFVVQIVVLSLTLP-VHCGKILVFPHEGSHWLNLNTLIEELHHRGHQISVIRALDSWYIPETSPHYDSINVPFLLGG---DDQFYHSFIFR    90
f5f1        ------MFLLFATVICLPTSMIPEVLGGNVLVFPHEGSHWVNMRVLIEELHSRGHNVTVVRAADSWYISQTSPHYKSITLDVAVGA---NNTFFHHFVTE    91
m5f1        ------MTM-LWVWTCFFWCCVSVVHSGKVLVFPVDGSHWVNMRVIIEELHSRGHQVTVIRHTDSWYIKETSPFYTSITVNLKND----SKNIVASFVSK    89
m5f2        ------MTM-LWVWSCVFWCCVSLVHSGKVLVFPVDGSHWVNMRVIIEELHSRGHQVTVIRHTNSWYIKETSPFYTSIRVNLKND----SKNIVAAFVSK    89
m5f4        ------MAMSLTSIFCLVLVIVPVSQGGKILVFPHDGSHWVNMKVLVEELNLRGHSLTVIRTSDSWYITDTSPFYNTLTIDVKGGG---NEEFFRVFVSE    91
s5f1        ------MFLTLVSIIFLLAGIIPAAYGGKVLVFPHDGSHWVNMRVLVDELHSRGHAVTVIRAADSWYISETSPHYSAVTVDVAGGA---DEAFFRLFVSQ    91
z5g1        -MDMSSK---WLSALTCLLSVVCCCHGGNILVFPEDGSHWVNMQVILRKLHTHGHSFTVVRSSKSWYIHENSALYNTITVNPVET-EDVGQDYFNMLLDR    95
z5g2        -MQGCMS--LVFLWTSLLLSLPSGYNADKILVFPIDGSHWVNMEVLVKKLQSRGHELSVIRLEDSWFIKGNSPYYSSITVTLKK--KFIDLDLFETAVKK    95
f5g1        -MSRIIP--VFLAGLCILLLGPTLGSSSRILVVPVDGSHWINMEILLRELHTRGHSITVLRSPKSWYIPSESEIFSSINVTMLE--DKTDMSYYNKLLLD    95
t5g1        -MSRIFP--VVLSGLCVLLLGPTLGSGGRILVAPVDGSHWINMEILLRKLHARGHNITVLRSTKSWYIPSDSSFYTSINVTMLE--DQTDKSYYNKLLLD    95
m5g1        -MSPAVSGLLTVLGFLLLLLGGSPCSSSKILVVPVDGSHWVNMKVILEELHSRGHELTVLRSAKSWYISSNSSFYTSINVPMLE--DEADREFYHKMIQD    97
s5g1        -MSGVIP--LFLAGLCFLFLRPTCCTGSRILVVPADGSHWINMEEILRELHSRGHDITVLRSTKSWYIPNNSSIYTSINVTMQE--DELDPDFFNKLLQD    95
s5g2        -MSGVIP--SFLAGLCFLLLRPTCCSGSRILVVPVDGSHWINMEVILRELHSRGHNITVLRSAKSWYIPNNSSIYTSINVTMLE--DESDRNFYNRMLQN    95
f5h1        -MRGLSAVSLTVLLVGVSLIASPSVHGGKILVFPVDGSHWVNMNLIVQSLHARGHEVTVVRTSTSWYIKEIAPHYRSITISLPEAICIEEPNFFHSFLSE    99
t5h1        -MGGISAGSLAILLVGVSLMTYSSVHGGRILVFPVDGSHWLHMNLIVQSLHARGHEVSVVRSATSWYVQESASHYRSLTVSLPEAICIEEPDFFHSFLSE    99
s5h1        --MGFNQG-VLLLLASTSLLTGSGVLAGKILVFPVDGSHWVNMNLLIQSLHSRGHEVTVVRTATSWYVKENAPHYRSLTVTLPGAISIEEEDFFVTFLVK    97
x5a1        ---MAPMRRMLGTSVFLCAAVLSSVQAGKILVVPVEASHWINIKILMMELVQKGHELTVLRPSNSLYIDEYSEDFRVINVSMAENQVMSRDEFEKYALEF    97
```

```
               .                                           . .   .   .    ::  * **    * .:.  : :*     ::    : .
z5a1          LLEIKRGG------YFVLNRVRLEFEIMMKFKTMHEDMLQMMDRMLEDE-KVMNSIQDAKFDVVLADPAVGGGPILAYKFNIPLVFNVRWTIQGEGHFAI   184
z5a2          LLKIQRQG------KSFWNEMALADEMIHFVTETHKQSCNMTAILFKNE-TLMNSLKDAAYDVVLTDPAYGSGVLLAHRLGLPLVYNVRWTVYGEAHFDI   183
z5a3          LLKIQRQG------KSFWNEMALADEMIHFVTETHKQSCNMTAILFENE-TLMNSLKDAAYDVVLTDPAYGSGVLLAHRLGLPLVYNVRWTVYGEAHFDI   183
z5a4          LLKMQRQG------KSFWTEMALADETYSRFTEIHQEICKMTATMFENV-TLMNTLKDAAYDVVLTDPAFGSGVLLAHRLGLPLVYNVRWTMYGEAHFVI   184
z5a5          ILVIRRQK------RSFWTKLKLVSEVGAVFEEMHRNQLNLMGNIFENK-DLMDSLQTAKYDLLLTDPAVGGGFFLAYRLGLPLVLNVRWTMHGEAHFEI   183
f5a1          TLKMRREG------ASLWSRLKLEYDLVELFRQIHKHTVEMVGEMFENT-QLMQILRDAKYDLVLTDPAGGGGVILAHRLGLPLVFNVRWTVQGEGHQAI   184
f5a2          LLAMRREG------ASFWNLLTLEYKMMETFSIMNNLVIQIAGEMFENT-QLMQSLHDAKYDLVLTDPAVPTGVLLAHRLGLPLVLNVRWTVQGEGHQAI   180
f5a4          MLAMRREG------ASLWSRLFVEY---EAVYQMNKHVLEMVREMFEDT-QLMQTLHNAKYDLVLTDPATGPGVLLAHRLGLPLVFNVRWTVQGEGHRAI   181
f5a5          QLEIRLAGRNASSWSRFWTNLQLRQVVVHQFSQFHKGMSEMVTQMFEDE-KLMLSLQEAKYDVLLTDPGFGGGAILARRLQVPLIYNVRWTIQGEAHLLI   192
t5a1          TLKMRREG------ASLWSHLRLEYDLMEFFYQIHKRVVEMIVEMFEDA-ELMQSLRDANYDLVLTDPAGGGGVLLAHRLGLPLVLNVRWTVQGEGHEAI   184
t5a2          LLAMRREG------ASLWNQLSLEFKMMETFAEMIKLVVQIAEEMFEDA-ELMQSLRDAKYDLVLTDPAVATGVLLAHRLGLPLVLNVRWTVQGEGHEAI   184
m5a1          TLNMQRQG------ASIWTRISLELDMLNNFNEMNKLVLQMVEEIFEDP-KLMQSLHEAKYDLVLTDPAIGGGVLLAHRLGLPLVLNVRWTIQGEGHQAI   184
s5a1          MMNMRRKG------ASLWARISLEFELVEKFYQMNKEVIEMVGKMFENT-KLMQSLTDAKYDLFLTDPAIGAGVLLGHRLGLPLVFNVRWTVQGEGHRAI   184
s5a2          QLEIRLQGRHGSFWSKIWTKIQIERLVVEQFSQFHKGMSEIAVQMLEDE-NLMQSFLEAKYDVVLTDPGVGVGAMLARRLQVPLVFNVRWTIQGEAHFLM   192
z5b1          LMKIVREG-------SRWGRLKLEIETWQSTYEMIEIESEMIKNMLEDQ-PLMQTFRDAKYDLLLTDPLLFGGVILGHFLKLPIVYNVRWTMYNEAHFVS   188
z5b2          IMHILRDG-------STWGHLKLQADMWQSFLKIFEAEREMIVNMIEDQ-QLMLSLKDAKYDLILTDPNMFGGVILGHYLKLPMVYNVRWAGYNEAHFSI   188
z5b3          LMNILREG-------STWGRVKLEIEMWQTSLKMIEIEREMITNMIEDQ-QLMQSLKKAKFDLILTDPAMYGGVMLGHYLKLPIVYNVRWTIFGEGHFAI   188
z5b4          LITILRDG-------STWGRLKLEMEMWQSSLKIMETEREMIINMIEDQ-QLIDSLKKTKYDLFLTDPAMYGGVILGNYLKLPIVYNVRWAIFGEGHFVI   188
f5b2          LLEIQREGK------SFWTRYKLEIEQAEKTREMFEKTSEFVKMLLENK-DLMQSIKNTNYDLVLTDPVAPVGAILANYLKLPLVFNARWTSHGEGHFDI   183
t5b3          LLEIQREGK------SFWTRCKLEFEQTVKGFEMLEKASESIKWLLDNK-EIMQSIKDTNFDLILSDPVAPFGAIMANYLKLPLVFNVRWTSHGEGHFEI   183
t5b4          LLEIQRKGK------SFWTRCKLEFEQAVKGFEMLEKASESIKWLLDNK-EIMQSIKNTNFDLILSDPVAPFGAIMANYLKLPLVFNVRWTSHGEGHFEI   183
t5b5          LLEIKREGK------SLWTRCKLEFEQVGKAYKVFTKASGAMKWLLDNK-EIMQSIKNTNFDLILSDPVAPFGAIMANYLKLPLVFNVRWMTHGDGHFEI   183
z5c1          KIDYERGKG------SWLGGVQLFLDVSKAVYESHKLVCRLITNIFESE-DILKALQEKKYDLMLTDPGWGTGIILAHKLKLPMVYNVRWTTPGEGHFDI   190
z5c2          MIKYEKEKG------SWLSIVRLYLNVFDAVKKTHEMVCQLITNILDSE-EILKMLNEKQYDLMLTDPVWGTGIILAHKLKLPMVYNVRWTTTGEGHFDI   190
z5c3          VINHERGKS------SWLSTVKLFFDSVIDNYEMTKMVCDITTSIFESE-EILKTLQEKQYDLMLTDPVWGMGIILAHKLKLPMVYNVRWTTTVDGHFNI   190
f5c1          IIDIERGER------PAWNFVSLQLEMFTSMYRMHRLICQMAANIFTDR-NLMNTLKENKYDLALVDPAWGAGLMLAHALKLPLAYYVRWITSGEGHFAI   188
f5c2          IIQFERGAL------PLTSFLHITFGMTSTFIDAHSAIGEFIAATLDDQ-ELMRTLKENKYDLVLTDPSWGSGIILAKYLNLPLVYNVRWLLSGEGHLAF   186
m5c1          IFAIERGKT------SIMSFISLLIEMFGTMFDMHGMMCKMATTMLEDK-ELMGVLRRKKYDLVLTDPVWGAGIILAHALELPLVYNVRWVTSGEGHLTI   189
m5c2          VFAYRREKT------SIMNFVFLLIEMFRTMFDMHGMMCEMATTVLEDK-ELMGDLRKKKYDLVLTDPVWGAGIILAHALELPLVYNVRWVTSGEGHLTI   189
m5c4          IFAIERGKT------SIMSFVSLQIEMFMAMFDMHGMMCKMATTMLEDK-ELMGVLRRKKYDLVLTDPAWGAGIILAHALELPLVYNVRWVTSGEGHLTI   189
m5c5          FIHFQRGAL------PITSFLHMSLGMIGKLREAHEPVGEFVTAAMNDK-ALMKTLNESKFDVMLTDPCWGGGVILAKYLNLPLVFNVRWLVPEEAHFAI   185
m5c7          FLQFERGAL------PLTSFLFSSVGVIGVFFEAHSAVCELVSAVLDDA-ALMKTLNESKFDLVLTDPCWGGGAILAKYLNLPLVYNVRWLTPMEAHFSI   185
s5c1          VIDIERGES------SVWSFASLQAEMFSAMFNVHRIMCKMATDMFEDK-DLMKTLKDRKYDLVLTDPAWGAGIILAHALKLPLVYNVRWITSGEGHMAI   188
s5c2          ALEFERGAL------PLANFLHLTVGMFGTFVDAHAIVGELLSAMLDDK-EFMRTVKEGNFDMVLTDPCWGHGIILAKYLNLPLVYNIRWLIAGEAHLAI   185
z5d1          AMEIERGEG------SGLAFLKLQYDLFSMLSTAHEIACKMVSIILEDK-MLVKKLQDEQYDLMLTDPAIAGGVFLAHYLKLPLVLNVRWITSGEGHFAI   190
m5d1          QIRVQREGA------SLSSFFRLHKDFISMMSHAHALRCNATKHIFENK-NLVKQIIDSKFDLVLTDPAMSAGVVLAKYFNLPVVLNVRWITSGEGHFVL   186
m5d2          QIKVQREGA------SMLSFFKLNKDFLSMLTQAHVLWCDVTKDIFENE-DLAKQITDSKYDVVLTDPAIGGGLVLAKYFNLPVVLNVRWIPVGEGHFVL   186
z5e1          NIDIQRGKG------SIWSFLALQKEIITLLEESHRSTAEMVRVVLEDE-KLIKTLKESKYDLMLTDPGFAGGVILGAYLGLPMVFNVRWITNAEGHFAI   189
f5e1          SIEIRRREG------SLWAFFEFYQNLFNMLEESQQDVAKMVIDIFENE-TLIMKLKKTEYDFMLLDPAFPGGVLLAHYLQLPTVLSARWAFSGEAHSII   189
t5e1          SIDIRRREG------SLRGFFEYYQNLYTFLEQSQQDVAKMVIDIFENK-TLSTKLKETNYNFVLTDPAFPAGVLLAHYLQLPLVLSARWSFSSEAEFII   189
t5e2          SIDIRRREG------SLRGFFEFYQNLFHMVRENQQDVAKMVTDIFENK-TLIRNLKDKEYNFVLTDPAFPAGVLLAHYLQLPVVLNARWAYNGDGHFII   189
m5e1          TLEGRQYQG------TLWGLFEFYRNFSKLLKENQQLVANLAASIMENK-TLVKELNQTKYDVFLTDPVFPAGVLLAHHLKLPLVLNVRWLSDGDAHYPI   189
m5e2          TLEDRQYQG------TLWGLFEFYRNFFNLIQGNQQVVANLAASIIENK-TLVKELNQTKYDVFLTDPVFPAGVLLAHHLKLPLVLNVRWLSDGDAHYPI   189
s5e1          SIEIRRNKG------TLWAFVKFYNNLFDMMGENHKVVAKLIVSIFENK-TLIAELKEAGYDLFLTDPAFPGGVLLAHYLQLPLVFNVRWLFNGEAHFAI   189
z5f1          QLQIRREQR--------WTRFKLDMELKEKFSEMHRKICEMVIHIVEKEPAIMKQIKEANFDIMLTDPANGGGVVLARYLHLPLVFNVQWTVHGEAHFAI   182
f5f1          VVKIKRHQG------NAWSRFGLDSELKNNFYELHRKICEAIVHMFENE-DLMESFQDSKFDIVLTDPANGGGVLLAHRLGLPLVFNARWTVHGEAHFAV   184
m5f1          NIEIKRRRN------PAWLRFLQEIEHFPQFLEFHKITCTTLERIFENQ-ELMQLLKNTNYDLILTDPATPGGAILAHYLKLPLVFNVRWTTHGEGHFII   182
m5f2          NIEIKRGRN------PAWLRFLQEIEYFPQFLEFHKITCTTLERIFENQ-ELMQMLKNTNYDLILTDPATPGGAILAHYLKLPLVFNVRFTTYGEGHFII   182
m5f4          VIKIKRSKA------SAWAQFSLNMVLKDGFSHFHEKVGEMIVCMFENA-TLMRSLQEAKYDVVLTDPGNGGGVFLAHYLSLPIVLNARWTIHGEAHFAI   184
s5f1          VIKIKRSKS------SAWARFALDMELKDKFFELHKKICEVVTNIFENK-ELMKSFRDSKFDAVLTDPANGGGVMLAHYLGLPLMFNARWTVHGEAHFAI   184
z5g1          SLALQKMSP-------LVRFFEQQKDITTVLKVFHNGVLRMVSAMLDDA-ELVKSLREAKFDLLLTDPAFPAGVLVANFLQLPMVYNVRWLNAGDAHMQT   187
z5g2          ILDARRDGP-------IMGVLAQMSEFIGILKVGHGANIAMLTSMLENK-VLMSQIKMANYDLMLTDPAMPGGVILAHYLHLPMVYNVRWMSFGEGHFSI   187
f5g1          VMECRQSGE-------FLRSFCQQHLITSMLDKGHKILASAAATMLDDP-KFMKKLTDAKFDLMLTDPGLTIGVVLGSYLKLPMVFNVRWINTGESHFTI   187
t5g1          VIECRRSGQ-------FLRSFCQQHLITSMFNKGHKILANSASAMLDNL-KFMKKLRDAKFDLMLTDPALTIGLILGSYLKLPMVFNVRWINTGESHFTM   187
m5g1          VLDCRRHPS-------FIRTFCQQEVMTFAFSNGHRILARAATTIIEDQ-ALMKKIRDTEFDLMLTDPALPLGVVLGGYLKLPMVFNVRWINLGEGHLTI   189
s5g1          AMECRDFQP-------SIGTFCQEHLITSLLGNGHEILARSAAAMLDDP-VFMKKLHDEKYDLMLTDPAMTLGVILGSYLKLPMVFNVRWINNGEGHLTI   187
s5g2          VMECRRSPH-------FISTFCQQNLVTSMLGKGHEILARSAAVMLDDP-VFMKKLHDAKYDLMLTDPALTLGVILGSYLKLPMVFNVRWINNGEGHLTI   187
f5h1          MLKIQKDGG------ALS-FVRFYWEVLTTLSVIHQQASMMGVEIFKNK-TLLQSLHDTEFDVVLIDPGLPVGVLVAHELKLPTVFNVRWVTAGDGHFVV   191
t5h1          MLTIQKAGG------ALS-FVRFYWKVLAALSRIHRQASLMGVEILQNK-TLLQRLRNTQFDVVLVDPGLPVGALVAHELKLPTVFNVRWVTSGDGHFVV   191
s5h1          MLAIHKEGA------SISGFVKFYWEMLGTLSNIHRQASLLAVEMFENK-TLMQSIRNTQFDVVLLDPGLPVGVLVAHELKLPTVFNVRWITSGEGHFVV   190
x5a1          VFTIALSKH--------SSPFSLAWNFIQTVQTGTQIVSVAVSALFENN-NIMNELRNTRFDLVLADPYNIAGPMLAHHLKLPMVFFGRWMPTEDIHFVT   188
```

```
          :*** *.:*       * * :  :  * .                         :        :   *:::* : :* :::*** ***  * .**:   ..
z5a1      APSPLSYIPVPGVELTDKMSFLQRVKNVLIYLISQIQMQIMVEPIYAPFCHKHFGPNVTYFSLFQDADIWLMRNDFTFEFPRPTMPNIVYMSGFQCKPAK   284
z5a2      APSPLSYVPVSGLQLTDKMTFSQRVMNMMTYIMIRYKYSKNFGSPYQELTQKYFGPNVNFFSLLQDADLWLMRNDFTFEFPRPTMPNVVYMGGFQCKPAK   283
z5a3      APSPLSYVPVSGLQLTDKMTFSQRVMNMMTYIMIRYKYSKNFGSPYQELAQKYFGPNVNFFSLLQDADLWLMRNDFTFEFPRPTMPNVVYMGGFQRKPAK   283
z5a4      APSPLSYVPVSGLQLTDKMTFSQRVMNMMTYIMILYKNFKYFGSPYQELTQKYFGPNVNFFSLLQDADLWLMRNDFTFEFPRPTMPNVVYMGGFQCKPAK   284
z5a5      APSPLSYIPVPGLELTDKMTFVQRVQNILVYMFSKFQKARTVSKHYRSFCEKYFGSEINYKAILQDADIWLMRNDFTFEFPRPTMPNIVYMGGFQCKPSK   283
f5a1      APSPLSYVPMAMSELTDQMTFFQRVKNILVFVLSQLRIKYLADPNYRPFVHRYFGDDVHYMELFQAADIWLMRNDFTFEFPRPTMPNVIYMSGFQCKPSK   284
f5a2      APSPLSYVPIAMSELTDQMTFFQRVKNILYYLFNQFKIWNFIDPILITFVHRYFGDDVHYMELFQSADIWLMRNDFTFEFPRPTMPNVIYMSGFQCKPSK   280
f5a4      APSPLSYVPIPGTELTDQMTFWQRVQNIMYYFFSCWQIWYFVDPNYRPFVHRYFGDDVHYMELFQSADIWLMRNDFTFEFPRPTMPNVIYMSGFQCKPSK   281
f5a5      APSPLSYVPFTAAELTDKMTFPQRIKNLLSYILGMYTMSSITEPCYKPLVEKYFGPDVDYSTFFLDADIWLMRNDFVFEFPRPTMPNIVYMGGFQCKAPK   292
t5a1      APSPLSYVPKAMSELTDKMTFFQRVKNVLFFVLTQLRIKYVADPNYKPFVHRYFGSDVHYMELFQAADIWLMRNDFVFEFPRPTMPNVIYMSGFQCKPSK   284
t5a2      APSPLSYVPKAMSELTDKMTFFQRVKNTLFYLFNQFKIRNFIDPVLNPSVHRYFGSDVHYMELFQAADIWLMRNDFIFEFPRPTMPNVIYMSGFQCKPSK   284
m5a1      TPSPLSYVPIPGSELTDKMSFKQRIKNILYYIFTCLQIWYITEPNYKPFVHRYFGSDIHYMELFQAADIWLMRNDFTLEFPRPTMPNIVYMSGFQCKPSK   284
s5a1      APSPLSYVPIPGAELSDKMTFPQRVKNLLYFFFTCFQTWYVTDSNYKPFVRRYFGDDVHYMELFQAADIWLMRNDFTFEFPRPTMPNVVYMSGFQCKPSK   284
s5a2      APSPLSYIPFTSTELTDKMTFPQRIKNVLSYNLGMYTMSCITEPYYKPVVKKHFGLDVDYSTFFLDADIWLMRNDFVFEFPRPTMPNIIYISGFQCKPPK   292
z5b1      APSPLSYVPFMMVES-DRMSFFQRVKNVVMFTVAEAQAALLFAPYYNALCEQFIGPGVSFLSLVQGADLWLHRVDFIFEYPRPTMPNIIYMAGFQCKPSK   287
z5b2      APSPLSYVPMPSLELSDRMSFLQRVYNVVMYIFIEIGAALVISPINDALAERFIGPGTSFLSLVQGADLWLHRVDFVFEYPRPTMPNVIYMAGFQCKPSK   288
z5b3      APSPLSYVPVPMLEIPDRMSFLERVKNVVMYIITETQIAFLIAPTYNALCERFIGPEVNFDNLVQEADLWLHRADFVFEYPRPTMPNIVYMAGFQCKPSK   288
z5b4      APSPLSYVPVAMLELPDRMSFLERVKNVVMYIITETQIAFLIAPTYNVLCERFIGPGVNLFNLVQGADLWLHRADFVFEYPRPTMPNIVYMAGFQCKPSK   288
f5b2      APSPISYIPLTGTQLSDQMSFFERLQNVLMFGFLQYQIHWYVAPTFEGLIKKYFGPGTDYISLFQAADLWLMRVDFVFEYPRPTMPNVVYIGGFQCKPAK   283
t5b3      APSPLSYIPLMGTQFSDRMSFFQRLQNVLTFGFMKAQLAWVTIPSYDDLIKKYLGPDIEYTSLFQAADLWLMRVDFVFEFPRPTMPNVVYMGGFQCKPAK   283
t5b4      APSPLSYIPLMGTQFSDRMSFFQRLQNVLTFGFMKAQLAWVTIPSYDDLIKTYLGPDIEYTSLFQAADLWLMRVDFVFEFPRPTMPNVVYMGGFQCKPAK   283
t5b5      APSPLSYVPLMGTQFSDRMSFFQRLQNVLAYGFMKALLSWVVIPSYDDLIKTYLGPDIEYTSLFQAADLWLMRVDFVFEFPRPTMPNVVYMGGFQCKPAK   283
z5c1      APSPMSYIPLTGSGNTDKMSFFQRVINVFYYLLLDFQCSRFNVPQYQALCDKYFDPPVDFYKLLQGADLWLMRVDFVFEFPRPTMPNIIYTGGFQCTPTK   290
z5c2      APSPMSYIPITGSGNTDRMSFFQRLKNYFFYLLLDLQLSHFNVKQYQAICDKYFTSRVNFHELLQGADLWLMRVDFVFEFPRPTMPNIIYIGGFQCPPAK   290
z5c3      APSPMSYIPITGSGNTDRMSFFQRVKNIIFYFTNDIKYSRFSLPQYQAICDKYFDPPVDFYQLLQGADIWLMRVDFVFEFPRPTMPNIIYTGGFQCTPAK   290
f5c1      APSPLSHIPVIGSGLSDIMTFSQRVKNLFFYMIWEAQNLFLIQPQYQAVCDHFFGPEVRYSELIQGADLWLMRVDFVFEYPRPTMPNVVYMGGFQCKPAK   288
f5c2      APSPISYIPITGSGHSDKMTFIQRVKNVILYLITKYHFDLLS-QVYQKICDKYLGPGHDFKQIMLDADIWLMRVDFVFEYPRPTMPNVVYMGGFQCKPAR   285
m5c1      TPSPLSYIPMTCSRLTDKMTFIQRVKNLLFYALWEIQDMLFICPQYQTVCDQFFGPNVKYNHLLQGADLWLMRVDFVFEFPRPTMPNVVYIGGFHCKPSK   289
m5c2      TPSPLSYIPMTCSRLTDKMTFIQRVKNLLFYALWEIQDMLFICPQYQTVCNQFFGPNVKYNHLLQGADLWLMRVDFVFEFPRPTMPNVVYIGGFHCKPSK   289
m5c4      TPSPLSYIPMTGSGLTDKMTFIQRVKNLLFYALWEIQDMLFICPQYQTVCDQFFGPNVKYTHLLQGADLWLMRVDFVFEFPRPTMPNVVYVGGFHCKPSK   289
m5c5      APSPLSYVPIVGSGNTDRMNFFQRVKNMFLYLVTQLQSYTAVNYVYQPICDKFLGPDHDFNRLVLDADIWLMRTDFVFDYPRPTMPNVVYMGGFQCKPAK   285
m5c7      APSPLSYVPVTGSGNTDRMTFLQRVKNVILYLVTYTQNDVLAKQIYQPTCDKYLGPDHDFNQIKTDADIWLMRSDFVFDYPRPTMPNVVYMGGFQCKPAK   285
s5c1      APSPLSYIPLTGSGLSDKMTFIQRVKNHLFSIIWLAQDVLLIRPQYQALCDQFFSSEVRYIDLLHGADLWLMRVDFVFEFPRPTMPNVVYMGGFQCKPAE   288
s5c2      APSPVSYIPITGSGNTDKMIFFQRVKNMILHIITQTQQQLVIKLIYQKLCDKYLGPDIDYNHLVLNADIWLMRVDFVFEFPRPTMPNVVYMGGFQCKPAE   285
z5d1      APSPMSYIPLPGSGHTDKMGFAQRVKNVLFKTFTFLQNRFVVGPHYDILIDKYLDYKTDIVGLIQAADIWLMRADFVFEFPRPTMPNIVYMGGFRCKPSK   290
m5d1      APSPLSYIPVPGSGLTDRMDFIQRLKNIFFYGIINFQQIVMVGPIYDDICSKYIQGGCSIVSLLQQADIWLFRSDFVFDFPRPTMPNVVYIGGFQCKPAE   286
m5d2      APSPLSYIPVPGSGLTDRMDFIQRLKNIFFNCITKFQELFLVGPIYDDICSKYIEGGCSIVSLLQQADIWLFRSDFVFDFPRPTMPNVVYIGGFQCKPAE   286
z5e1      APSPLSYIPTIGSRVTDKMSFANKLKNIMHFGIGQYIDHMLTRPLYQGVISKYIDPNTNVYALIQGADLWLMRVDFVFEFPRPTMPNVVYIGGFQCKPSK   289
f5e1      APSPLSYVPQVLSYNSDKMDFFQRMKNVISHMMLIYMHYFVVNPPYQAVCDKYFGADVNVMSLMQGADLWLIRTDFIFEFPRPTMPNVVYIGGFQGKPSK   289
t5e1      APSPLSYVPQLFSYNSDQMDFLQRIKNVISYLMLVYMHYYVTDPPYKSLCDKYFGPEVNILSLMQGADLWLIRSDFVFEFPRPTMPNVVYIGGFQGRPSK   289
t5e2      APSPLSYVPQLFSYNSDQMDFHQRMKNVISHLMLVYMMHFVSDPPYQAVCDKYFGPEVNVMSLMQGADLWLMRSDFVFEFPRPTMPNVVYIGGFQGRPSK   289
m5e1      TPSPLSYIPALFSHNTDKMSFFQRVKNVFCKGMLVYLYYYISNPPYQALCDRYFEHNVNVMSLMQGADLWLMRTDFTFDFPRPTMPNIIYIGGFQCKPAN   289
m5e2      TPSPLSYIPALFSHNTDKMSFFQRVKNVFCKGMLVYLYYYISNPPYQALCDRYFEHNVNVMSLMQGADLWLMRTDFTFDFPRPTMPNIIYIGGFQCKPAN   289
s5e1      APSPLSYVPHLFTDNSDKMDFFQRINNMIRRTILIYIYYFVSNPPYQAVCDQYFGPDVSVMSLIQGADLWLMRVDFIFEFPRPSMPNVVYIGGFQGKPSK   289
z5f1      APSPPSYVPFPLSQLADSMSFPQRIYNVLFSAVRLFLYRRTVGPHYSALCNRLFGPGLDYFELFQAADIWLMRADFVFDFPRPTMPNVIYIGGFHCSPAK   282
f5f1      APSPLSYVPLPPSELSDKMNFIERVKNLLFYIMRMHLYKQVVGPHYSAVVSRYFDQDTDYFSLFQAADLWLMRVDFAFEFPRPTMPNVVYMGGFQCKPAK   284
m5f1      APSPLSYIPVG-IEFSDKMSFFHRVINVLFFYFGQHSFEKMITPHYQDFMKKYFGPDVDFYSMFQAADLWLMRVDFVFEFPRPTMPNVVYMGGFQCKPAK   281
m5f2      APSPLSYIPVG-IEFSDKMSFFQRVINVLFFYFGQHSFEKMITPHYQDFMKKYFGPDVDFYSMFQAADLWLMRVDFVFEFPRPTMPNVVYMGGFQCKPAK   281
m5f4      APSPLSYVPLPPSELTDLMTFPERVKNVIYYITRMHLYKQTVRPHYSPLTLRYFGPDVDYFSLFQAADLWLMRVDFVFEFPRPTMPNVVYMGGFQCKPAK   284
s5f1      APSPLSYVPLPPSELTDQMTFFERVKNVVFYTMRMHLYKQIVAPHYSALSRRYFGPDADYFSLFQAADLWLMRVDFVFEFPRPTMPNVIYMGGFQCKPAK   284
z5g1      APSPPSYVPMYNSLLHDQMSFLQRVENFLRYLCSLLQERYVIVPIYRELLERHFPPGSDLLSMQQSADIWLMRMDFVFDFPRPTMPNIIYMGGFQCKPAQ   287
z5g2      APSPISFVPVPGSGLTDRMGLLERSRNFLHYGLNLIQERWMVIPMYTSLLLNHFPPGTDLLTMQRSAELWLVRADFVFEFPRPSMPNLVYIGGFQCRPAK   287
f5g1      APSPVSYVPVPGSELHDQMDFPGRIKNMLLYLHSLVEQYFIINPAYSELLQKHFPPGTDLLSLQLAADIWLVRVDFVFEFPRPTMPNVVYIGGFQCQEAQ   287
t5g1      APSPVSYVPVPNSELHDRMDFLGRFKNMLLYLHSVVEQHLIINPAYSELFQKHFPPGTDLLSLQLAADIWLVRVDFVFEFPRPTMPNMVYIGGFQCQEAQ   287
m5g1      APSPLSYVPISGTELHDKMDFLDRVKNMLHYLHTVVEQHFIVNPAYSDLFRRYFPPGTDLLSLQYSAEIWLLRADFVFEFPRPTMPNVVYIGGFQCKEAQ   289
s5g1      APSPVSYVPVSGSELRDRMDFLERTKNMLHYLYSFCEQQFIINPPYADLLQRHFPPGTDLLSLELAADIWLVRTDFVFEFPRPTMPNLVYIGGFQCKPSN   287
s5g2      APSPVSYVPVSGSELRDRMDFLERTKNMLHYLYSFFEQQFIINPPYADLLQRHFPPGTDLLSLELAADIWLVRTNFVFEFPRPTMPNLVYIGGFQCKPSN   287
f5h1      APSPTSYVPISGNAMSDRMTFAQRLKNTLYYFLNTCIDRYVVCPHYDRLVEKFFGSDVNFYHLLQSADLWLIRVDFVFEFPRPTMPNVAYIGGFQCKPSE   291
t5h1      APSPTSYVPVSGNAVSDRMTFAQRLKNTLYYFFNACFDKYIVCPHYDRIVEKFFGPDVTFYHLLQGADLWLIRVEFVFEFPRPTMPNVIYIGGFQCKPPK   291
s5h1      APSPTSYVPTSGYAASDKMNFAQRVKNTFHYFLNTCIDRFIVCPHYDRLVDRYFGPDVKFYHLLQGADIWLMRVDFVFEFPRPTMPNIAYIGGFQSKPSK   290
x5a1      APSPLSYVPVINSRQTDKMYFSERVTNVLLFSMYYVTSHLLIYPVYDKLCQLYLHTDVGLLEMYKKADIYLMKVDFVFEFPRPIMPNAVYIGGFQCKPTK   288
```

```
        .*   :: *  .:.   .:.:::*:..  :       ..  :: :..:::* . .     :..** : .*:**.*:*.*   : *: *.* **: *::::
z5a1    PLPNDLEKFVESSGEHGVIVMSLGTLIAQLPQDMTDDIAAAFAELPQKVIWRYTGPRPVTLGNNTLLVEWLPQNDLLGHPQIKVFVAHGGTNGIQEAIYH   384
z5a2    PLPGDLEEFVQSSGEHGVIMMSLGTVFGQLLSELNDEIAAAFAQLPQKVIWKYTGPRPANLGNNTLIVNWLPQNDLLGHPKTKLFVAHGGTNGLQEAIYH   383
z5a3    PLPGDLEEFVQSSGEHGVIMMSLGTVFGQLLSELNDEIAAAFAQLPQKVIWRYTGPRPANLGNNTLIVNWLPQNDLLGHPKTKLFVAHGGTNGLQEAIYH   383
z5a4    PLPGELEEFVQSSGEHGVIMMSLGAVFGQLLSEPNDEIAAAFAQLPQNVIWRYTGPRPANLGNNTLIVNWLPQNDLLGHPKTKLFVAHGGTNGLQEAIYH   384
z5a5    PLPDDLEEFVKSSGDHGLIVMSLGTLFTHLPEDITEEIAAAFAGLPQKIIWRHTGPRPVNIGDNTLLVDWLPQNDLLGHAKTKVFITHGGTNGIQEAIYH   383
f5a1    PLSKELEDFVQSSGEHGVIIMTLGTLVGNLPEDIVEDIAAAFAQLPQKVIWRHKGKRPSTLGNNTLLLDWLPQNDLLGHPKTKLFVAHGGTNGIQEAIYH   384
f5a2    PLSKELEDFVQSSGEHGVIIMTLGTLVGNLPEDIVEDIAAAFAQLPQKVIWRHKGKRPSTLGNNTLLLDWLPQNDLLGHPKTKLFVAHGGTNGIQEAIYH   380
f5a4    PLSKELEDFVQSSGEHGVIIMTLGTLVENLPKDVVEDIAAAFAQLPQKVIWRHKGKRPSTLGNNTLLLDWLPQNDLLGHPKTKLFVAHGGTNGIQEAIYH   381
f5a5    PLPADLEEFVQSSGEHGVVVMTLGTLVADLPRDVAEEIAAGFARLPHKVVWRYVGRRPSSLGNNTLLVDWLPQNDLLAHPKARVFVTHGGTNGVQEAIFH   392
t5a1    PLSQELEDFVQSSGEHGVIIMTLGTLVEKLPEDIVEDIAAAFAQLPQKVIWRHKGKKPSTLGNNTLLMDWLPQNDLLGHPKTRLFVAHGGTNGVQEAIYH   384
t5a2    PLSQELEDFVQSSGEHGVIIMTLGTLVGKLPEDIVEDIAAAFAQLPQKVIWRHKGKKPSTLGNNTLLVDWLPQNDLLGHPKTRLFVAHGGTNGVQEAIYH   384
m5a1    PLPKELEDFVESSGEHGVIVMTLGTLVERLPDDITEDIAAAFAELSQKVIWKHKGRRPNNLGNNTLVLDWLPQNDLLGHPKTRVFVAHGGTNGIQEAIYH   384
s5a1    PLSKELEDFVQSSGEHGVIVMTLGTLVAKLPEDITEEIAAAFAQLPQKVIWRHKGKRPSTIGNNTLLLDWLPQNDLLGHPKTRVFVAHGGTNGIQEAIYH   384
s5a2    PLPADLEEFVQGSGDHGVVMMTLGTLVGELPQDIAEEIAAAFAQLPQKVVWRYVGQRPANLGNNTLLVNWLPQKDLLGHPKTRVFVTHGGTNGVQEAIYH   392
z5b1    PLPQDLEDFVQSSGDHGVIIMSLGTLVGQLPDDVAEAIAEAFAELPQKIIWRYKGKRPSALGNNTLVMDWMPQNDLLGHPKTRAFVAHGGTNGVQEAIYH   387
z5b2    PLPQDLEDFVQSSGDHGVIIMSLGTLIDKLPDDVAEAIAEAFAELPQEIIWRYKGKRPSALGNNTLIMDWMPQNDLLGHPKTRAFVAHGGTNGIQEAIYH   388
z5b3    PLPQDLEDFVQSSGNHGVIVMSLGTLIGQLPDDVAEAIAEAFAELPQKIIWRYKGKRPSALGNNTLVMDWMPQNDLLGHPKTRAFVAHGGTNGVQEAIYH   388
z5b4    PLPKDLEDFVQSSGDHGVIIMSLGTIIGQLLDDVAEAIAEAFAELPQKIIWRYKGKRPSALGNNTLIMDWMPQNDLLGHPKTRAFVAHGGTNGVQEAIYH   388
f5b2    PLPEHLEEFVQSSGEHGVIIMSLGTFIAELPQDLADQIAAAFAKLPQKVIWRYKGAKPATLGNNTLLVDWMPQNDLLGHPKTKLFVAHGGTNGVQEALYH   383
t5b3    PLPEHLEEFAQSSGEHGLIIMSLGTFIAELPPDLADEIAAAFAKLPQKVIWRYKGAKPATLGNNTLLVDWMPQNDLLGHPKTKLFVAHGGTNGVQEAIYH   383
t5b4    PLPEHLEEFAQSSGEHGLIIMSLGTFIAELPPDLADEIAAAFAKLPQKVIWRYKGAKPATLGNNTLLVDWMPQNDLLGHPKTKLFVAHGGTNGVQEAIYH   383
t5b5    PLPEHLEEFAQSSGEHGLIIMSLGTFIAELPPDLADEIAAAFAKLPQKVIWRYQGAKPATLGNNTLLVDWMPQNDLLGHPKTKLFVSHGGTNGIQEAIYH   383
z5c1    PLPHDLEDFMQSSGDHGVIVMSLGSFISVLPDYVSSEIAAAFARLPQKVIWRYTGKKPSTLGNNTLLVDWMPQKDLLGHPKTKLFIAHGGTNGVQEALYH   390
z5c2    PLPHDLEDFMQSSGDHGVIVMSLGSLIGNLPENVTAEIVAAFARLPQKVIWRYTGKKPSTLSNNTLMVDWMPQKDLLGHPKTKVFISHGGTNGVLEALYH   390
z5c3    PLPHDLEDFMQSSGDHGVIVMSLGTFISALPEDVTAEIAAAFARLPQKVIWRYTGKKPSTLGNNTLLVDWMPQKDLLGHPKTKVFVAHGGTNGVQEALYH   390
f5c1    PLPEHLEEFVQSSGEHGVIIMSLGTFVSQLPAEITNEIAAAFAKLPQKIIWKHEGDRPATLGNNTLLVDWMPQNDLLGHPKTKLFVAHGGTNGVQEAMYH   388
f5c2    PLPEHLEEFVQSSGEHRVIIMSLGTFVSQLPAEITNEVAAAFAKLPQKIIWKHEGDRPATLGNNTLLVDWMPQNDLLGHPKTKLFVAHGGTNGVQEAMYH   385
m5c1    PLPEHLEEFVQSSGEHGVIIMSLGTFVSELPDDMANEIAAAFAKLPQKVIWSYRGSRPSTLGNNTLVVDWIPQNDLLGHPKIKLFVAHGGTNGVQEAIYH   389
m5c2    PLPEHLEKFVQSSGEHGVIIMSLGTFINELPEDMANEIAAAFAKLPQKVIWSYRGSRPSTLGNNTLVVDWIPQNDLLGHPKIKLFVAHGGTNGVQEAIYH   389
m5c4    PLPEHLEKFVQSSGEHGVIIMSLGTFVSELPDDMANEIAAAFAKLPQKIIWSYKGSRPSSLGNNTLVVDWMPQNDLLGHPKIKLFVAHGGTNGVQEAIYH   389
m5c5    PLPEHLEEFVQSSGEHGVIIMSLGTFVSELPDDLANEIAAAFAKLPQKVIWRYKGKRPASLGNNTLVVDWMPQNDLLGHPKIKLFVAHGGTNGVQETLYH   385
m5c7    PLPEHLEEFVQSSGEHGFIILSLGTFINELPDDLANEIAAAFAKLPQKVIWRYKGKRPASLGNNTLVVDWMPQNDLLGHPKIKLFVAHGGTNGVQETLYH   385
s5c1    PLPEHLEEFVQSSGEHGVIIMSLGTFVSQLPADISNEMAAAFAKLPQKVIWRHKGVRPATLGNNTLLVDWMPQKDLLGHPKIKLFVAHGGTNGVQEAIYH   388
s5c2    PLPEHLEEFVQSSGEHGVIIMSLGTFVSELPADISNEMAAAFAKLPQKVIWRHKGVRPATLGNNTLLVDWMPQKDLLGHPKIKLFVAHGGTNGVQEAIYH   385
z5d1    PLPADLEAFAQSSGEHGFIIMSLGTLVKSLPADMANAIAAAFARLPQKVIWRHLGDRPSNVGNNTLIVDWMPQNDLLGHSKIKAFVAHGGTNGVQEAIFH   390
m5d1    PLPADLEKFVQSSGEHGVIIMTLGTLVNELPQDVANEIAEVFSKMPQKVIWKHKGDRPSTLGDNTLIVDWMPQKDLLGHSQVKVFVAHGGTNGVQEAIYH   386
m5d2    PLPADLEKFVQSSGEHGVIIMTLGTLVNALPQDVANEIAEVFSKMPQKVIWKHKGDRPSTLGNNTLIVDWMPQKDLLGHPQVKVFVAHGGTNGVQEAIYH   386
z5e1    PLPDELNKFVESSGEHGVVIMSLGTLLGSLVPDISEIVASAFAQLPQKVIWRHVGEKPSTLGNNTLVVDWLPQNDLLGHPKTKAFVTHGGTNGIYEAIYH   389
f5e1    PLPADLEDFMQSSGEHGVVVMSLGTLLGDLGPELSEIIASAFANLPQKVVWRHIGERPTSLGNNTMLVKWLPQNDILGHPKTKLFMSHGGTNGIYEAIYH   389
t5e1    PLPADLEEFMQSSGEHGVVVMSLGTLLGDLGPELSEIVASAFASLPQKVVWRHIGERPTSLGNNTLLVKWLPQNDVLGHPKTKLFISHGGTNGIYEAIYH   389
t5e2    PLPADLEEFMQSSGEHGVVVMSLGTLLGDLGPELSEIVASAFASLPQKVVWRHIGERPTSLGNNTLLVKWLPQNDVLGHPKTKLFISHGGTNGIYEAIYH   389
m5e1    PLPADLEDFMQSSGEHGVILMTLGTLLSDLGPKVSEIFAAAFANLPQKVLWRHIGEIPATLGSNTMLVKWLPQNDILGHPKTRLFVTHGGTNGLYEAIFH   389
m5e2    PLPADLEDFMQSSGEHGVILMTLGTLLSDLGPKVSEIFAAAFANLPQKVLWRHIGEIPATLGSNTMLVKWLPQNDILGHPKTRLFVTHGGTNGLYEAIFQ   389
s5e1    PLSSDLEAFVQSSGDHGVVVMTLGTLLSDLGPEISEILASAFANLPQKVVWRHIGKRPINLGNNTMLVEWLPQNDLLGHHKTKVFVTHAGTNGIYEAIYH   389
z5f1    ALPKDLEDFLQSSGEHGVIVMSLGTLVAQLPMDIADEIVAAFAELPQKVIWRYTGDRPANVGNNTLLVNWLPQNDLLGHPKTRVFVSHGGTNGVFEAIYH   382
f5f1    PLPEHLEEFVQSSGEHGVIIMSLGTFIAELPQDLADKVAAAFAKLPQKVIWRYKGAKPATLGSNTLLVDWMPQNDLLGHPAIKLFISHGGTNGIYEAIYH   384
m5f1    PLPDHLEEFVQSSGEHGFIIMSLGTLIADLPADLAEEIAAAFAELPQKVIWRYKGSRPSTLGNNTLLVDWLPQNDLLGHPHIQLFVSHGGTNGIYEAIYH   381
m5f2    PLPDLLEEFVQSSGEHGFIIMSLGTLIADLPADLAEEIAAAFAELPQKVIWRYKGSRPSTLGNNTLLVDWLPQNDLLGHPHIQLFVSHGGTNGIYEAIYH   381
m5f4    PLPDHLEEFVQSSGEHGFIIMSLGTLIADLPADLAEEIAAAFAELPQKVIWRYKGSRPSTLGNNTLLVDWLPQNDLLGHPHIQLFVSHGGTNGIYEAIYH   384
s5f1    PLPEHLEEFVQSSGEHGVIIMSLGTLIGEFPSDLADEIAAAFAILPQKVIWRYKGDRPAALGNNTLLVDWMPQNDLLGHKSIKLFVSHGGTNGIYEAIYH   384
z5g1    ALPVDLEEFMQSSGEHGVVFMSLGAMVGALPRTITEAIASAFAKIPQKVMWRYHGERPSTLGNNTLLLEWFPQNDLLGHPKTRAFVSHGGTNGIYEAIYH   387
z5g2    PLPADLEEFMQSSGDHGVVVMSLGTLIAGLPKEVMEAIASAFAQIPQKVIWRFIGQRPSTLGNNTLLIQWLPQNDLLGHPKTRAFVAHGGTNGLYEAIYH   387
f5g1    PLPAEMEAFMQSSGEHGVVVMSLGTLVSALPLEVTEAIAAAFAELPQKVVWKFLGEKPSFLGNNTMLTKWLPQNDLLGHPKTRAFVAHGGTNGMYEAIYH   387
t5g1    PLPAELEAFMRSSGEHGVVVMSLGTIVSALPREVTEAMASAFAELPQKVVWKFVGEKPSSLGNNTMLTKWMPQNDILGHPKTRAFVAHGGTNGMYEAIYH   387
m5g1    PLPDEFETFVQSSGEHGVVVMSLGTLVSALPRETTEAIAAVFAQLPQKVIWRFVGEKPSSLGNNTRIVNWLPQNDLLGHPKTRAFVAHGGTNGVYEAIYH   389
s5g1    PLPVELEAFMQSSGEHGVVVMSLGTFVSAINDQATEAIAAAFAELPQKVVWRFGGGKPLCLGNNTLLMDWLPQRDLLGHPKTRAFVAHGGTNGMYEAIYH   387
s5g2    PLPHELEAFMQSSGEHGVVIMSLGTFVSSIDRQATDAIAAAFAEIPQKVVWRFVGEKPLSLGNNTLLVDWLPQKDLLGHPKTRAFVAHGGTNGMYEAIYH   387
f5h1    PLLSELEDFVQSSGEYGFVLMSLGTLVQGLPLEITSEIAAAFAQIPQKVIWRHTGKSPKNLGNNTFLVKWLPQNDLLGHPKIKAFVGHGGTNGIYESIYH   391
t5h1    PLSPELEDFVLSSGEHGFVLMSLGTLVQCLPLEITSEITAAFAQIPQKVIWRHVGEQPKNLGNNTLLVKWLPQNDLLGHPKIKAFVGHGGTNGIYESIYH   391
s5h1    PLSSELEEFVQSSGEHGFILMSLGTLVNGLPIEITSEIAAAFSQIPQKVIWRHASAPPNNLGNNTLLVKWMPQNDLLAHPKIKAFVAHGGTNGIYESMYH   390
x5a1    PLPHHLQQFMDGAS-QGVVVFSMGTLVKYLPYNIAREIAAGLARLPEKVIWRYSGEKLDTLGNNTLIADWIPQNDILGHPNTKAFLAHGGENGVYEAIYH   387
```

```
                :*::.. .  **  *. ::. :. .  :           :   :   :     *   :  :* :  *    *:: * :* *..:*   * ** :    :
z5a1    GVPILGLPLVFDQPDNLFRMEAKGTAKIVD-IATLD-KTVFLEALKEVLYNP-SYRENMQRLSKLHHDQPLKPLDRAMFWIEFVMRNKGAPHLRTQSFRM   481
z5a2    GVPIVGLPLAFDQPDNLSRMRAKGTAKIVG-FATLD-RAVFLEALKEVLHNP-SYRENMQRLSKLHHDQPMKPLDRAIFWIEFVMRNKEAPHLRAQSFRM   480
z5a3    GVPIVGLPLAFDQPDNLSRMRAKGTAKIVE-FATLD-RAVFLEALKEVLHNP-SYRENMQKLSKLHHDQPMKPLDRAIFWIEFVMRNKGAPHLRTQSFRM   480
z5a4    GVPIVGLPLAFDQLDNLSRMRAKGTAKIVE-FATLD-RAVFLEALKEVLHNP-SYRENMQRLSKLHHDQPMKPLDRAIFWIEFVMRNKGAPHLRAQSFRM   481
z5a5    GVPILGLPLVFDQPDNLSRMKVRGTAKLLD-IAELN-RTMFLEALKEVLYNP-SYKENIQRLSKVHHDQPMKPLDRAMFWIEFVMRNKGASHLRTQSFRM   480
f5a1    GVPLVGLPLMFDQQDNFFRMKVKGVAKVLD-IATVN-KDNFLEALKEVLHQP-SYREKMKTLSSLHRDQPMKPMDQAMFWIEFVMRHKGAKHLRTESYKM   481
f5a2    GVPLVGLPLMFDQQENFFRMKVKGVAKVLD-IATVN-KDNFLEALKEVLHQP-SYREKMKTLSNLHRDQPMKPMDRAMFWIEFVMRHKGAKHLRTESYKM   477
f5a4    GVPLVGLPLMFDQQDNFFRMKVKGVAKVLD-IATVN-KDNFLEALKEVLHQP-SYREKMKTLSNLHRDQPMKPMDRAMFWIEFVMRHKGAKHLRTESYKM   478
f5a5    GIPVVGLPLFLDQPDNLSRLRAKGGAVVLD-IAVLD-RDVFAEALETVLHDP-SYRENMQRLSRLHRDQPVKPLDLAVFWIEYVMRHKGAQHLRTHANKM   489
t5a1    GVPLVGLPLMFDQQDNFFRMKVKGVAKVLD-IATVN-KDNFLEALKEVLYQP-SYRENMKTLSNLQRDQPIKPMDQAMFWIEFVMRHKGAKHLRTESYKM   481
t5a2    GVPLVGLPLMFDQQDNFFRMKVKGVAKVLD-IATVN-KDNFLEALKEVLYQP-SYRENMKTLSNLQRDQPIKPMDQAMFWIEFVMRHKGAKHLRTESYKM   481
m5a1    GVPLVGLPLMFDQHDNFFRMQARGVAKVLD-FGTLN-KDTFLEGLKEVLYTP-SYRENMKKLSSLHRDQPMKPLDRAVFWIEFVMRHKGAAHLKTDSYKM   481
s5a1    GVPLVGLPLMFDQPDNFFRMKTRGVAKVLD-IATVN-KENFLEALKDVLYDP-TYMEKMKVLSGLHRDQPMKPLDRAMFWIEFVMRHKGAAHLRTDSYKM   481
s5a2    GVPVVGLPLFFDQPDNLSRIKAKGGAVILD-IAELD-RHVFADALQAALYNS-SYRENMQRLSRLSRDKPMKPLDQAVFWIEYVIRHKGARHLTTQSTKM   489
z5b1    GVPIIGFGLIFDQPDNLEKMRVRGAANNVD-FATMD-KESFLKTVKEVLYDP-SYQENMQRLSRLHKDVPVKPLDNAIFWIEFVMRHKGAAHLRTESYKM   484
z5b2    GVPIIGFGLIFDQPDNLSKMRVRGAAKNVD-FATMN-KESFLTTVKEVLYDP-SYRENMQRLSRLHKDVPVKPLDNAIFWIEFVMRHKGAAHLRTESYKM   485
z5b3    GVPIIGFGLIFDQPDNLSKMRVRGAAKNVD-FATTD-KESFLTTVKEVLYDP-SYRENMQRLSRLHKDVPVKPLDNAIFWIEFVMRHKGAAHLRTESYKM   485
z5b4    GVPIIGFGLIFDQPDNLSKMRVRGAAKNVD-FATMD-KESFLKTVKEVLYDP-SYRENMQRLSRLHKDVPVKPLDNAIFWIEFVMRHKGAAHLRTESYKM   485
f5b2    GVPIIGLPLIFDQPDNVHRLEVRGAGKVLD-FFTMT-EEIFFQGIQEVLNDP-SYRMNMQRLSRLHRDAPMKPMDSALFWIEFVMRHKGAAHLRTESYRL   480
t5b3    GVPVIGLPLIFDQPDNVRRLEVRGAGKVLD-FFTLT-EEIFSQGIQEVLHDP-SYRTNMQRLSRLHRDQPMKPMDTALFWIEFVMRHKGAAHLRTESYKL   480
t5b4    GVPVIGLPLIFDQPDNVRRLEVRGAGKVLD-FFTLT-EEIFSQGIQEVLHDP-SYRTNMQRLSRLHRDQPMKPMDTALFWIEFVMRHKGAAHLRTESYKL   480
t5b5    GVPVIGLPLIFDQPDNVRRLEVRGAGKVLD-FFTLT-EEIFSQGIQEVLHDP-SYRTNMQRLSRLHRDQPMKPMDTAVFWIEFVMRHKGAAHLRTESYKL   480
z5c1    GVPVIGIPFFFDQYDNLIRLQARGGAKIVS-LAELG-ENSLHAAIQEVINEP-SYRLNMQKLSYLHRDKPVEPLDSAIFWIEFVMRHKGAAHLRTESYKM   487
z5c2    GVPVIGIPFFFDQYDNLIRLQARGGAKLLS-IADLG-ENTLHAAIQEVINEP-SYRLNMHKLSHLHKDKPVRPLDSAIFWIEFVMRHKGAAHLRTESYKM   487
z5c3    GVPVVGIPFFFDQYDNLIRLQARGGAKIVS-LAELG-ENSLHAVIKEVINNP-SYRLTMQKLSQLHLDKPVKPLDSAIFWIEFVMRHKGAAHLRTESYKM   487
f5c1    GVPVVGLPVFFDQYDNLLRLKERGGAEILS-LRTVDKDDNFLAAVKRVLNDP-SYRMNMQRLSGLHRDKPMKPMDSALFWIEFVMRHKGAAHLRTESYRL   486
f5c2    GVPVVGLPVFFDQYDNLLRLKERGGAEILS-LRTVDKDDNFLAAVKRVLNDP-SYRMNMQRLSRLHRDAPIKPMDSALFWIEFVMRHKGAAHLRTESYRL   483
m5c1    GVPVVGLPIFHDQYENLLRLQARGAAKIIT-PATVDKDDNFLKAIQEVLTEP-SYRMNMQRLSRLHRDQPVKPMDKALFWIEFVMRHKGAAHLVTESNKM   487
m5c2    GVPVVGLPIFHDQYENLLRLQARGAAKIIT-PATVDKDDNFLKAIQEVLTEP-SYRMNMQRLSRLHRDQPVKPMDKALFWIEFVMRHKGAAHLVTESNKM   487
m5c4    GVPVVGLPIFYDQYDNLLRLQARGAAKIIT-PATVDKDDNFLKAIQEVLTEP-SYRMNMQRLSRLHRDQPVKPMDKALFWIEFVMRHKGAAHLVTESNKM   487
m5c5    GVPVVGLPLFFDQYDNLLRLQARDGAIVLS-ITTVDKDDSFLKAIQEVLTEP-SYRMNMQRLSRLHRDQPVKPMDKALFWIEFVMRHKGAAHLKAQSSNM   483
m5c7    GVPVVGLPLFFDQYDNLLRLQERGGAKILS-VSTVEKDDSFLKAIQEVLTEP-SYRMNMQRLSRLHRDQQVKPMDKALFWIEFVMRHKGAAHLKAQSYNM   483
s5c1    GVPVVGLPLFFDQYDNLLRLQERGAAKILT-LATVDKDNIFLNAIQEVLHEP-SYRRNMQRLSRLHRDQPMKPLDNALFWVEFVMRHKGAAHLRTESYKM   486
s5c2    GVPVVGLPLFFDQYDNLLRLQVRGAAKLLT-IKTVDKDNNFLNAIQEVLNEP-SYRRNMQTLSRLHRDQPMKPLDNALFWVEFVMRHKGAAHLRTESYKM   483
z5d1    GVPVLGVPLFFDQFDNLIRVQGKGAGKILK-LSELN-AEAFEQALRESLNDG-SYKRNMQTLSTLHRDQPMRPLDTAIFWIEHVIRHKGATHLRSEFYKM   487
m5d1    GVPVLGIPLFFDQYDNLLRLQERGAGKILQ-LADLN-GRTFEEGLKEVLHNI-SYRQNIQRLSCLHKDKPMSPMDQAIFWIEYVIRHKGARHLISEAYKM   483
m5d2    GVPVLGIPLFFDQYDNLLRLQERGAGKILQ-LADLN-GRNFEEGLKEVLHNS-SYRQNIQRLSRLHKDKPMSPMDQAIFWIEYVIRHKGARHLISEAYKM   483
z5e1    GVPMLGLPLIFDQFDNIIRLEARGVARMLD-VATLD-VDILTQALKDILDEKQSYQKNMRKMSSLHRDTPLKPLDSAIFWLEFVMRHKGAAHLRTESYKL   487
f5e1    GVPILGLPLIFDQIDNFVRMKARGAAEMVD-VTTLD-VESLTNTLKNILDPTKAYKEKIQKLSQLHHDKPMKPIDSALFWMEFVMRHKGASHLRTESYKL   487
t5e1    GVPVLGLPLIFDQMDNFVRMKARGAAEIVD-VTTVD-VESLTKTLRNILHPANSYGERMKKLSQLHHDKPMKPLDSALFWMEFVMRHKGAPHLRTESYKL   487
t5e2    GVPVLGLPLIFDQMDNFVRMKARGAAEIVD-VTTVD-VESLTKTLRNILHPANSYGERMKKLSQLHHDKPMKPLDSALFWMEFVMRHKGAPHLRTESYKL   487
m5e1    RVPILGIPLIFDQHDNLVRMEAHGVAKIID-ITAMD-VQSLTNSLKNLLDPTEPYKQNMMKLSRIYRDQPIKPMDKAVFWIEFVMRHKGAGHLHTESYKL   487
m5e2    GVPILGIPLIFDQHDNLVRMEAHGVAEIID-ITTMD-VQSLTNSLKDLLDPTEPYKLNMIKLSRIYRDQPIKPMDKAVFWIEFVMRHKGAGHLRTESYQL   487
s5e1    GVPVLGIPLIFDQYDNMVRLKSRGVAEIVE-VTTLA-VESLTSSLKNILDPQKPYKQNMLKLSRLHHDKPMKPVDNALFWIEFVMRHKGAAHLRTESYKL   487
z5f1    GVPIVGLPLVFDQDYNLLKMKHKGVAKVLD-IATIN-RNIFKDALQEVLNDP-SYRSNMQKLSSLHKDTPLKPLDSALFWTEFVMRHRGAAHLRTDSYKM   479
f5f1    GVPIVGIPLVFDQADNLSRLRAKGVAKLLD-ISELD-SDIFQKAIEEVLNEP-LYALNMQRLSRLHRDAPMKPLDSALFWIEFVMRHKGAAHLRTESYRL   481
m5f1    GVPVVGIPIVFDQADNLSKLRVRGVARTVE-ISDLN-RTNFQEAIQDVLNDP-SYRMNMQRLSRLHRDQPMRPLDTALFWIEFVMRHKGAAHLRTESYKL   478
m5f2    GVPVVGIPIVFDQADNLSKLRVRGVARTVE-ISDLD-RTNFQEAIQDVLNDP-SYRMNMQRLSRLHRDQPMRPLDTALFWIEFVMRHKGAAHLRTESYKL   478
m5f4    GVPVVGIPIVFDQADNLSRLKARGVARTVE-ISDLD-RTNFQEAIQDVLNDP-SYRMNMQRLSRLHRDQPMRPLDTALFWIEFVMRHKGAAHLRTESYKL   481
s5f1    GVPIVGIPIVFDQADNLSRLRAKGVAEVMD-VSELN-TKNFLNAMQEVMNEP-SYRKNMQRLSRLHRDQPMKPLDKALFWMEFVMRHKGAAHLRTESYKM   481
z5g1    GVPVLALPLLFDQFDNVMRLQVRNAARVLQ-VATLT-SQEFLEGLKDVLENP-LYRSSIRKMSELHRDQPISPLDSATFWIEYVMRYKGAAHLRSEANNL   484
z5g2    GVPVLGLPLLFDQLDNIVRLQARGGARMLD-AATCS-TKEFLVALTDILENP-TYRQNMQKLSNLHRDRPLHPLDKAVYWIEFVLRNKGAPHLRAEAYNV   484
f5g1    GVPVVGLPLLFDQFDNLHRLKVRGAARLVE-AKSLT-KENFLEPLKDILETP-SYRDNIQRLSQLHRDQLMSPMDTAVFWTEYVIRNKGAAHLRAAGFTL   484
t5g1    GVPVLGLPLLFDQFDNLHRLKIRGAARVVE-AKSLT-KEDFLLALKDVLENP-SYRSNIKHLSRLHRDQLTSPMDTAVFWTEYVIRNKGAAHLRAAGFTL   484
m5g1    GVPVLGLPLLFDQFDNLLRLEVRGAARVVE-VRSLN-KENFHEALNDILNNP-TYRNNIQRLSQLHRDRLISPLETAIFWIEYVIRNRGAAHLQSRGFDL   486
s5g1    AVPVLGLPLLFDQFDNLLRLKVRGAARVVE-AKSIT-KEDFLEALRDILETP-SYRHNIQHLSQLHRDRPMSPMDTAIFWIEYVIRNKGAAHLQSAGFNL   484
s5g2    GVPVLGLPLLFDQFDNLLRLKVRGAARVVE-AKSIT-KEDFLEALRDILETP-SYRHNIQHLSQLHRDRPMTPMDTAIFWIEYVIRNKGAAHLQSAGFNL   484
f5h1    GVPMIGIPLLFDQFENVLRLEVRGAAKVVH-ATELT-RQKFLEVLQEVLTNP-SYRENMRHLSALHRDKPMHPLDTALFWIEFVMRHKGASHLRSESYRM   488
t5h1    GVPMIGIPLLFDQFENILRLEERGAAKVVH-ATELT-QQNFLEAVQEVLHDP-SYRENMRRLSALHRDKPMHPLDTALFWIEFVMRHKGASHLRTESFRM   488
s5h1    GVPIVGIPLLFDQFENILRLEARGAAKSVD-ATKLT-RENFMEAIQEVLQNP-SYRNNMKRLSALHRDKPMHPLETALYWIEFVMRHKGAAHLRTESYKM   487
x5a1    GVPIVGIPLFGDQFENILRLKTKGAAILLENLADIN-SEEIFSAVRLIIDDP-SYGAAMKHLSKLHRDTPIAPLNAAVFWTEFTMRHGGAGHLQAVGNNL   485
```

```
              *   *:
z5a1      SWIEYHSIDVILTLMVTGFVFALVIAYTIKYFCL-CVFRRK---VKRE---------------   525
z5a2      SWIEYQSIDVILTLMLMVLVFVLLTVYTMKYFCL-CLFRKK---VKRE---------------   524
z5a3      SWIEYQSIDVILTLMLMVLVFALVTAYMTKYFCL-CLFRKK---VKHE---------------   524
z5a4      SWIEYQSIDVILTLMLMVLVFALVTAYTTKYFCL-RLFRKK---VKRE---------------   525
z5a5      SWIEYHSIDVILTLLMILVLFCVLIYSIVKCICLKVVLKKK---VKTQ---------------   525
f5a1      NAIQYHSIDVLAFLVTLVLLIVAVFTSAVKFLWRRLFCRSK---VKKE---------------   526
f5a2      NAIQYHSIDVLAFLVTLVLLIVLVFTSAVKFLWRRLFCRSK---VKKE---------------   522
f5a4      NAIQYHSIDVLAFLVTLVLLIVAVFTSAVKFLWRRLFCRSK---VKKE---------------   523
f5a5      SWFTYHSLDVIAALSTAALLLVVICISALRCVWKILFR-TK---VKHE---------------   533
t5a1      SAIQYHSIDVLAFLLALVLLILAVFTAVLKFLWRRLFGRSK---VKRE---------------   526
t5a2      SAIQYHSIDVLAFLLTLVLLILAVFTAVLKFLWGRLFGRSK---VKKE---------------   526
m5a1      TMIQYYSMDVAAFLFATVLLFLLVSFSAVKFLIRQIFDRSK---VKKE---------------   526
s5a1      STIQYHSIDVVAFLLAVILLVFAVFVCAVMFLWRRVFSGSK---VKKE---------------   526
s5a2      SWFVYKSLDVIAALLAVILLVTFTCISIAGLLWRIILVGKK---VKHE---------------   534
z5b1      PWYSYHSVDVILVLISAVSLIILTIYAVIRYFCCGICMRTT---KVKLQ--------------   530
z5b2      LWYSYHSVDVILVLISAVSLIILSIYAVIRYFCCRICMRKT---KIKHE--------------   531
z5b3      PWYSYHSVDVILVLISAVSLIILSIYAVIRYFCCRICMRTT---KNKRE--------------   531
z5b4      PWYSYHSVDVILVLISAVSLIILTIYAVMRYFCCKICMRKT---KIKRQ--------------   531
f5b2      PWYSYHSVDVMLFFAGITLLIFMTFAALARCLCSRCVRAKS---KLD----------------   524
t5b3      PWYSYHSVDVVLFLTATVLLIILTIITVFKYLFTKVCKRKV---KHD----------------   524
t5b4      PWYSYHSVDVVLFLTATVLLIILTIVTVFKYLFTKVCKSKV---KHD----------------   524
t5b5      PWYSYHSVDVMLFLAGITLVISMIFVALIRCSCRRCVKTKS---KHD----------------   524
z5c1      PWYSYHSVDVSVTLIAVVLIFIYSMFVTVRYLCIKCCSRKR---KTE----------------   531
z5c2      PWYSYHSVDVVVTLFAVVLILTYCIFVTVRYLCVKCCSQKR---KTE----------------   531
z5c3      PWYSYHSVDVAVTLIAVVLIFIFSIFYVVRYVCIKCCSRKR---KTE----------------   531
f5c1      PWYSYHSVDVVLFLTGAVLLTILACTTMIRCLFTKICKRKV---KNE----------------   530
f5c2      PWYSYHSVDVMLFLAGITLLIFMTFAALVRCLCSRCVRAKS---KQE----------------   527
m5c1      PWYSYYSVDVVAFSFAVAAFIYFSLFAFLRWICFRKCLRRE---KQLQSTNKIGSHQAGLKQE   547
m5c2      PWYSYYSVDVVAFSFAVAAFIYFSLFAFLRWICFRKCLRRE---KQLQSTNKIGSHQAGLKQE   547
m5c4      PWYSYYSVDVVAFSFAVAAFIYFSLFAFLRWICFGKCLRRE---KQLQSANKIGSHKTGLKQE   547
m5c5      SWFSYHSADVVLFLTAAVLLVFLTSFVFIRCLFRAVCKCKV---KHE----------------   527
m5c7      SWFSYHSVDVVLFLTAAVLLVTLTSFMFIRCLFRAVCKHKV---KYE----------------   527
s5c1      PWYSYHSVDVMLF-LLTTAVVALLFVAGLLWSCFRLGCKRK---VKLD---------------   530
s5c2      PWYSYHSVDVVLFLAAAGLLVLLTIFLLIRCLCTAMCKHKM---KRD----------------   527
z5d1      PWYSYHSVDVFLVLFIVAAVCMLSTIAVIRYVCYKICCRRK---SKSE---------------   532
m5d1      PWYSYHSFDVVLLVLAVETVLLYAIYAVFRFLCCRRKRKTK---TKQN---------------   528
m5d2      PWYSYHSLDVVLLLLAVGAVLLYSIYGVFRFLCCRRKIKTK---TKLN---------------   528
z5e1      PWYSYHCLDVLALIMSLCTAAGLLLILISRALLRACVRKIK---AKKE---------------   532
f5e1      PWYAYHCLDVMAVFAAYGLILMSLVWVSCRCVIRALIRATK---SPAKSKKE-----------   536
t5e1      PWYAYHCLDVMAAFAALGLVVMALAWLLCRRVFRALTGAQK---KKSVNKSKKE---------   538
t5e2      PWYAYHCLDVMAAFAALGLVVMALAWLLCRRVFRALTGPKN---SKSQV--------------   533
m5e1      PWYAYQCLDVMAVFGAVGLFIFTFIWNSCRCMFRCLTIMKK---SKVE---------------   532
m5e2      PWYAYQCLDVMAVFGAVGLFIFTFIWISCRCMFRCLTRMKK---SKVE---------------   532
s5e1      PWYAYHCLDVMAVFVLFAVLITALVWVSFRCLIKRLIRTKK---TTAKSKHD-----------   536
z5f1      PWYSYHSVDVGVFLVSVLCFIVFVLFKIVRCLCCRFCAKRN---RAKQD--------------   525
f5f1      PWYSYHSVDVLLFLIAVVFLVLLGFVVLV-WSCFRPCVKR----KEKSD--------------   525
m5f1      PWYSYHSVDVIMFLLAAAVLLLFSIFGFV-WFCFRLCVQR----KQKCD--------------   522
m5f2      PWYSYHSVDVIVFLLAAAVLLLFSIFGFL-WFCFRLCVQR----KQKCE--------------   522
m5f4      PWYSYHSVDVIVFLLAAAVLLLFSIFGFL-WFCFRLCVKR----KQKCD--------------   525
s5f1      PWYSYHSVDVMLFLLAITVVALLFIAALL-WSCFRLCCKR----KVKLD--------------   525
z5g1      AWYSYHSLDVLVFLLAIAVIAFWTSVYVCRFVCCRKSIKK----RKVD---------------   528
z5g2      SEFSYYCLDVVALVLFMLLGTLGGVYTICKRRPKRQENKTGGDWNKKGLK-------------   534
f5g1      PWYTYYSLDVALAISAVIAACAWAFVFICRVLCCRKSRRK----TKAD---------------   528
t5g1      PWYTYHSLDVAAVATAVIGACVWVVVFICRVLYRRMSRRK----AKSD---------------   528
m5g1      PWYSYFCLDVAAFFVTISGAFIWVSVSVCRFLCCRKSRRK----TKAE---------------   530
s5g1      PWYSYFCLDVAVLFGALIWVFIWASVVVYKILCCRGRRRK----IKAE---------------   528
s5g2      PWYSYFCLDIAVLFGALIGVFVWASVVVCKILCCRGRRRK----IKAE---------------   528
f5h1      PWYSYHSVDVGGFALAVAFMMTVFVVGSLKFLCFSRKK-KS---KQD----------------   531
t5h1      PWYSYHSVDVGSLVLAVVGTLAGFVVGSVKFLCFSRKKNQS---KISDIR-------------   535
s5h1      PWYAYYSVDVICFLVAVLLMLTAVIVGSIRFVCFRLCRRRK---TKKE---------------   532
x5a1      PWYQYYLLDVIAFLAFIAIICISLLYKFLKAIFRKCCSRKK---KNKRD--------------   531
```
